# Comparing the Performance of Double‐Stranded and Single‐Stranded DNA Libraries for Ancient Oral Microbiome Reconstruction

**DOI:** 10.1111/1755-0998.70201

**Published:** 2026-09-21

**Authors:** Keri Burge, Irina M. Velsko, Domingo C. Salazar‐García, María Haber Uriarte, Joaquín Lomba Maurandi, Christina Warinner

**Affiliations:** ^1^ Department of Anthropology Harvard University Cambridge Massachusetts USA; ^2^ Department of Archaeogenetics Max Planck Institute for Evolutionary Anthropology Leipzig Germany; ^3^ Associated Research Group of Archaeogenetics Leibniz Institute for Natural Product Research and Infection Biology, Hans Knöll Institute Jena Germany; ^4^ Departament de Prehistòria, Arqueologia i Història Antiga Universitat de València València Spain; ^5^ Department of Geological Sciences University of Cape Town Cape Town South Africa; ^6^ Departamento de Prehistoria, Arqueología, Historia Antigua, Historia Medieval y Ciencias y Técnicas Historiográficas Universidad de Murcia Murcia Spain; ^7^ Faculty of Biological Sciences Friedrich Schiller University Jena Germany; ^8^ Department of Human Evolutionary Biology Harvard University Cambridge Massachusetts USA

**Keywords:** ancient DNA, dental calculus, metagenomics, oral microbiome

## Abstract

DNA library construction methods can affect the recovery of ancient DNA, thus influencing downstream analyses. While single‐stranded library preparation (ssLib) has been shown to outperform double‐stranded (dsLib) for highly degraded vertebrate host DNA, especially for samples older than 40,000 years, few studies have examined how library protocols shape ancient microbial community reconstruction. Here, we compare the sequencing output of paired ssLib and dsLib dental calculus libraries generated from 12 Neanderthals and two Chalcolithic humans, prepared using implementations of the Gansauge et al. and Meyer and Kircher protocols, respectively, and sequenced with identical Illumina chemistry. We compared read length and GC%, read duplication and taxonomic profiles across normalization strategies to assess protocol‐specific biases. Double‐stranded libraries retained a significantly higher proportion of sequenced reads throughout data processing (dsLib 72.1%, ssLib 37.9%), a higher proportion of oral reads (dsLib 9.78%, ssLib 6.75%), significantly longer median oral DNA read lengths (dsLib 57.5 bp, ssLib 50.5 bp) and more GC‐rich fragments (dsLib 60.5% GC, ssLib 52.5% GC). In contrast, ssLibs exhibited slightly higher Shannon diversity and a greater proportion of unique reads. Despite these differences, species richness and overall community composition was not significantly different between protocols, with individual and preservation status explaining the most variance. Stratifying reads by length (< 50 bp vs. ≥ 50 bp) resulted in different classification rates but only had minor effects on diversity estimates. Together, these results demonstrate that dsLib and ssLib protocols impose distinct trade‐offs and library choice should be guided by study‐specific goals.

## 1 Introduction

Recovery of authentic ancient DNA (aDNA) from archaeological samples is allowing reconstruction of the past genomic landscapes of animals, plants and microbes. However, the study of ancient DNA is challenged by distinct damage patterns that accumulate postmortem, including DNA fragmentation leading to short molecule lengths and cytosine deamination resulting in an observed accumulation of miscoding lesions near the ends of molecules (Bokelmann et al. 2020; Dabney, Meyer, and Pääbo 2013; Hofreiter et al. 2001; Sawyer et al. 2012; Ziesemer et al. 2015). These damage patterns are both a boon and a bane of the field of paleogenomics, providing characteristics for authentication against recent contamination (Briggs et al. 2007; Hofreiter et al. 2001) but also decreasing molecule recoverability. Advances and improvements in sample processing facilities, DNA extraction methods (Dabney, Knapp, et al. 2013; Rohland et al. 2018; Rohland and Hofreiter 2007) and library construction protocols (Carøe et al. 2018; Gansauge et al. 2017; Gansauge and Meyer 2013, 2019; Kapp et al. 2021; Kircher et al. 2012; Meyer et al. 2012; Meyer and Kircher 2010) over the last 20 years have substantially elevated the quality and quantity of aDNA recovery. However, the focus of these efforts has been on the recovery of vertebrate host DNA, and the recovery of ancient microbial DNA—including that from complex microbiota such as host microbiomes (Warinner et al. 2017)—has not yet received the same level of testing or optimization.

Biological remains differ substantially in initial cell density and substrate structure, which affects the amount of ancient vertebrate host DNA and microbial DNA that is likely to be recovered. In skeletal tissue, vertebrate host DNA content is typically very low (< 2 ng/mg), while the highly complex microbial communities of secondary substrates such as dental calculus (calcified dental plaque) and paleofeces yield substantially higher amounts of DNA, with a reported median biomass of 39 ng/mg (SD = 62.5) (Fagernäs et al. 2020; Velsko, Hübner, et al. 2026). Further, the high porosity of skeletal tissues (bone and dentine) permits microbial infiltration during decay and burial, leading to high amounts of the recovered DNA being derived from environmental microorganisms, rather than the host organism itself (Javan et al. 2016; Mann et al. 2018; Metcalf et al. 2016; Warinner et al. 2014, 2015). Because endogenous host DNA represents only a small fraction of total DNA, vertebrate host‐focused studies have sought to maximize the recovery of short, damaged fragments that have characteristics of authentic ancient molecules since longer reads are more often attributed to contaminants or environmental DNA (Dabney, Knapp, et al. 2013; Fu et al. 2014; Mafessoni et al. 2020; Meyer et al. 2012, 2014, 2016; Prüfer et al. 2014, 2017; Slon et al. 2017). In comparison, microbial‐focused studies begin with inherently high microbial biomass but face the interpretive challenge of distinguishing ancient microbes from postmortem colonizers, a task that depends heavily on confident taxonomic assignment (Warinner et al. 2017).

Vertebrate host‐focused studies benefit from the ability to map reads to a single, well‐characterized reference genome, which allows even short, damaged fragments to be confidently assigned (Dabney, Knapp, et al. 2013; Fu et al. 2014; Mafessoni et al. 2020; Meyer et al. 2012, 2014, 2016; Prüfer et al. 2014, 2017; Slon et al. 2017). In contrast, microbial studies must map against expansive and heterogenous reference databases of thousands of taxa (Warinner et al. 2017). In this context, taxonomic assessment is most strongly influenced by DNA fragmentation (Velsko et al. 2018). Reads shorter than 30 bp are usually discarded due to lack of taxonomic specificity and even reads between 30 and 50 bp may lack the resolution for reliable microbial classification (Eisenhofer and Weyrich 2019). Short reads can also introduce taxonomic bias, particularly among taxa with variable GC content (Mann et al. 2018) or large accessory genomes (Chaves‐Moreno et al. 2015; Kuo et al. 2009; Sela et al. 2016; Tettelin et al. 2005), where substantial genomic divergence may occur within a single species. For instance, in the species *Porphyromonas gingivalis* , the number of genes between strains can vary by 22% (ranging from 1870 to 2405 genes) yet still be classified as the same species (Koehler et al. 2003; Warinner et al. 2017). Therefore, in comparison to vertebrate host‐focused studies, microbial paleogenomic studies of mixed microbial communities necessitate longer reads to achieve sufficient taxonomic resolution for genome reconstruction and analysis.

Because ultrashort DNA is abundant and still informative in vertebrate host‐focused paleogenomics, methodological innovation in aDNA research has largely centred on maximizing the recovery of very short DNA fragments. Extraction protocols have been modified through altered binding chemistries (Rohland and Hofreiter 2007), buffer compositions (Dabney, Knapp, et al. 2013) and size‐selection strategies (Rohland et al. 2018) to preferentially retain ultrashort endogenous molecules that would be lost under standard recovery conditions. During library preparation, these priorities are further reinforced: although double‐stranded DNA libraries (dsLibs) remain comparable to standard commercial Illumina workflows (Dubbeldam 2023), paleogenomics has increasingly adopted single‐stranded library (ssLib) methods, which more efficiently capture highly fragmented molecules.

In dsLib preparation protocols following the Meyer and Kircher (2010) approach, double‐stranded aDNA molecules are blunt end‐repaired and ligated to double‐stranded adapters (Briggs et al. 2007; Kircher et al. 2012; Meyer and Kircher 2010). However, this blunt‐end ligation of sequencing adapters is non‐directional, meaning that either of the two adapters can be added to each end of the sequencing molecule. Since only molecules that have one of each adapter in the correct orientation can be sequenced, approximately 50% of DNA molecules are lost due to incompatible adapter combinations (Meyer and Kircher 2010). Therefore, some unique, original DNA fragments will not enter the sequencing library. This method is also highly sensitive to unrepaired nicks in the initial fragments, which cause further downstream DNA losses as these molecules may not be amplifiable. Additional size selection‐based purification steps, such as with carboxyl‐coated magnetic beads or silica spin columns, may further result in the loss of short molecules (Meyer and Kircher 2010).

Single‐stranded library protocols (Gansauge et al. 2017, 2020; Gansauge and Meyer 2013, 2019; Meyer et al. 2012) modify dsLib protocols to increase endogenous DNA recovery by heat denaturing all input molecules into a single‐stranded form prior to adapter ligation. DNA strands are ligated to a biotinylated adapter, immobilized on streptavidin‐coated beads, copied and converted into amplifiable libraries (Gansauge et al. 2017, 2020; Gansauge and Meyer 2013). Melting the DNA prior to library construction eliminates the problem of unrepaired nicking, and because molecules remain bound during processing, ssLibs minimize DNA loss during purification steps (Gansauge and Meyer 2013). As a result, ssLibs have been shown to be more effective in maximizing the recovery of short, damaged endogenous DNA fragments (Bennett et al. 2014; Wales et al. 2015) from samples older than 40,000 years (Gansauge et al. 2020; Meyer et al. 2016).

Despite its advantages, optimization of library protocols for ultrashort fragment recovery raises important practical considerations for microbial paleogenomics. Although ssLibs recover more aDNA, it remains unclear whether these predominantly ultrashort reads can be effectively incorporated into microbial metagenomic analyses. Conversely, dsLibs contain longer DNA, but the process of preparation may result in substantial molecule loss, potentially altering the relative abundance and composition of recovered taxa. Thus, library choice may shape not only total DNA yield but also the taxonomic profile inferred from ancient microbiome samples. These uncertainties are compounded by incomplete reference databases and the growing reliance on metagenome‐assembled genomes (MAGs), which require sufficient read length for accurate assembly (Klapper et al. 2023; Wibowo et al. 2021). As yet, relatively few ancient microbiome studies utilize ssLibs (Klapper et al. 2023; Maixner et al. 2021) and whether either library preparation strategy consistently outperforms the other for ancient microbiomes is unclear. A recent study (Wright et al. 2025) on six dental calculus samples approximately 4000–7500 years old found that sample preservation significantly biassed the effectiveness of each protocol and no library protocol outperformed the other across the assessed metrics. Larger studies with greater time depth are needed to rigorously test whether these findings hold up more broadly and especially for older and more damaged samples.

Dental calculus provides an ideal system for evaluating the performance of library preparation strategies on reconstructing ancient microbiota, as it preserves oral microbiomes over deep time, typically contains a mixture of ~50–200 oral species per sample, is well characterized in both modern and ancient contexts and spans a wide range of preservation states (Akcalı and Lang 2018; de La Fuente et al. 2013; Fellows Yates, Velsko, et al. 2021; Velsko et al. 2019, 2023, 2024; Warinner et al. 2014; Weyrich et al. 2017). Here, we present a systematic comparison of ssLib and dsLib preparation protocols for ancient oral microbiome analysis using dental calculus samples from twelve Neanderthals (aged ~43,000–102,000 BP) and two Chalcolithic humans (aged ~4200 BP). To understand how library protocols influence multiple stages of ancient metagenomic analysis, we assessed library read complexity and contamination, read processing and retention through merging and quality filtering, fragment length and GC content and both alpha and beta diversity of inferred microbial communities. We find that while dsLibs retain more reads throughout data processing and recover longer DNA fragments, both dsLibs and ssLibs yield largely comparable species richness and community composition, with the main difference being that dsLibs have higher GC%, suggesting they are depleted in AT‐rich fragments. Overall, we find that individual and preservation state explain substantially more genetic variation than library preparation strategy, indicating that library protocol selection should be driven by study‐specific goals.

## 2 Materials and Methods

### 2.1 Samples

**FIGURE 1:**
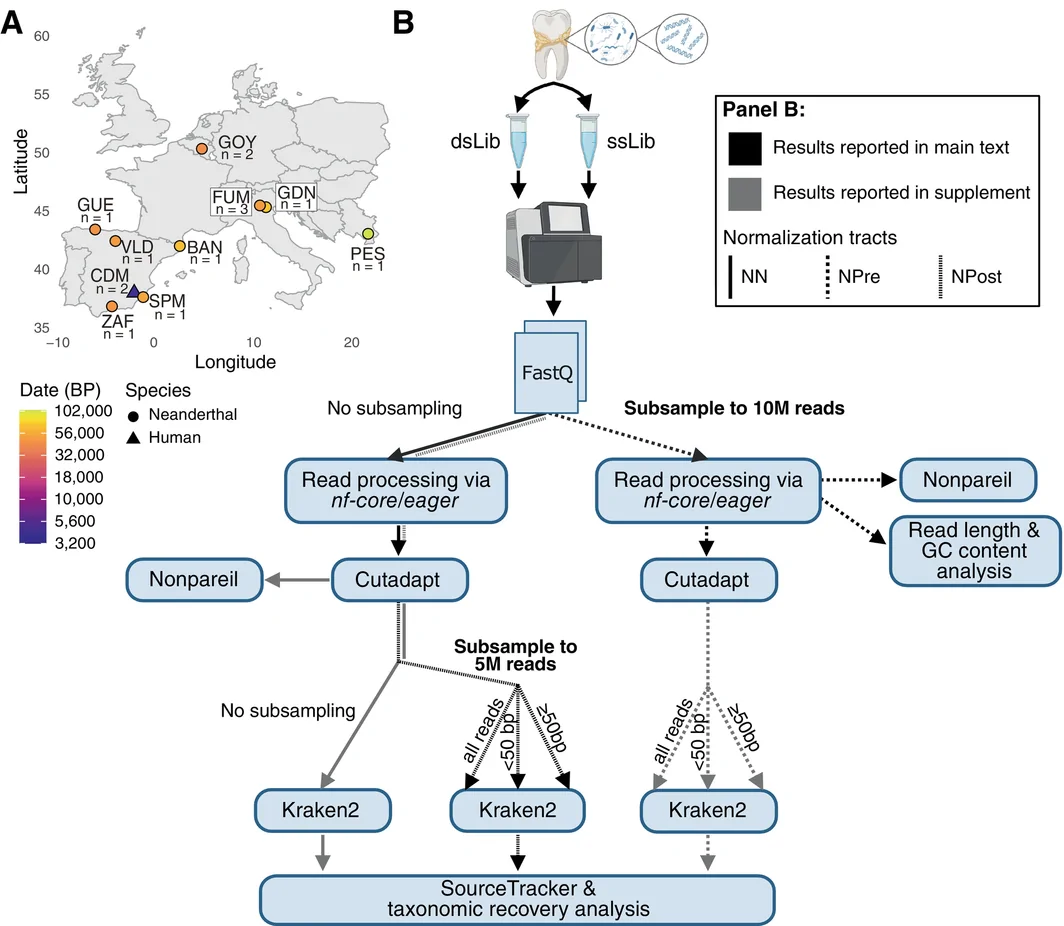
Experimental design and library metadata. (A) Origin of dental calculus samples used in this study. Site abbreviations are shown above each point along with the number of individuals sampled from that site (BAN, Banyoles; CDM, Camino del Molino; FUM, Fumane; GDN, Grotta De Nadale; GOY, Goyet Cave; GUE, La Güelga; PES, Pešturina; SPM, Sima de las Palomas del Cabezo Gordo; VLD, Cueva de Valdegoba; ZAF, Cueva del Boquete de Zafarraya.). Points are coloured by age (BP), and shape denotes the sample host. (B) Experimental design diagram. Both dsLibs and ssLibs were generated from a single extract from each calculus sample. Different normalization tracts (indicated by line type) were utilized to control for outstanding factors. Grey lines indicate analyses reported in the supplement while black lines indicate results reported in the main text. Experimental design diagram (B) created in BioRender. Burge, K. (2026) https://www.BioRender.com/4y5enah.

This study included 12 previously published Neanderthal dental calculus samples, and two unpublished dental calculus samples from Chalcolithic humans (Figure 1A). The 12 Neanderthal dental calculus samples originate from five sites in Spain (Banyoles (BAN, ~66 kya) (Grün et al. 2006), La Güelga (GUE, ~44 kya) (Menéndez et al. 2018), Cueva de Valdegoba (VLD, ~48 kya) (Dalén et al. 2012), Cueva del Boquete de Zafarraya (ZAF, ~46 kya) (Sanchez 1999; Wood et al. 2013) and Sima de las Palomas del Cabezo Gordo (SPM, ~55 kya) (Trinkaus and Walker 2017; Walker et al. 2008, 2010)), one site in Serbia (Pešturina Cave (PES, ~102 kya) (Radović et al. 2019)), two sites in Italy (Fumane (FUM, ~48 kya; *n* = 3) (Benazzi et al. 2014), Grotta de Nadale (GDN, ~70 kya) (Arnaud et al. 2017)) and one site in Belgium (Troisième caverne of Goyet (GOY, ~43 kya; *n* = 2) (Rougier et al. 2016)). Sampling methods and permissions are described in Fellows Yates, Velsko, et al. (2021). The two Chalcolithic human samples are from the site of Camino del Molino in Spain (CDM, ~4.2 kya; *n* = 2) (Lomba Maurandi et al. 2009). This site was excavated by the University of Murcia in 2008, revealing a collective burial pit with a minimum of 1300 individuals dated to the second half of the third millennium BCE.

Previously published sequencing data is available on the European Nucleotide Archive (ENA) (PRJEB34569) and the samples are listed in the AncientMetagenomeDir (Fellows Yates, Andrades Valtueña, et al. 2021). Accessions are listed in Table S1A. Newly published sequenced data is available on the ENA (PRJEB112518) and accessions are listed in Table S1B.

### 2.2 DNA Extraction and Next Generation Sequencing

The DNA extracts used in this study were produced in Fellows Yates, Velsko, et al. (2021) following a silica‐based total DNA extraction protocol modified by Dabney, Knapp, et al. (2013) and adapted for dental calculus by Mann et al. (2018), as documented in the corresponding bench protocol from Aron, Hofman, et al. (2020). Sequencing data for double‐stranded libraries (dsLibs) and single‐stranded libraries (ssLibs) were previously generated for 12 samples (Fellows Yates, Velsko, et al. 2021) without uracil‐DNA glycosylase (UDG) treatment, and the same DNA extraction and library build protocols were used for two additional samples, CDM019 and CDM020. Libraries constructed using the dsLib method were prepared following an optimized implementation of the Meyer and Kircher (2010) protocol for aDNA library construction (Aron, Neumann, et al. 2020) and indexing (Stahl et al. 2023), with 20 μL of input DNA extract per sample. The Aron, Neumann, et al. (2020) dsLib construction protocol follows the same overall workflow as Meyer and Kircher (2010), including blunt‐end repair, purification, adapter ligation and adapter fill‐in. However, unlike Meyer and Kircher (2010), which was developed for high‐throughput library preparation in a 96‐well format, Aron, Neumann, et al. (2020) presents a manual workflow using PCR strip tubes and individual 1.5 mL LoBind tubes for purification. The primary methodological differences involve updated commercial buffer formulations (Quick Ligase Buffer and Isothermal Buffer), modified reaction volumes and substitution of the final purification after adapter fill‐in with an 80°C heat‐inactivation step before PCR. The Stahl et al. (2023) indexing protocol varies from the Meyer and Kircher (2010) protocol through the use of the uracil‐tolerant enzyme Pfu Turbo Cx HotStart DNA Polymerase rather than the proofreading enzyme Phusion Hot Start High‐Fidelity DNA Polymerase, which stalls when encountering uracils.

Sequencing data for dsLibs was previously generated for BAN001, FUM001, FUM002, FUM003, GDN001, GUE001, SPM002, VLD001 and ZAF001 on an Illumina NextSeq 500 with paired‐end 75 bp chemistry (Fellows Yates, Velsko, et al. 2021). For samples GOY005, GOY006, PES001, CDM019 and CDM020, additional dsLib sequencing data was generated from the original libraries on an Illumina NextSeq 500 with paired‐end 75 bp chemistry to generate sufficient reads for analysis.

The libraries constructed using the ssLib method were prepared following an implementation of the Gansauge et al. (2017, 2020) ssLib preparation method for both library construction (Nagel et al. 2026) and indexing (Nagel et al. 2025), with 30 μL of input DNA extract per sample. Whereas the original protocol was designed to process up to 16 libraries per run, the Nagel et al. (2025, 2026) implementation is optimized for automated liquid handling on the Bravo NGS Workstation B in a 96‐well format. The workflow retains the same core steps of dephosphorylation, splinted ligation of the first adapter, immobilization of ligation products on magnetic beads, bead washing, second‐strand synthesis, a second bead wash, ligation of the second adapter, a third bead wash and library elution. The same reagents are used, with reaction setup and handling adapted for automated liquid processing.

New sequencing data was generated for the ssLibs of all 12 published Neanderthal samples and the two unpublished CDM samples to increase read depth to be comparable to the previously sequenced dsLibs. Sequencing of the ssLibs was performed on both an Illumina NextSeq 500 and an Illumina HiSeq 4000 with paired‐end 75 bp chemistry. All samples were sequenced at the MPI‐EVA Core Facility with a target depth of 10 million paired reads. Controls included extraction blanks and library‐build blanks, which were sequenced on a NextSeq 500 or a HiSeq 4000 (Table S1C).

### 2.3 Comparative Data

Additional publicly available datasets were downloaded to aid in estimating endogenous and contaminant sources of DNA in the ancient dental calculus. Human‐derived sources include skin (Oh et al. 2016), gut (Obregon‐Tito et al. 2015), modern calculus (Velsko et al. 2019) and modern dental plaque (Human Microbiome Project Consortium 2012). Sediment metagenomes were from a study on DNA from Late and Middle Pleistocene sediments in Eurasia (Slon et al. 2017). Sequencing data from 11 published archaeological bone samples (Jeong et al. 2018), which were re‐sequenced in Fellows Yates, Velsko, et al. (2021), were used as archaeological environment proxy controls (Table S1D) to identify possible contamination from burial soil, excavation and handling. All data were downloaded from the ENA.

### 2.4 Data Processing

To control for variation in the number reads available for analysis at different steps, three approaches were taken: (1) no normalization (NN), (2) normalizing to 10 million read pairs prior to read processing (NPre) and (3) normalizing to 5 million reads (merged pairs) post read processing (using approach 1 as the input) and prior to taxonomic identification (NPost) (Figure 1B). For the NN approach, no normalization was applied at any step. For the NPre approach, all libraries were first normalized to 10 million reads. Subsampling was performed using Seqtk v1.2‐r94 (https://github.com/lh3/seqtk) with the command seqtk sample ‐s100 input.fastq.gz 10000000. Subsampled R1 and R2 reads were subsequently processed following the pipeline described below before continuing to taxonomic classification. For the NPost approach, the post‐processing FASTA files from the NN dataset were subsampled to 5 million reads using Seqtk v1.2‐r94 prior to taxonomic classification. Four samples did not have at least 5 million reads after processing, FUM001, GUE001, SPM002 and ZAF001, and were excluded from further downstream analysis in this tract.

To assess the impact of read length on taxonomic assignment, read length stratification was performed on both the NPre dataset and the NPost dataset prior to taxonomic profiling (Figure 1B). Post‐processing reads were stratified into two groups: < 50 and ≥ 50 bp. FASTA files were subsampled from the post‐processed FASTA using Seqkit v2.4.0 (Shen et al. 2024) with the commands seqkit seq ‐m 1 ‐M 49 and seqkit seq ‐m 50, respectively. This produced three separate subdatasets within the NPre and the NPost datasets: (1) all reads, (2) < 50 bp reads and (3) ≥ 50 bp reads. These read length–stratified datasets were independently taxonomically profiled as described in Section 2.6, Taxonomic Profiling and Cleanup. In total, 7 datasets were taxonomically profiled: (1) no normalization, (2) NPre all reads, (3) NPre reads < 50 bp, (4) NPre reads ≥ 50 bp, (5) NPost all reads, (6) NPost reads < 50 bp and (7) NPost reads ≥ 50 bp.

All sequencing data produced for this study, as well as the published datasets, were processed with the *nf‐core/eager* pipeline v2.4.6 (Fellows Yates, Lamnidis, et al. 2021). Separate *nf‐core/eager* runs were performed for each library type (dsLib, ssLib). This consisted of quality checks with FastQC v0.11.9 (Andrews 2010), adapter trimming, read merging and quality filtering with AdapterRemoval v2.3.2 (Schubert et al. 2016), removing reads < 30 bp and mapping against the human genome (hs37d5) and other sequencing/laboratory artefacts with ‐bwaalnl 16500 \ ‐bwaalnn 0.01 \ ‐bwaalno 2 parameters (v0.7.17‐r1188) (Li and Durbin 2009) and samtools v1.12 (Danecek et al. 2021). The mapping FASTA file contained the human genome as well as other sequencing and laboratory artefacts and truncated adapter sequences that were not being properly removed with AdapterRemoval (Table S1E). All reads that did not map to the human genome and additional artefacts were then further cleaned up using Cutadapt v1.18 (Martin 2011). Separate Cutadapt runs were performed for each library type with their associated forward and reverse adapter sequences as well as truncated adapter sequences that persisted after the *nf‐core/eager* pipeline using the minimum length ‐m 30 parameter. Read stratification as described above was performed on reads post‐Cutadapt.

### 2.5 Read Complexity

After processing via *nf‐core/eager* and Cutadapt, FASTQ files were used as input for Nonpareil v.3.304 (Rodriguez‐R et al. 2018; Rodriguez‐R and Konstantinidis 2014). Nonpareil predicts diversity in metagenomic datasets by using the principle that datasets with more coverage have a higher proportion of redundant reads. To calculate this, Nonpareil generates a sample of query reads from the entire dataset and then sees the number of matches to that query dataset within the whole dataset. Curves were plotted in R v4.3.2 (R Core Team 2023) with the Nonpareil library following the Nonpareil documentation.

### 2.6 Taxonomic Profiling and Cleanup

The Cutadapt output FASTQ files were used as an input for the taxonomic profiler Kraken2 v2.1.2 (Wood et al. 2019) with the GTDB r207 database for taxonomic classification (downloaded from the Struo2 ftp server (Youngblut and Ley 2021); http://ftp.tue.mpg.de/ebio/projects/struo2/GTDB_release207/kraken2/) and a confidence threshold of 0.15 (Wright et al. 2023). Kraken2 species‐level taxonomic tables were cleaned by first applying a 0.005% relative abundance filter. To identify and remove potential laboratory and environmental contaminant species from our ancient calculus datasets, the R package decontam (Davis et al. 2018) was used with the prevalence method and a threshold of 0.9. To identify laboratory contamination, we used the extraction and library controls from this study. To identify environmental contaminant species, we used the archaeological bone samples. Contaminants were then removed from the Kraken2 species tables.

In R, strain‐specific suffixes appended to species names were removed to collapse classifications to the species level, as GTDB average nucleotide identity cut‐offs to delineate species artificially split numerous oral species into smaller units (Galtier et al. 2026; Parks et al. 2020, 2022). Read counts were then summed by species. To reduce the impact of low‐abundance and potentially spurious assignments, species with fewer than 10 reads were treated as absent by setting their counts to zero.

### 2.7 Sample Quality and Preservation

In order to assess environmental contamination, microbial sources were checked with the R package cuperdec (Fellows Yates, Velsko, et al. 2021). This package generates cumulative percent decay (cuperdec) curves, which visualize the level of endogenous oral microbial content in metagenome samples. To do this, the software determines what percentage of species in the sample derives from an oral source at each abundance rank. To establish the correct target source, a list of oral species (following the GTDB naming scheme) was created by referencing the Human Oral Microbiome Database (HOMD) v4 (Escapa et al. 2018). Because the GTDB r207 database used for taxonomic profiling has updated unnamed species that have yet to be added to HOMD, we looked at the isolation source of unnamed species within the genera of known oral species to confirm whether they are likely attributed to the oral microbiome (Table S1F). To run cuperdec, an adaptive burn‐in filter with a threshold of 50% was applied to the curves to determine which samples are well‐preserved and which are poorly preserved.

Overall preservation and potential sources of contamination were further assessed using SourceTracker v1.0.1 (Knights et al. 2011). Species‐level taxonomic count tables generated with Kraken2, including samples from potential sources (sediment, skin, gut, modern dental plaque and modern dental calculus), were used as input, after prior decontamination via the R package decontam and filtering to collapse strain specific suffixes and remove spurious hits. SourceTracker was run in R using the QIIME‐compatible implementation, with samples rarefied to 1900 reads for model training and 51,500 reads for sink estimation. Source contribution estimates were generated for each library, and verbose output was retained for quality control. Results were visualized in R using ggplot2 (Wickham 2016) and ggpattern (https://github.com/trevorld/ggpattern). We considered a sample to be well‐preserved if over 50% of the sample was composed of oral sources (modern calculus and plaque). Regardless of preservation status, all samples were included in downstream analysis unless otherwise stated.

To assess sample similarity to sources (sediment, gut, skin, modern dental plaque and modern dental calculus) while minimizing the influence of sequencing depth on community structure, we performed principal coordinates analysis (PCoA) on a Jaccard distance matrix calculated from rarefied species‐level taxonomic profiles. This method was selected as an initial principal components analysis (PCA) of species‐level taxonomic abundance data showed that sample clustering was strongly influenced by differences in sequencing depth.

In R, species count tables generated with Kraken2 were combined across ancient calculus libraries and potential source environments (samples described in Section 2.3, Comparative Data) and samples with zero counts after collapsing classifications to the species level and applying the low‐abundance filter described in Section 2.6 (Taxonomic Profiling and Cleanup) were excluded. The resulting species table was converted to a phyloseq (McMurdie and Holmes 2013) object and rarefied to the minimum sample depth using random subsampling with a fixed seed to ensure reproducibility using the phyloseq rarefy_even_depth() function. Pairwise sample dissimilarities were calculated using Jaccard distance based on presence–absence information with vegdist() from the package vegan (Oksanen et al. 2025), which emphasizes compositional similarity while reducing sensitivity to read count biases. Ordination was performed using PCoA with the pcoa() function in the package ape (Paradis and Schliep 2019) and plotted with ggplot2. To test whether the environment significantly structured community composition, a permutational multivariate analysis of variance (PERMANOVA) was performed on the Jaccard distance matrix with the function adonis2() from the R package vegan (Oksanen et al. 2025).

### 2.8 Alpha Diversity

Alpha diversity was compared for both the total species profiles as well as the oral‐associated taxa profiles. The total species profiles consist of the species‐level Kraken2 output tables after collapsing strain specific suffixes, removing spurious hits and running decontam. The oral‐associated taxa profiles consist of these tables subset in R to include only the oral species listed in Table S1F. To quantify the number of observed species, the total species and oral species Kraken2 output tables were used to calculate the total number of unique species detected per library in R. Species counts were computed both before filtering for oral‐associated taxa (total observed species) and after restricting profiles to only oral‐associated taxa. To calculate the Shannon index, the diversity() function in the R package vegan (Oksanen et al. 2025) was used. In parallel, the number of reads classified by Kraken2 and the number of reads assigned to oral taxa were calculated for each library, allowing estimation of the proportion of oral reads relative to both total input reads and classified reads. With the R package rstatix (Kassambara 2023), species diversity values were compared between library preparation protocols using paired Wilcoxon signed‐rank tests with a FDR *p* value adjustment, and effect sizes were estimated using rank‐biserial correlation. Raincloud plots for both observed species and the Shannon index were created in R following a tutorial from Cedric Scherer (https://www.cedricscherer.com/2021/06/06/visualizing‐distributions‐with‐raincloud‐plots‐and‐how‐to‐create‐them‐with‐ggplot2/) with the R packages ggplot2 (Wickham 2016), ggforce (Pedersen 2025), ggdist (Kay 2024, 2025), gghalves (https://github.com/erocoar/gghalves) and ggpubr (Kassambara 2025).

### 2.9 Beta Diversity

To explore beta diversity, analyses were performed on the Kraken2 species table after running decontam and removing spurious hits. Similar to alpha‐diversity, beta‐diversity metrics were computed for both total observed species and after restricting profiles to only oral‐associated taxa. A value of +1 was added to all species read counts, which were then CLR‐transformed to perform a PCA on a Euclidean distance matrix using the R package mixomics (Rohart et al. 2017). Plots were generated with ggplot2 (Wickham 2016). To determine if the library groups have significantly different species profiles, a PERMANOVA was performed on the Euclidean distance matrix with the function adonis2() from the R package vegan (Oksanen et al. 2025).

To identify taxa contributing most strongly to ordination structure, we examined principal component loadings from the PCA of species‐level relative abundance profiles. For each principal component, taxa were ranked by their loading values, and the 10 taxa with the strongest positive loadings and the 10 taxa with the strongest negative loadings were retained as the most influential contributors to that axis. These taxa were visualized on PCA biplots as vectors originating at the plot origin, with vector direction and magnitude corresponding to the sign and strength of the loading on the plotted PC axes. The vegan function betadisper() was additionally utilized to analyse group dispersion (Oksanen et al. 2025).

### 2.10 Read Characteristics—Read Length and GC Content

To investigate variation in read length and GC content between libraries, we computed per‐sample read count distributions across fragment length and GC‐content bins. Read count distributions across fragment length and GC‐content bins were calculated at selected points throughout the workflow. Read length and GC histograms were created for the read processing normalized dataset (NPre) in order to better understand how these variables are affected during key read processing steps (adapter removal and merging, removing reads < 30 bp, removing lab and sequencing artefacts and Cutadapt).

Further, for all normalization approaches, read length and GC analyses were performed on all classified Kraken2 reads and oral Kraken2 reads. To isolate classified Kraken2 reads, headers from the Kraken2 output file indicative of a classified read were identified and then only those reads were pulled from each sample's FASTQ file using Seqkit. Read characteristics were summarized via EMBOSS infoseq (https://emboss.sourceforge.net/apps/cvs/emboss/apps/infoseq.html). The same methodology was used for oral reads, except to isolate the oral reads, species names had to first be correlated with their associated Kraken2 taxonomy IDs before filtering the FASTQ files. These distributions were visualized as histograms, with per‐library median values overlaid. Library‐level medians were further summarized using boxplots to enable comparison between protocols.

### 2.11 Species‐Specific Postmortem Damage

To assess whether reads assigned to specific taxa exhibited molecular damage patterns consistent with aDNA, we performed species‐specific read mapping followed by postmortem damage analysis. Reads from the NPost dataset were mapped against reference genomes for *Actinomyces dentalis* (GCF_000429225.1), *Aggregatibacter kilianii* (GCF_963519225.1), *Flexilinea* sp001717545 (*Anaerolineaceae* bacterium oral taxon 439) (GCA_001717545.1), *Lautropia mirabilis* (GCF_900637555.1), *Methanocatella oralis* (GCA_001639275.1) and *Neisseria elongata* (GCF_900453895.1). These species were selected because they contributed strongly to ordination structure in the PCA of well‐preserved samples and span a range of genomic GC content.

Mapping was performed separately for each species. Reads were first aligned using BWA align (v.0.5.10‐evan.10) (Li and Durbin 2009) with the parameters ‐n 0.01 ‐o 2 ‐l 16500. A SAM file was generated with BWA samse. This output was converted to a BAM file and filtered using Samtools view v1.18, with the parameters ‐Sb ‐q 25 ‐F 4 ‐e ‘qlen >=30’. Duplicated reads were removed using Samtools rmdup (Li et al. 2009). Last, the BAM file was indexed using Samtools index. Postmortem damage patterns were then quantified using mapDamage2 v2.2.3 (Jónsson et al. 2013) with default parameters. The flag —single‐stranded was added for ssLibs. Along with damage plots, reads for each species mapped were extracted and median GC content (%) and fragment length (bp) were calculated and plotted.

### 2.12 Duplication Analysis

To find the percentage of unique reads in comparison to duplicated reads in each sample, the copy number of each unique read in a sample was determined. The count of occurrences per read was then grouped into duplication categories in R (unique/no duplication, duplicated 2×, duplicated 3–5×, duplicated 6–10×, duplicated 10+ times) and visualized with a bar plot.

### 2.13 Additional Plotting Aspects and Statistical Annotations

Plots were generated in R v4.3.2 (R Core Team 2023) using ggplot2 v.3.5.1 (Wickham 2016) and arranged in grids using the ggarange() function from ggpubr (Kassambara 2025) unless otherwise stated. Colour scales were obtained from the package viridis (https://github.com/sjmgarnier/viridis/). The map figure was generated using ggplot2 with geographic data from the maps package (Becker et al. 2026). Scripts to generate all main and supplemental figures can be found in the GitHub repository: https://github.com/keriburge/library_ds_vs_ss. Inkscape v1.1 was used to assemble both main and supplemental figures. All statistical annotations presented on the plots are provided in Table S2A–C.

## 3 Results

### 3.1 Initial Sequencing

**FIGURE 2:**
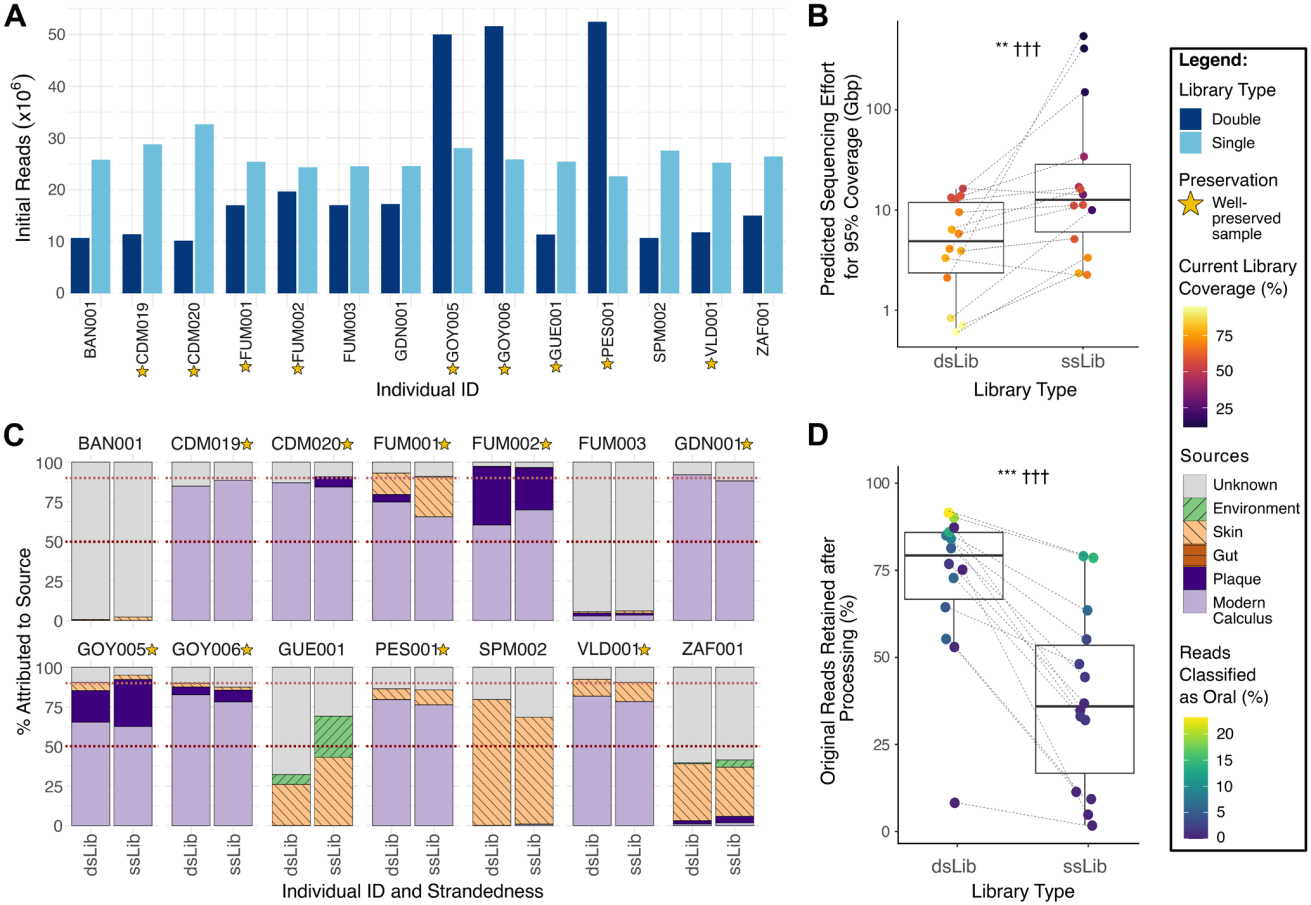
Sequencing depth, dataset complexity, contamination profiles and read retention across library preparation protocols. (A) Bar plot of the raw read count of each library before processing. (B) Predicted sequencing effort required to achieve 95% metagenomic coverage, estimated using Nonpareil. Points represent individual libraries and are coloured according to their initial estimated metagenomic coverage. (C) SourceTracker results for the NPre dataset taxonomically profiled with Kraken2. The red hashed line indicates 50% while the pink hashed line indicates 90%. Gold stars next to the sample name indicate a well‐preserved sample (> 50% oral (plaque/calculus) contribution in the SourceTracker plot and further confirmed with cumulative percent decay curves analysis). Wilcoxon signed‐rank tests were used for paired comparisons. (D) Percentage of reads retained from the NPre dataset after processing via *nf‐core/eager*. Points are coloured by the percentage of subsampled reads identified as oral. Libraries from the same individual are connected by a dotted grey line. Significance code: **p < 0.01, ***p < 0.001. Effect size (rank‐biserial r): ††† large (≥ 0.50).

Following sequencing, substantial variation in sequencing depth was observed across samples (Figure 2A; Table S3A). Median sequencing depth for dsLibs was 16,016,533 (SD = 16,271,615) read pairs and 25,618,848 (SD = 2,440,517) read pairs for ssLibs, with several dsLibs exhibiting markedly higher read counts than the rest of the dataset. In particular, GOY005, GOY006 and PES001 dsLibs each exceeded 50 million read pairs, representing clear outliers in comparison to other dsLibs. To reduce the influence of sequencing depth on downstream comparisons between library preparation protocols, two independent normalization strategies were applied: (1) subsampling to 10 million read pairs prior to read processing (NPre dataset); and (2) subsampling to 5 million reads post read processing (using the NN dataset as input) but prior to taxonomic identification (NPost dataset) (Figure 1B).

### 3.2 Library Complexity

Nonpareil (Rodriguez‐R et al. 2018; Rodriguez‐R and Konstantinidis 2014) was used to estimate metagenomic coverage and sequence complexity in the NPre dataset. Although metagenomic coverage was generally similar between dsLibs and ssLibs, dsLibs consistently exhibited higher estimated average coverage than ssLibs (Figure 2B; Figures S1 and S2; Table S3B), indicating that sequencing depth approached saturation more rapidly for processed and quality‐filtered dsLibs, whereas processed and quality‐filtered ssLibs generally remained at lower estimated coverage. This suggests that the dsLibs have more sequence redundancy.

### 3.3 Contamination Profiles

We estimated sample preservation using two approaches: cumulative percent decay (cuperdec) curves (Fellows Yates, Velsko, et al. 2021) and Bayesian source tracking with SourceTracker (Knights et al. 2011). Cuperdec profiles showed no consistent differences between library preparation protocols (Figures S3–S5). SourceTracker oral source proportions, used here as a proxy for sample preservation, did not differ significantly between library preparation protocols (*p* = 0.715; effect size = 0.11), indicating no systematic difference in overall oral preservation based on library protocol (Figure 2C; Figures S6 and S7; Table S3C). Of the fourteen starting samples, five (BAN001, FUM003, GUE001, SPM002 and ZAF001) exhibited profiles indicative of poor preservation with both library approaches in each dataset. All samples were included in downstream analysis unless otherwise stated to understand how library preparation protocol affects well‐ and poorly‐preserved samples.

### 3.4 Effects of Library Preparation Protocol on Read Processing Outcomes

During adapter removal, read merging, low‐quality end trimming, removing reads < 30 bp, removing sequencing and lab artefacts (including reads mapped to the human genome and lab standards left in the sample), and additional adapter removal with Cutadapt, the dsLibs from the NPre dataset exhibited significantly greater read retention at each processing step compared to ssLibs (*p* = 0.0001; effect size = 0.88; Figure 2D, Figures S8–S12; Table S3D). On average, dsLibs from the NPre dataset retained 72.1% of reads after processing, while ssLibs retained only 37.9%.

**FIGURE 3:**
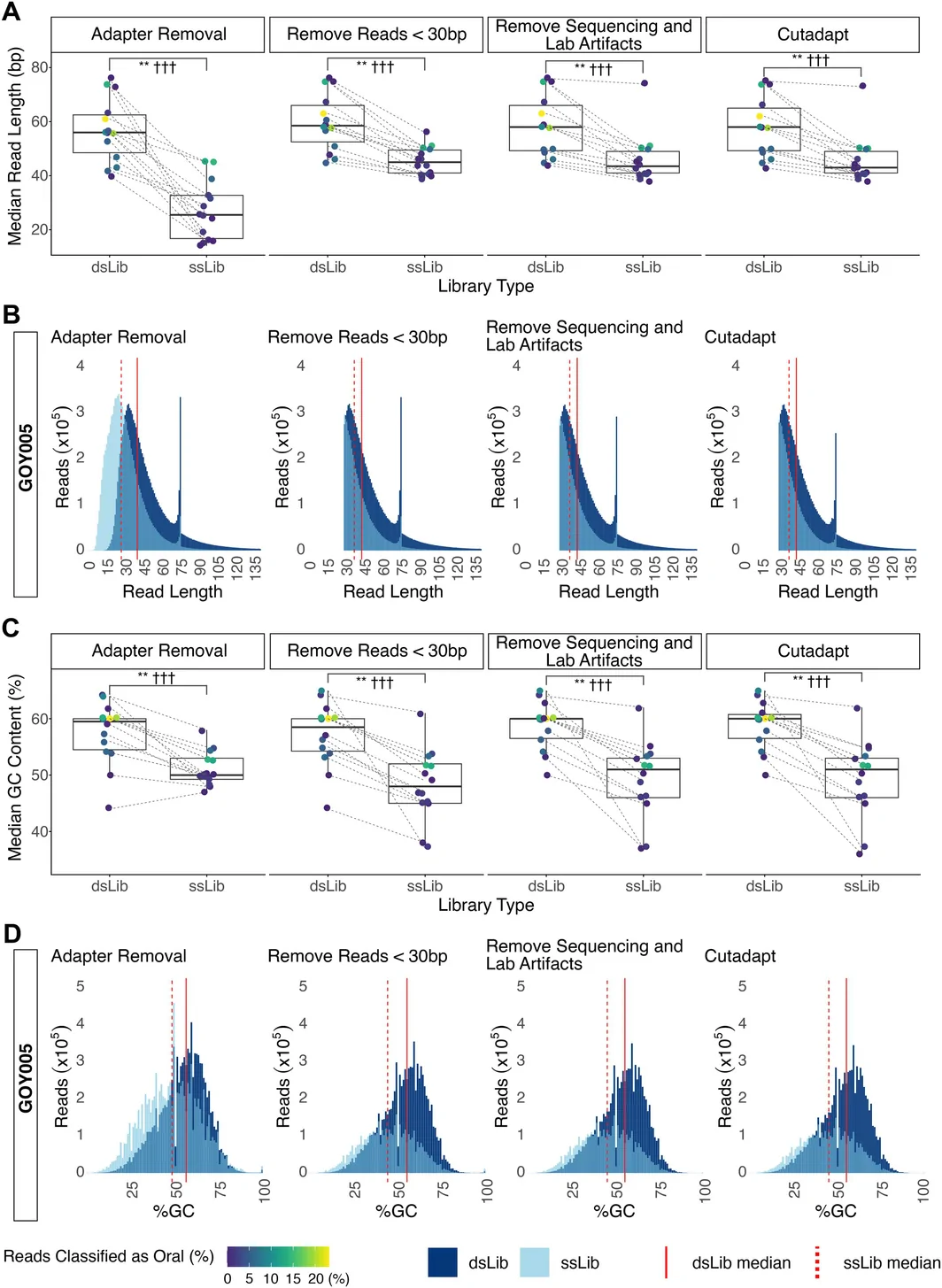
Effect of library type on read processing after subsampling to 10 million reads (NPre). (A) Median read length (bp) of dsLibs and ssLibs from the NPre dataset after each *nf‐core/eager* processing step: (1) adapter removal, read merging and low quality end trimming, (2) removing reads < 30 bp, (3) removing sequencing and lab artefacts (including reads mapped to the human genome and lab standards left in the sample), and (4) additional adapter removal with Cutadapt. Points are coloured by the percentage of subsampled reads later identified as oral. Libraries from the same individual are connected by a dotted grey line. (B) Read length distributions of NPre GOY005 libraries after each main processing step. The solid red line indicates the dsLib median while the dashed line indicates the ssLib median. Strong peaks at 75 bp are likely an artefact resulting from unmerged reads derived from fragments > 140 bp in length. (C) Median GC content (%) of NPre dsLibs and ssLibs after each main processing step. (D) GC content distributions of NPre GOY005 libraries after each main processing step. Wilcoxon signed‐rank tests were used for paired comparisons. Significance codes: **p < 0.01. Effect sizes (rank‐biserial r): ††† large (≥ 0.50).

Consistent with these differences in read retention, dsLibs also preserved significantly longer fragments following processing (median = 58 bp, SD = 11.20) than ssLibs (median = 43 bp, SD = 8.86) (*p* = 0.001; effect size = 0.88; Figure 3A; Figure S13B; Table S4A). In addition, dsLibs exhibited higher GC content after processing (median = 60%, SD = 4.28) compared to ssLibs (median = 51%, SD = 7.00) (*p* = 0.002; effect size = 0.88; Figure 3C; Figure S13C; Table S4B). We visualized read length and GC distributions throughout each processing step (Figures S8–S12), with Neanderthal GOY005 shown as an example (Figure 3B,D; Table S4C,D). The prominent 75 bp peak observed in both dsLibs and ssLibs is likely attributable to unmerged read pairs originating from fragments greater than 140 bp that could not be collapsed by AdapterRemoval, although some genuine 75 bp fragments may also contribute. Excluding 75 bp reads from median fragment length calculations had negligible effects on the reported medians (Table S4E).

The majority of read loss in ssLibs occurred during the removal of reads shorter than 30 bp. At this step, dsLibs lost only 10.4% (SD = 5.60) of reads, whereas ssLibs lost 56.8% (SD = 23.60) (*p* = 0.0001; effect size = 0.88; Figure S13A; Table S3D), indicating that fragment length filtering disproportionately impacts ssLib datasets. Analysis of the AdapterRemoval output demonstrated that adapter‐dimer‐derived reads (0 bp inserts) represented only a small fraction of merged reads (0.1%–3%), while the majority of reads < 30 bp retained authentic DNA inserts (6–29 bp). Therefore, the disproportionate loss of ssLib reads following length filtering reflects the removal of genuine ultrashort molecules recovered by the ssLib protocol rather than elevated adapter‐dimer formation (Table S4F).

### 3.5 Oral Microbiome–Focused Diversity and Compositional Analyses

Because the substantial loss of ssLib reads during NPre data processing resulted in highly uneven read counts available for taxonomic classification, we used the normalized NPost dataset to test for library‐specific differences in taxonomic composition. In the NPost dataset, libraries were subsampled to 5 million reads prior to taxonomic classification with Kraken2 (Table S5A). We next filtered the classified Kraken2 reads from the NPost dataset to include only reads assigned to oral taxa (Table S6A). Equivalent analyses performed for all classified reads prior to oral filtering are presented in Results S3.1 (Figures S14–S22; Table S5). These analyses show the same overall patterns observed for the oral‐focused analyses presented below.

**FIGURE 4:**
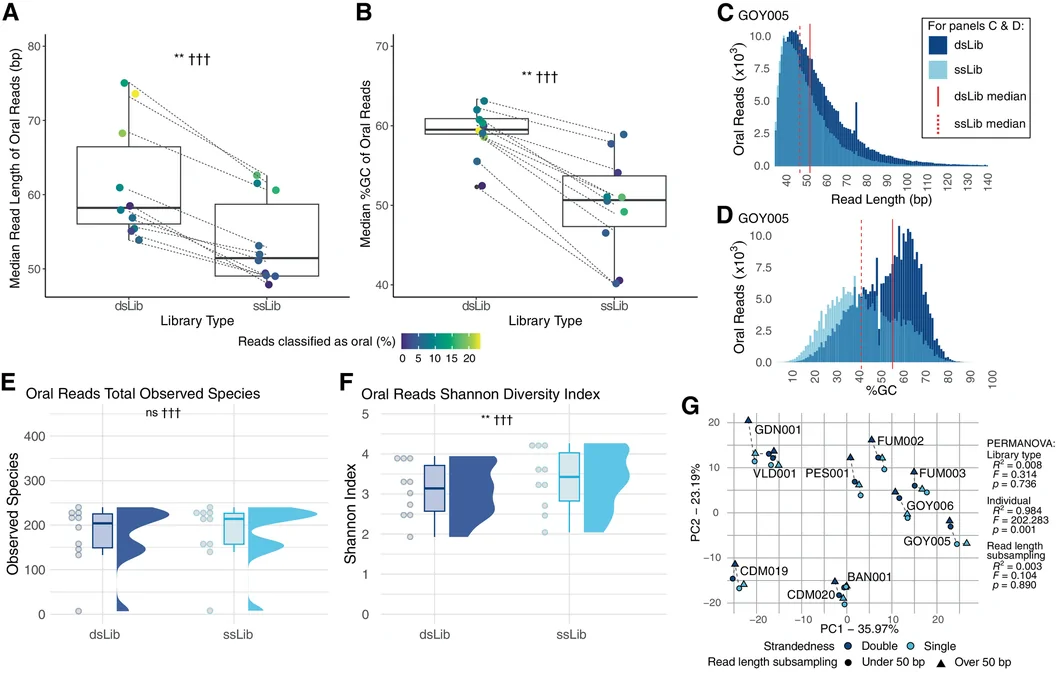
Oral‐species‐specific read characteristics and taxonomic diversity of samples profiled with Kraken2 after subsampling to 5 million reads (NPost). (A) Median read length (bp) of oral reads from the NPost dataset classified by Kraken2. Points are coloured by the percentage of the subsampled reads that were classified as oral species. (B) Median GC content (%) of reads assigned to oral taxa in the NPost dataset. (C) Read length distribution of oral GOY005 NPost reads. The solid red line indicates the dsLib median while the dashed line indicates the ssLib median. (D) GC content (%) distribution of classified GOY005 taxonomic profiling normalized oral reads. (E, F) Count of total observed oral species (E) and Shannon diversity of oral reads (F) in NPost libraries. (G) Principal components analysis (PCA) of well‐preserved NPost oral profiles (excludes BAN001 and FUM003). Points are coloured by strandedness, and the shape indicates read length subsampling (< 50 bp and ≥ 50 bp). Libraries from the same individual are connected by a hashed grey line. (A, B, G). Wilcoxon signed‐rank tests were used for paired comparisons. Significance codes: ns, *p* ≥ 0.05; **p < 0.01. Effect sizes (rank‐biserial r): ††† large (≥ 0.50).

On average, a greater proportion of oral taxa‐derived reads were recovered from dsLibs (9.78%, SD = 7.34 [67.1% of classified reads, SD = 33.60]) compared to ssLibs (6.75%, SD = 5.69 [67.3% of classified reads, SD = 33.70]) (*p* = 0.004; effect size = 0.85) out of the initial 5 million reads going into Kraken2 (Table S6B). Oral reads from dsLibs were significantly longer (median = 57.5 bp, SD = 8.09) than those from ssLibs (median = 50.5 bp, SD = 5.95) (*p* = 0.006; effect size = 0.89; Figure 4A; Table S6B). In addition, oral dsLib reads exhibited higher GC content (median = 60.5%, SD = 2.90) than oral ssLib reads (median = 52.5%, SD = 5.80) (*p* = 0.006; effect size = 0.90; Figure 4B; Table S6B), indicating that the GC‐content bias is not driven by environmental contamination. Neanderthal GOY005 is shown as an example (Figure 4C,D; Table S6C,D). The same trends were also seen in the NN and NPre datasets (Figures S23–S25).

Alpha diversity of oral‐associated taxa was subsequently assessed across normalized samples. No significant difference was observed in the total number of oral species recovered between library preparation protocols (*p* = 0.152; effect size = 0.61; Figure 4E; Table S6E), with dsLibs and ssLibs recovering an average of 177 oral species (SD = 70.50) and 181 (SD = 70.60) oral species, respectively. These trends were also observed for both the NN and NPre datasets (Figure S18A,C). In contrast, Shannon diversity was significantly higher in NPost oral ssLibs (mean = 3.35, SD = 0.76) compared to oral dsLibs (mean = 3.09, SD = 0.69) (*p* = 0.004; effect size = 0.85; Figure 4F; Table S6E), indicating a slightly more even distribution of oral species abundances in ssLibs, but the difference was minimal, suggesting that library protocol does not introduce a strong bias. No significant differences were observed in the NN and NPre datasets (Figure S18B,D).

Beta diversity differences in community composition were evaluated using a PCA of oral species‐level taxonomic profiles. Samples once again clustered significantly by individual rather than by library preparation protocol regardless of normalization (Figures S19C,D–S21C,D). As with total classified reads, preservation status did not significantly structure oral community composition.

### 3.6 Differences in Taxonomic Identification Based on Read Length

To further explore the influence of fragment length on taxonomic classification, we conducted parallel within‐library analyses comparing the < 50 and ≥ 50 bp oral read fractions for both the NPre and NPost datasets. Equivalent analyses performed for all classified reads prior to oral filtering are presented in Results S3.2 (Figures S26–S27).

For ssLibs, read length stratification did not result in significant differences in alpha diversity, regardless of normalization (Figure S26A,C). For dsLibs, in the NPre dataset, dsLibs reads ≥ 50 bp did recover significantly more oral species (average: 155, SD = 78.90) than reads < 50 bp (average: 132, SD = 83.30) (*p* = 0.006, effect size = 0.88) (Figure S26A). A similar trend was observed for the NPost dataset, although it was not significant (dsLibs: *p* = 0.561, effect size = 0.32) (Figure S26C). These trends observed in the dsLibs seem to be driven by the higher proportion and classification rate of reads ≥ 50 bp (NPre median: 4,826,502 total reads, 13.81% classified, SD = 10.93; NPost median: 3,188,216 total reads, 14.75% classified, SD = 11.16) compared to reads < 50 bp (NPre median: 3,198,294 total reads, 5.95% classified, SD = 4.19; NPost median: 1,811,784 total reads, 7.00% classified, SD = 3.91). Differences in the proportion and classification of reads ≥ 50 bp (NPre median: 1,196,979 total reads, 10.00% classified, SD = 9.57; NPost median: 1,695,666 total reads, 11.15% classified, SD = 9.59) and < 50 bp (NPre median: 2,472,280 total reads, 3.88% classified, SD = 3.56; NPost median: 3,304,334 total reads, 4.39% classified, SD = 3.61) were smaller in ssLibs.

Shannon diversity of the oral species profiles from the NPost dataset (Figure S26D) did not significantly differ between read length fractions. No significant differences were observed for dsLibs (*p* = 0.590; effect size = 0.24) or ssLibs (*p* = 0.840; effect size = 0.11). The same trends for Shannon diversity were observed in the NPre dataset (Figure S26B).

When plotting the length‐stratified datasets in a PCA, we saw once again that interindividual separation on the PCA is greater than separation by library protocol or read length stratification for both the NPre (Figure S27) and NPost datasets (Figure 4G; Figure S28; Table S6F). Along PC2, dsLibs ≥ 50 bp skewed toward more positive scores compared to the other datasets, indicating slight differences that differentiate the dsLib ≥ 50 bp dataset from the others.

Together, these results indicate that, within the read length ranges examined, longer fragments have a slight advantage for oral taxonomic recovery relative to shorter fragments, but this is only observed for dsLibs. Overall, although dsLibs yield a greater proportion of longer reads, differences in read length alone do not appear to drive major differences in taxonomic profiles between library preparation protocols.

### 3.7 Oral Species‐Level Drivers of Compositional Differences Between Library Protocols

Although individual and preservation status explained most of the variance in taxonomic composition, the partial separation of libraries by protocol along PC1 suggested that systematic technical biases may influence species recovery. To further investigate this pattern, we examined the taxa contributing most strongly to each principal component and evaluated whether protocol‐specific differences in read length and GC content were associated with differential recovery of individual species within the NPost dataset. To minimize the effects of preservation, this analysis was restricted to well‐preserved samples only.

**FIGURE 5:**
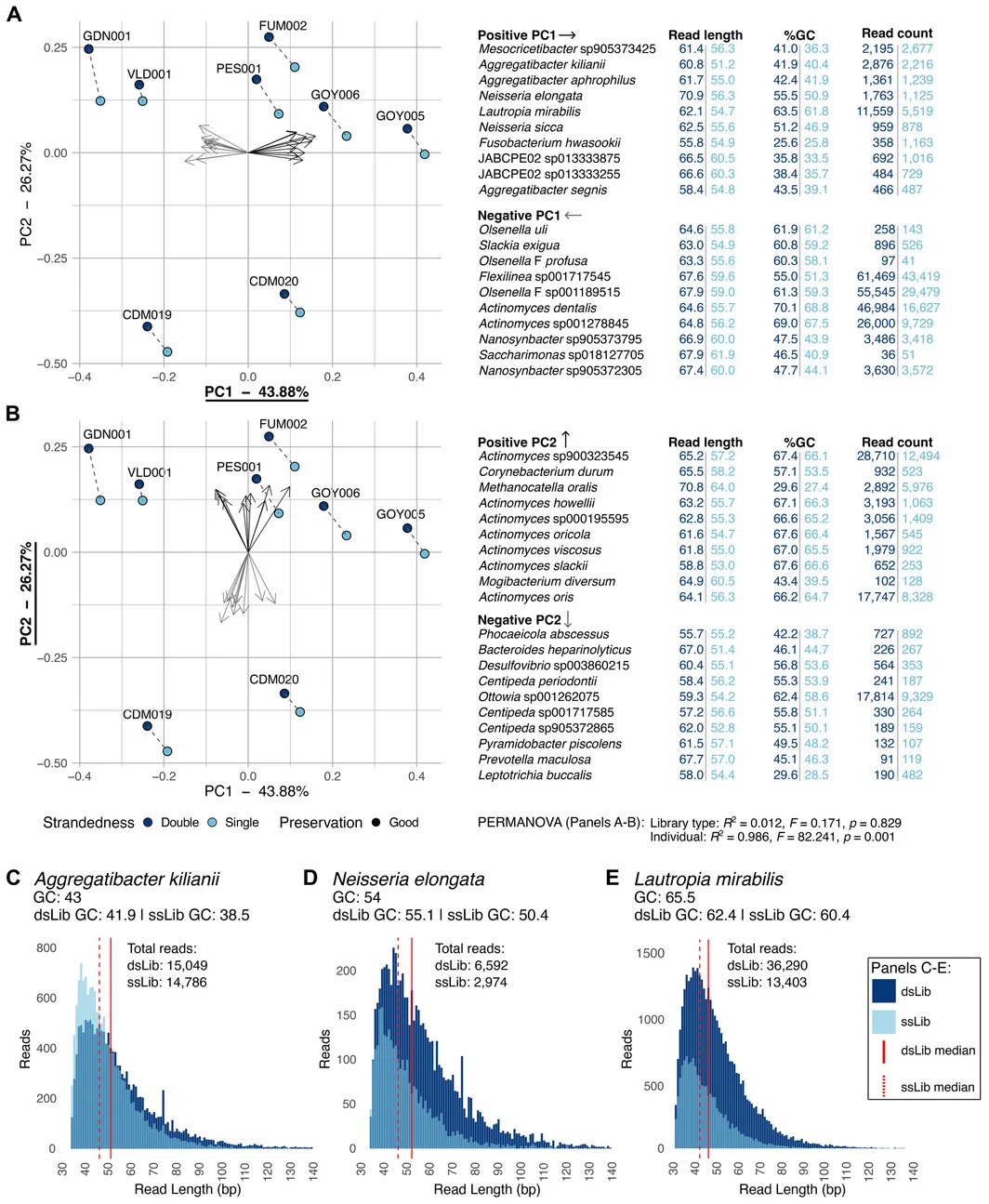
Oral‐species–specific drivers of PCA structure and library‐dependent recovery of taxa. (A) Principal component analysis (PCA) of samples based on relative abundances of oral species classified by Kraken2 in the NPost dataset. Arrows indicate species loadings, with the direction and magnitude reflecting their contribution to PC1. Species with the strongest positive and negative loadings on PC1 are listed, alongside mean read length, GC content and read counts for each library type. (B) PCA highlighting species driving separation along PC2, with associated species loadings and summary statistics shown. Read length (bp), GC (%) and read count were calculated by averaging across the samples in which the species were present. Not all species were present across all samples. Libraries from the same individual are connected by a hashed grey line (A, B). Only well‐preserved samples are shown (A, B). (C–E) Read length distributions for representative oral species in GOY005 NPost libraries spanning low (C), intermediate (D) and high (E) genomic GC content. Reads with Kraken2 species identifications for the species of interest were extracted.

In this subset, samples clustered most strongly by individual (PERMANOVA: *R* ^2^ = 0.986, *F* = 82.241, *p* = 0.001) and library preparation protocol explained little overall variance (*R* ^2^ = 0.012, *F* = 0.171, *p* = 0.829; Figure 5A,B; Table S7A). Despite this, consistent shifts along both PC1 and PC2 were observed between library types. Along PC1, ssLibs tended toward more positive scores while dsLibs were shifted toward more negative scores. Along PC2, dsLibs exhibited more positive scores and ssLibs were more negative.

Examination of the species enriched in each loading showed no clear correlation in the GC content of the taxa. For example, positive PC1 was not enriched solely in low‐GC‐content taxa (Figure 5A; Table S7B). Instead, persisting trends were seen in which dsLibs recover longer fragments of the same species compared to ssLibs, and further have higher GC content of that particular species. When considering the average read count per species (Figures S29–S34), we saw that ssLibs only outperform dsLibs in read recovery when it comes to low‐GC‐content species such as *Methanocatella oralis* (Figure S29).

To further illustrate these patterns, read length distributions of Kraken2 assigned species reads were examined across representative oral taxa spanning low‐, intermediate‐ and high‐GC content (Figure 5C–E; Table S7D–F). Double‐stranded libraries consistently recovered greater numbers of longer reads for intermediate‐GC species such as *Neisseria elongata* (Figure 5D; Figure S30; Table S7E) and *Flexilinea* sp001717545 (*Anaerolineaceae* bacterium oral taxon 439) (Figure S31), and for high‐GC species such as *Lautropia mirabilis* (Figure 5E; Figure S32; Table S7F) and *Actinomyces dentalis* (Figure S33). In contrast, ssLibs showed relatively improved recovery of low‐GC taxa such as *Aggregatibacter kilianii* (Figure 5C; Figure S34; Table S7D) and *M. oralis* (Figure S29), primarily through an increased abundance of shorter fragments. All species analysed show characteristic patterns of aDNA damage (Figures S29–S34). Together, these results indicate that subtle but consistent protocol‐specific biases in fragment length and GC recovery may influence species‐level representation and contribute to the observed structure in ordination space.

We next plotted median GC content and fragment length of reads mapped to the species previously mentioned and compared these medians to the median GC content of the reference genome used (Figure S35; Table S7C). We found that while dsLibs consistently have higher GC content compared to their ssLib counterpart across all species, we additionally see overarching trends in the ancient samples regardless of library type. For low‐GC‐content species like *M. oralis* , we see ancient samples have a higher median GC content compared to the reference genome, while with intermediate‐GC‐content species (such as *N. elongata* and *Anaerolineaceae* bacterium oral taxon 439), ancient samples are more similar to the median. With high‐GC‐content species like *L. mirabilis* and *A. dentalis* , ancient samples cluster lower than the expected median based on the reference, indicating that additional research is needed to better understand the sequencing biases affecting ancient metagenomic samples in general, regardless of library protocol.

### 3.8 Clonality

Because dsLibs yielded a higher number of oral reads on average compared to ssLibs, we next evaluated whether differences in read duplication (clonality) could account for this pattern. If a substantial fraction of dsLib oral reads represents duplicates, higher read counts may not translate into a broader representation of the microbial community. To assess clonality, we calculated the proportion of unique reads assigned by Kraken2 as oral taxa. An equivalent analysis performed for all classified reads prior to oral filtering is presented in Results S3.3 (Figure S36).

On average, across the NPost dataset, dsLibs consisted of 86.9% (SD = 22.40) unique oral reads on average (mean = 437,302, SD = 381,337), whereas ssLibs consisted of 95.4% (SD = 4.64) unique oral reads (mean = 329,840, SD = 286,148) (*p* = 0.027; effect size = 0.69; Figure S37C). This was additionally seen in the NN and NPre datasets (Figure S37A,B).

Although dsLibs therefore showed a higher degree of read duplication overall, clonality varied substantially across samples, indicating that this effect is sample‐dependent. For example, sample GDN001 represented a clear outlier in both library preparation protocols, exhibiting elevated levels of duplication relative to other samples (Figure S37). Further, when analysing the degree of duplication within dsLib oral reads from the NPost dataset, out of the average 13.1% duplicated oral reads (mean = 54,424, SD = 118,830), most reads (mean 90.3%, SD = 19.90) were only a single duplication, indicating that the degree of clonality within dsLibs is not severe despite the increased percentage of duplicated reads compared to ssLibs (Figure S37C).

## 4 Discussion

### 4.1 Protocol‐Dependent Trade‐Offs in Read Recovery and Taxonomic Profiling

We compared an optimized implementation of the Meyer and Kircher (2010) dsLib preparation protocol (Aron, Neumann, et al. 2020; Stahl et al. 2023) and the Gansauge et al. (2017, 2020) ssLib (Nagel et al. 2025, 2026) preparation protocol for ancient oral microbiome analysis using dental calculus samples from twelve Neanderthals (aged ~43,000–102,000 BP) and two Chalcolithic humans (aged ~4200 BP) spanning a range of preservation states. Across analyses, neither protocol was consistently superior: dsLibs increased read recovery, the number of total and oral species classified and the fragment length, while ssLibs retained a higher proportion of unique reads. GC content also consistently differed between the library preparation protocols, with dsLibs having higher and ssLibs having lower GC values. However, despite read‐level differences, both approaches produced broadly comparable taxonomic profiles, even for samples 40,000 to 100,000 years old. Overall, we find that individual differences and preservation status drive the majority of variation between library samples, which supports previous studies showing that one library protocol does not consistently outperform the other across all metrics (Wright et al. 2025).

Given the stark read‐level differences between protocols, it is necessary to assess how increased sequencing yield in dsLibs affects redundancy and overall dataset complexity. Increased read recovery and coverage can result in higher redundancy in metagenomic datasets (Rodriguez‐R et al. 2018; Rodriguez‐R and Konstantinidis 2014). Consistent with this, dsLibs showed higher estimated coverage in Nonpareil plots but also greater clonality in both classified reads and reads identified as oral. As a result, the increased read recovery in dsLibs compared to ssLibs across processing and taxonomic profiling may not necessarily translate into greater community‐wide information. This is supported by comparable species richness between protocols, indicating that additional dsLib reads being classified did not recover species beyond those identified in ssLibs. Furthermore, the slightly higher Shannon diversity observed in ssLibs despite comparable richness between library protocols suggests a more even distribution of reads across taxa, whereas the additional reads recovered in dsLibs appear to disproportionately increase coverage of already abundant taxa, consistent with greater redundancy. Together, these results emphasize that protocol comparisons should consider not only gross yield but also how those reads are distributed across taxa.

### 4.2 GC Content and Fragment Length as Drivers of Library‐Specific Biases

Differences in fragment length and GC content were the most consistent read‐level patterns, with dsLibs retaining longer and more GC‐enriched reads, consistent with previous comparisons of dsLibs and ssLibs (Wales et al. 2015; Wright et al. 2025). In ssLibs, read‐length stratification did not significantly affect species richness or Shannon diversity. However, in dsLibs, reads ≥ 50 bp recovered slightly more species but showed no difference in Shannon diversity compared to the < 50 bp dataset. This could perhaps indicate higher taxonomic assignment for longer reads with more specificity, although dsLibs retaining a greater proportion of longer reads did not significantly affect overall community composition compared to ssLibs.

The loss of short AT‐rich fragments in ancient microbial DNA has been previously reported (Fagernäs et al. 2020; Mann et al. 2018). This effect is likely more pronounced in dsLibs because AT‐rich fragments are less thermally stable compared to GC‐rich fragments (Panjkovich et al. 2005). With these AT‐rich fragments being more prone to strand separation, fragmentation and damage, if one strand is lost or heavily nicked, then that molecule cannot be incorporated into the dsLib since intact double‐stranded molecules are required (Briggs et al. 2007; Kircher et al. 2012; Meyer and Kircher 2010). In contrast, ssLibs begin by heat denaturing (Gansauge et al. 2017, 2020; Gansauge and Meyer 2013, 2019; Meyer et al. 2012) and individual damaged strands (which may be more AT‐rich) are directly incorporated into the library.

Species‐level analyses showed that this pattern is not simply explained by preferential recovery of high‐GC taxa in dsLibs. Instead, for the same species, dsLibs typically recovered longer and more GC‐enriched fragments. Differences in retention were seen when comparing species by their GC content (low, intermediate and high). For intermediate‐ and high‐GC species, dsLibs recovered a greater number of longer reads, whereas ssLibs showed relatively improved recovery for low‐GC species via an enrichment of shorter fragments. Previous studies have reported GC‐content biases associated with ancient DNA library preparation, although the direction and magnitude of these biases depend on the protocols being compared (Kapp et al. 2021; Wales et al. 2015). However, comparisons to reference genomes further revealed a trend regardless of library protocol, indicating that ssLibs may not always be a better reflection of original GC composition. With low‐GC‐content species, ancient samples have a higher median GC content in mapped reads compared to the reference genome, while with intermediate‐GC‐content species, ancient reads are more similar in GC content to the reference genome median, and with high‐GC‐content species, ancient samples have lower than the expected median GC content compared to the reference, indicating that not all genomes exhibit a trend toward GC enrichment in dsLibs. Additional species‐level research is needed to elucidate how sequencing biases affect specific species retention in ancient metagenomic samples, regardless of library protocol.

### 4.3 Biological Signal Outweighs Library Preparation Effects

Across all ordinations, the strongest clustering signal was the individual. This indicated that the library preparation protocol does not override the underlying biological signal in ancient dental calculus microbiomes. Instead, protocol effects appear as secondary shifts, where differences in the read‐level properties described previously introduce minor variation between library types of the same individual. Preservation was additionally an important axis of variation, particularly before restricting to only oral taxa. After filtering to oral taxa, the influence of preservation on beta diversity was reduced, suggesting that much of the preservation‐associated separation is driven by non‐oral taxa that are removed through oral species filtering. Therefore, while library protocol choice affects read retention and some aspects of species representation, the broad structure of oral community composition remains determined by biological and taphonomic factors.

### 4.4 Implications for Ancient Oral Microbiome Study Design

For future ancient oral microbiome studies, library preparation protocol choice should be informed by the study objectives rather than by assuming one protocol outperforms the other. Double‐stranded libraries are advantageous when the goal is to maximize read length and the number of identifiable oral reads. Metagenomic assembly in particular benefits from longer reads and higher coverage. Current reference databases, such as NCBI and GTDB are incomplete and lack an exhaustive representation of the genomic diversity found in oral taxa. Recent studies incorporating metagenomic assembled genomes (MAGs) are substantially expanding our understanding of the known diversity of oral microbiomes through time (Galtier et al. 2026; Velsko, Hübner, et al. 2025). However, sufficient read length is required to recover high completeness and low contamination MAGs (Klapper et al. 2023; Wibowo et al. 2021), which may make dsLibs more favourable for metagenomic assembly, although deeper sequencing would be needed for a systematic comparison of assembly success between the library types in this study.

In practice, however, protocol choice is a balance between data characteristics and logical considerations. While dsLibs may be advantageous for applications requiring longer reads and higher coverage, ongoing refinements to both dsLib (Carøe et al. 2018) and ssLib (Kapp et al. 2021) protocols are reducing differences in yield and cost. Importantly, despite these differences, results indicate that dsLibs and ssLibs can be directly compared within a single study without introducing major library protocol‐driven biases in community composition. More broadly, these findings suggest that systematic evaluations of other methodological variables in ancient microbial metagenomics would be valuable. As the dsLib and ssLib protocols use different DNA‐extract input volumes, differences in library complexity, duplication rates and taxonomic recovery may reflect not only the library preparation method itself but also the unequal number of DNA molecules entering library construction. Future studies comparing these protocols would therefore benefit from using identical input volumes. Similar comparative studies examining DNA extraction methods, polymerases, uracil‐DNA glycosylase (UDG) treatment strategies and other laboratory workflows across a variety of samples would help determine whether these technical differences likewise preserve underlying community composition, ultimately improving the comparability of ancient microbiome datasets generated across laboratories.

## 5 Conclusion

In this study, we provided a systematic comparison of double‐stranded (implementation of Meyer and Kircher 2010) and single‐stranded (implementation of Gansauge et al. 2017, 2020) library preparation protocols for ancient oral microbiome analysis using dental calculus samples from Neanderthals and Chalcolithic humans spanning a range of preservation states. Despite fundamental differences in how these protocols recover aDNA, no single protocol consistently outperformed the other. Instead, dsLibs and ssLibs exhibited distinct and predictable trade‐offs: dsLibs retained a greater proportion of reads throughout processing, recovered longer and more GC‐enriched fragments and yielded more classified and oral reads while ssLibs produced a higher proportion of unique reads and retained shorter AT‐rich fragments. Importantly, however, these protocol‐dependent differences did not substantially affect overall taxonomic richness or obscure dominant biological or taphonomic signals.

This study directly addresses a central challenge in microbial paleogenomics, where methods have previously prioritized the recovery of ultrashort DNA fragments. The increased recovery of short, damaged DNA in ssLibs does not necessarily improve taxonomic resolution for microbial communities. Further, longer fragments thought to be preferential for taxonomic assignment did not differentiate the community composition of a dsLib from its ssLib counterpart. Accordingly, protocol choice should be guided by study goals and sample context. Double‐stranded libraries may be more advantageous for applications requiring longer reads and higher coverage (e.g., metagenomic assembly), and ssLibs are beneficial for highly degraded samples where short fragment recovery is critical. More broadly, our findings demonstrate that dsLibs and ssLibs can be directly compared within a single study without introducing major biases in community composition. Nevertheless, as the field of paleogenomics expands to include increasingly complex microbial communities, continued methodological refinement and systematic cross‐sample comparisons will be essential for robustly reconstructing ancient microbial ecosystems.

## Author Contributions

C.W. and I.M.V. conceived and supervised the study. D.C.S.‐G., M.H.U. and J.L.M. contributed materials and resources. K.B. performed data analysis. K.B. wrote the manuscript with editing from I.M.V. and C.W., and with input and contributions from all coauthors.

## Funding

This research was funded by the American School of Prehistoric Research, Carolyn Weinberg and the Radcliffe Institute, the Werner Siemens Foundation (Paleobiotechnology to C.W.), the Deutsche Forschungsgemeinschaft (German Research Foundation) under Germany's Excellence Strategy EXC 2051 (Project No. 390713860, “Balance of the Microverse” to C.W.) and the National Science Foundation (GRFP to K.B).

## Ethics Statement

New genetic sequencing data was generated from twelve previously published dental calculus samples and two newly reported dental calculus samples with permission from the original archaeologists and museum curators. All other archaeological genetic sequence data was obtained through the AncientMetagenomeDir database and downloaded from public data repositories. Modern comparative genetic sequence data was additionally downloaded from public data repositories.

## Conflicts of Interest

The authors declare no conflicts of interest.

## Supporting information


**Figure S1:** Estimated metagenomic dataset coverage for samples normalized to 10 million reads going into *nf‐core/eager* (NPre dataset). Nonpareil curves for each sample showing the estimated coverage in each metagenomic dataset. The horizontal dashed lines indicate 100 (grey) and 95% (red) coverage. The empty circles indicate the size and estimated average coverage of the datasets, and the lines after that point are projections of the fitted model. Arrows on the *x*‐axis represent the location corresponding to exp(Nd), which is a metric of sequence diversity based on the projection of the Nonpareil curve. Curves with corresponding arrows that are more to the right indicate a higher sequence diversity. Each plot shows both the dsLib (dark blue) and ssLib (light blue) of each sample.
**Figure S2:** Estimated metagenomic dataset coverage for samples not normalized going into *nf‐core/eager* (NN dataset). Nonpareil curves for each sample showing the estimated coverage in each metagenomic dataset. The horizontal dashed lines indicate 100 (grey) and 95% (red) coverage. The empty circles indicate the size and estimated average coverage of the datasets, and the lines after that point are projections of the fitted model. Arrows on the *x*‐axis represent the location corresponding to exp(Nd), which is a metric of sequence diversity based on the projection of the Nonpareil curve. Curves with corresponding arrows that are more to the right indicate a higher sequence diversity. Each plot shows both the dsLib (dark blue) and ssLib (light blue) of each sample.
**Figure S3:** Sample preservation status with no normalization (NN dataset). (A) Cumulative percent decay (cuperdec) plots showing the fraction of oral taxa relative to all taxa, as ordered by abundance rank for the sample set that was not normalized going into both *nf‐core/eager* and Kraken2 (NN dataset). The *x*‐axis is restricted to 250 positions for visualization purposes. Modern reference datasets (plaque and environmental samples) are shown in the bottom row for comparison. Each plot shows the cuperdec curve for both the dsLib (darker line) and ssLib (lighter, more transparent line) of each sample. A 50% threshold with an adaptive burin determines a passing sample. (B) Summary of preservation status by individual and library strandedness. Each row represents an individual, with bars indicating whether dsLibs and/or ssLibs were classified as well (dark grey) or poorly preserved (light grey) as determined by the cuperdec curves (A).
**Figure S4:** Normalizing 10 million reads into *nf‐core/eager* (NPre) sample preservation status. (A, C, E) Cumulative percent decay (cuperdec) plots showing the fraction of oral taxa relative to all taxa, as ordered by abundance rank for the NPre dataset that was normalized 10 million reads prior to processing with *nf‐core/eager* (A) as well as further subsampled datasets separating reads ≥ 50 bp (C) and reads < 50 bp (E). The *x*‐axis is restricted to 250 positions for visualization purposes. Modern reference datasets (plaque and environmental samples) are shown in the bottom row for comparison. Each plot shows the cuperdec curve for both the dsLib (darker line) and ssLib (lighter, more transparent line) of each sample. A 50% threshold with an adaptive burin determines a passing sample. (B, D, F) Summary of preservation status by individual and library strandedness. Each row represents an individual, with bars indicating whether dsLibs and/or ssLibs were classified as well (dark grey) or poorly preserved (light grey) as determined by the cuperdec curves. Summary plots are included for all reads (B), reads ≥ 50 bp (D) and reads < 50 bp (F).
**Figure S5:** Normalizing 5 million reads into Kraken2 (NPost) sample preservation status. (A, C, E) Cumulative percent decay (cuperdec) plots showing the fraction of oral taxa relative to all taxa, as ordered by abundance rank for the NPost dataset that was normalized 5 million reads prior to taxonomic profiling with Kraken2 (A) as well as further subsampled datasets separating reads ≥ 50 bp (C) and reads < 50 bp (E). The *x*‐axis is restricted to 250 positions for visualization purposes. Modern reference datasets (plaque and environmental samples) are shown in the bottom row for comparison. Each plot shows the cuperdec curve for both the dsLib (darker line) and ssLib (lighter, more transparent line) of each sample. A 50% threshold with an adaptive burin determines a passing sample. (B, D, F) Summary of preservation status by individual and library strandedness. Each row represents an individual, with bars indicating whether dsLibs and/or ssLibs were classified as well (dark grey) or poorly preserved (light grey) as determined by the cuperdec curves. Summary plots are included for all reads (B), reads ≥ 50 bp (D) and reads < 50 bp (F).
**Figure S6:** SourceTracker plots show source composition of each sample. Percentage bar plots showing the percentage of sources (environment, skin, gut, modern dental plaque, modern dental calculus and unknown) attributed to each metagenomic sample for each normalization tract: no normalization (NN) (A), normalizing to 10 million reads prior to processing (NPre) (B) and normalizing to 5 million reads prior to taxonomic profiling (NPost) (C). The red hashed line indicates 75% while the pink hashed line indicates 90%. Stars next to the sample name indicates a well preserved sample. For the two normalization datasets (C, D), read length stratification datasets (< 50 bp versus ≥ 50 bp) are additionally shown.
**Figure S7:** Rarefied PCoAs of ancient dental calculus and source samples. Principal coordinates analysis (PCoA) for ancient dental calculus samples and source samples (environment, skin, gut, modern dental plaque and modern calculus) for each normalization dataset: no normalization (NN) (A), normalizing to 10 million reads prior to processing (NPre) (B) and normalizing to 5 million reads prior to taxonomic profiling (NPost) (C). The variance explained by each principal coordinate is written on the axes. Dark grey hashed lines connect the dsLib and ssLib from the same individual. The Kraken2 total species tables used for these analyses were filtered, removing low abundance taxa, before plotting.
**Figure S8:** CDM read length and GC content throughout processing steps. Histograms showing the distribution of read length (bp) (A, C) and GC content (%) (B, D) for NPre CDM019 (A, B) and CDM020 (C, D) throughout the main read processing steps: adapter removal and merging, removing reads < 30 bp, removing sequencing and laboratory artefacts and additional adapter removal with Cutadapt. The solid red line indicates the dsLib median while the dashed line indicates the ssLib median.
**Figure S9:** Read length and GC content throughout processing steps for poorly preserved samples BAN, GUE and ZAF. Histograms showing the distribution of read length (bp) (A, C, E) and GC content (%) (B, D, F) for NPre BAN001 (A, B), GUE001 (C, D) and ZAF001 (E, F) throughout the main read processing steps: adapter removal and merging, removing reads < 30 bp, removing sequencing and laboratory artefacts, and additional adapter removal with Cutadapt. The solid red line indicates the dsLib median while the dashed line indicates the ssLib median.
**Figure S10:** Read length and GC content throughout processing steps for poorly FUM samples. Histograms showing the distribution of read length (bp) (A, C, E) and GC content (%) (B, D, F) for NPre FUM001 (A, B), FUM002 (C, D) and FUM003 (E, F) throughout the main read processing steps: adapter removal and merging, removing reads < 30 bp, removing sequencing and laboratory artefacts and additional adapter removal with Cutadapt. The solid red line indicates the dsLib median while the dashed line indicates the ssLib median.
**Figure S11:** Read length and GC content throughout processing steps for GDN, GOY and VLD. Histograms showing the distribution of read length (bp) (A, C, E) and GC content (%) (B, D, F) for NPre GDN001 (A, B), GOY006 (C, D) and VLD001 (E, F) throughout the main read processing steps: adapter removal and merging, removing reads < 30 bp, removing sequencing and laboratory artefacts and additional adapter removal with Cutadapt. The solid red line indicates the dsLib median while the dashed line indicates the ssLib median.
**Figure S12:** PES and SPM read length and GC content throughout processing steps. Histograms showing the distribution of read length (bp) (A, C) and GC content (%) (B, D) for NPre PES001 (A, B) and SPM002 (C, D) throughout the main read processing steps: adapter removal and merging, removing reads < 30 bp, removing sequencing and laboratory artefacts and additional adapter removal with Cutadapt. The solid red line indicates the dsLib median while the dashed line indicates the ssLib median.
**Figure S13:** Read retention and processing outcomes in *nf‐core/eager* after subsampling to 10 million reads (NPre dataset). (A) Total number of reads of both dsLibs (dark blue) and ssLibs (light blue) throughout the main read processing steps (adapter removal and merging, removing reads < 30 bp, removing sequencing and lab artefacts and running Cutadpt to remove persisting adapter sequences). (B) Percent reduction in read length after read processing (as compared to the initial reads sequenced at 75 bp). (C) Percent of change in GC content after processing. Points (B, C) are coloured by the percentage of reads classified as oral and libraries from the same sample are connected by a hashed grey line. Wilcoxon signed‐rank tests were used for paired comparisons. Significance codes: **p < 0.01; ***p < 0.001. Effect sizes (rank‐biserial r): ††† large (≥ 0.50).
**Figure S14:** Sequencing read characteristics and taxonomic diversity of samples profiled with Kraken2 after subsampling to 5M reads (NPost). (A) Median read length (bp) of reads from the NPost dataset classified by Kraken2. Points are coloured by the percentage of the subsampled reads that were classified to the species level. (B) Median GC content (%) of reads classified by Kraken2. (C) Read length distribution of classified NPost GOY005 taxonomic profiling normalized libraries. The solid red line indicates the dsLib median while the dashed line indicates the ssLib median. (D) GC content (%) distribution of classified NPost GOY005 reads. (E, F) Count of total observed species (E) and Shannon diversity (F) in NPost libraries classified by Kraken2. G. Principal components analysis (PCA) of Kraken2 taxonomic profiles from the NPost dataset. Points are coloured by strandedness, and the shape indicates preservation status. Libraries from the same sample are connected by a hashed grey line (A, B, G). Wilcoxon signed‐rank tests were used for paired comparisons. Significance codes: ns, *p* ≥ 0.05; **p < 0.01. Effect sizes (rank‐biserial r): †† moderate (0.30–0.49), ††† large (≥ 0.50).
**Figure S15:** Read lengths distributions of classified reads by sample. Histograms showing the counts of reads classified by Kraken2 at each read length for the no normalization (NN) dataset (A), normalising 10 million reads prior to processing (NPre) (B), and normalising 5 million reads prior to taxonomic profiling (NPost) **(C)** divided by sample. Red solid lines indicate the dsLib median per sample while the hashed red lines indicate the ssLib median per sample.
**Figure S16:** GC content distributions of classified reads by sample. Histograms showing the counts of reads classified by Kraken2 at each %GC bin for the no normalization (NN) dataset (A), normalising 10 million reads prior to processing (NPre) (B), and normalizing 5 million reads prior to taxonomic profiling (NPost) (C) divided by sample. Red solid lines indicate the dsLib median per sample while the hashed red lines indicate the ssLib median per sample.
**Figure S17:** Median read length and %GC of classified Kraken2 reads. Boxplots showing the median read length (bp) (A, C) and %GC (B, D) of the total classified reads from each sample for the no normalization (NN) dataset (A, B) and the NPre dataset subsampling to 10 million reads prior to read processing (C, D). Grey hashed lines connect the dsLib and ssLib of the same individual. Points are coloured by the percent of processed reads (post *nf‐core/eager* and Cutadpt) classified by Kraken2. Wilcoxon signed‐rank tests were used for paired comparisons. Significance codes: **p < 0.01. Effect sizes (rank‐biserial *r*): ††† large (≥ 0.50).
**Figure S18:** Alpha diversity analyses for both total classified reads and oral reads. Raincloud plots showing both the total observed species count (A, C, E) and Shannon diversity index (B, D, F) for each normalization dataset: no normalization (NN) (A, B), normalizing to 10 million reads prior to processing (NPre) (C, D) and normalizing to 5 million reads prior to taxonomic profiling (NPost) (E, F). For each plot, the left panel shows the alpha‐diversity metric for all reads classified by Kraken2, and the right panel shows the alpha‐diversity metric for only reads assigned to oral taxa. Wilcoxon signed‐rank tests were used for paired comparisons. Significance codes: ns, *p* ≥ 0.05; *p < 0.05; **p < 0.01. Effect sizes (rank‐biserial r): † small (< 0.30), †† moderate (0.30–0.49), ††† large (≥ 0.50).
**Figure S19:** Drivers of PCA structure for dataset normalizing 10 million reads prior to processing steps. Principal component analysis (PCA) of samples based on NPre Kraken2 species tables after normalizing to 10 million reads prior to processing via *nf‐core/eager* for both total species classified (A, B) and reads attributed only to oral species (C, D). A, C. Arrows indicate species loadings, with the direction and magnitude reflecting their contribution to PC1. Species with the strongest positive and negative loadings on PC1 are listed. B, D. PCA highlighting species driving separation along PC2, with associated species loadings. Libraries from the same sample are connected by a hashed grey line. Asterisks next to species names in the total species (A, B) PCAs indicate oral associated taxa.
**Figure S20:** Drivers of PCA structure for the no normalization dataset. Principal component analysis (PCA) of samples based on NN Kraken2 species tables after not normalizing prior to both processing and taxonomic profiling for total species (A, B), oral species (C, D) and oral species of only well‐preserved samples (no BAN001, FUM003, GUE001, SPM001, or ZAF001). (A, C, E) Arrows indicate species loadings, with the direction and magnitude reflecting their contribution to PC1. Species with the strongest positive and negative loadings on PC1 are listed. (B, D, F) PCA highlighting species driving separation along PC2, with associated species loadings. Libraries from the same sample are connected by a hashed grey line. Asterisks next to species names in the total species (A, B) PCAs indicate oral associated taxa.
**Figure S21:** Drivers of PCA structure for dataset normalizing 5 million reads into Kraken2 (NPost). Principal component analysis (PCA) of samples based on NPost Kraken2 species tables after normalizing to 5 million reads for both total species (A, B) and oral species (C, D). (A, C) Arrows indicate species loadings, with the direction and magnitude reflecting their contribution to PC1. Species with the strongest positive and negative loadings on PC1 are listed. (B, D) PCA highlighting species driving separation along PC2, with associated species loadings. Libraries from the same sample are connected by a hashed grey line. Asterisks next to species names in the total species (A, B) PCAs indicate oral associated taxa.
**Figure S22:** Total species beta diversity and dispersion of well‐preserved samples from the NPost dataset (subsampled to 5M reads prior to taxonomic profiling). (A, B) Principal component analysis (PCA) of samples based on Kraken2 total species tables after filtering for only well‐preserved samples in the NPost dataset. (A) Arrows indicate species loadings, with the direction and magnitude reflecting their contribution to PC1. Species with the strongest positive and negative loadings on PC1 are listed. (B) PCA highlighting species driving separation along PC2, with associated species loadings. Libraries from the same sample are connected by a hashed grey line. (C) Principal coordinates analysis (PCoA) of Euclidean distances showing sample clustering, with ellipses representing group dispersion and crosses indicating group centroids. (D) Distances to group centroids (C) (with vegan function betadisper (Oksanen et al. 2025)) for each sample by library type, with paired samples connected by dashed lines. Wilcoxon signed‐rank tests were used for paired comparisons. Significance codes: ns, *p ≥ 0.5*. Effect size: †† moderate (0.30–0.49).
**Figure S23:** Read lengths distributions of oral reads by sample. Histograms showing the counts of oral reads at each read length for the no normalization (NN) dataset (A), normalizing 10 million reads prior to processing (NPre) (B) and normalizing 5 million reads prior to taxonomic profiling (NPost) (C) divided by sample. Red solid lines indicate the dsLib median per sample while the hashed red lines indicate the ssLib median per sample.
**Figure S24:** GC content distributions of oral reads by sample. Histograms showing the counts of oral reads at each %GC bin for the no normalization (NN) dataset (A), normalizing 10 million reads prior to processing (NPre) (B) and normalizing 5 million reads prior to taxonomic profiling (NPost) (C) divided by sample. Red solid lines indicate the dsLib median per sample while the hashed red lines indicate the ssLib median per sample.
**Figure S25:** Median read length and %GC of oral reads. Boxplots showing the median read length (bp) (A, C) and %GC (B, D) of the reads attributed to oral taxa from each sample for the no normalization (NN) dataset (A, B) and the NPre dataset subsampling to 10 million reads prior to read processing (C, D). Grey hashed lines connect the dsLib and ssLib of the same individual. Points are coloured by the percent of processed reads (post *nf‐core/eager* and Cutadpt) determined to be oral. Wilcoxon signed‐rank tests were used for paired comparisons. Significance codes: **p < 0.01. Effect sizes (rank‐biserial r): ††† large (≥ 0.50).
**Figure S26:** Alpha‐diversity analyses for read stratification datasets. Raincloud plots showing both the total observed species count (A, C) and Shannon diversity index (B, D) for the NPre and NPost dataset read length stratification (< 50 bp versus ≥ 50 bp): normalizing to 10 million reads prior to processing (A, B), and normalizing to 5 million reads prior to taxonomic profiling (C, D). For each plot, the left panel shows the alpha‐diversity metric for all reads classified by Kraken2, and the right panel shows the alpha‐diversity metric for only reads assigned to oral taxa. Wilcoxon signed‐rank tests were used for paired comparisons. Significance codes: ns, *p* ≥ 0.05; *p < 0.05; **p < 0.01. Effect sizes (rank‐biserial *r*): † small (< 0.30), †† moderate (0.30–0.49), ††† large (≥ 0.50).
**Figure S27:** Drivers of PCA structure for dataset normalizing 10 million reads prior to processing steps and stratifying by read length. Principal component analysis (PCA) of samples based on NPre Kraken2 species tables after normalizing to 10 million reads prior to processing via *nf‐core/eager* and stratifying by read length (< 50 bp versus ≥ 50 bp) for total species (A, B), oral species (C, D) and oral species of only well‐preserved samples (no BAN001, FUM003, GUE001, SPM001, or ZAF001). (A, C, E) Arrows indicate species loadings, with the direction and magnitude reflecting their contribution to PC1. Species with the strongest positive and negative loadings on PC1 are listed. (B, D, F) PCA highlighting species driving separation along PC2, with associated species loadings. Libraries from the same sample are connected by a hashed grey line. Asterisks next to species names in the total species (A, B) PCAs indicate oral associated taxa.
**Figure S28:** Drivers of PCA structure for dataset normalizing 5 million reads into Kraken2 and stratifying by read length. Principal component analysis (PCA) of samples based on NPost Kraken2 species tables after normalizing to 5 million reads and stratifying by read length (< 50 bp versus ≥ 50 bp) for total species (A, B), oral species (C, D), and oral species of only well‐preserved samples (no BAN001 or FUM003). A, C, E. Arrows indicate species loadings, with the direction and magnitude reflecting their contribution to PC1. Species with the strongest positive and negative loadings on PC1 are listed. B, D, F. PCA highlighting species driving separation along PC2, with associated species loadings. Libraries from the same sample are connected by a hashed grey line. Asterisks next to species names in the total species (A, B) PCAs indicate oral associated taxa.
**Figure S29:** *Methanocatella oralis* (27.5% GC, low GC taxa) per sample read length distributions and damage. (A) Bar plot displaying the total number of *Methanocatella oralis* reads per well‐preserved library subsampled to 5 million reads prior to taxonomic profiling (NPost). Only samples with over 100 reads were included. (B) Histograms showing the distribution of read length for all *M. oralis* reads for well‐preserved samples subsampled to 5 million reads prior to taxonomic profiling. Plots are grouped by individual. The solid red line indicates the dsLib median while the dashed line indicates the ssLib median. Insets to the right of each histogram show nucleotide misincorporation patterns to assess characteristic aDNA damage. The left panel displays the frequency of cytosine deamination at the 5′ end (C→T), and the right panel shows the complementary guanine to adenine (G→A) transitions at the 3′ end. Double‐stranded libraries exhibit complementary G→A substitutions at the 3′ end due to blunt‐end repair during library preparation (Briggs et al. 2007). In contrast, single‐stranded libraries show complementary C→T patterns at both ends because the DNA molecules are fully denatured and reconstructed without fill‐in or blunt‐end repair (Meyer et al. 2012). As a result, ssLibs typically display a flat profile at the 3′ end when plotting G→A transitions, since this misincorporation pattern is not expected in ssLib preparation.
**Figure S30:** *Neisseria elongata* (54% GC, mid GC taxa) per sample read length distributions and damage. (A) Bar plot displaying the total number of *Neisseria elongata* reads per well‐preserved library subsampled to 5 million reads prior to taxonomic profiling (NPost). Only samples with over 100 reads were included. (B) Histograms showing the distribution of read length for all *N. elongata* reads for well‐preserved samples subsampled to 5 million reads prior to taxonomic profiling. Plots are grouped by individual. The solid red line indicates the dsLib median while the dashed line indicates the ssLib median. Insets to the right of each histogram show nucleotide misincorporation patterns to assess characteristic aDNA damage. The left panel displays the frequency of cytosine deamination at the 5′ end (C→T), and the right panel shows the complementary guanine to adenine (G→A) transitions at the 3′ end. Double‐stranded libraries exhibit complementary G→A substitutions at the 3′ end due to blunt‐end repair during library preparation (Briggs et al. 2007). In contrast, single‐stranded libraries show complementary C→T patterns at both ends because the DNA molecules are fully denatured and reconstructed without fill‐in or blunt‐end repair (Meyer et al. 2012). As a result, ssLibs typically display a flat profile at the 3′ end when plotting G→A transitions, since this misincorporation pattern is not expected in ssLib preparation.
**Figure S31:** *Flexilinea* sp001717545 (55% GC, mid GC taxa) per sample read length distributions and damage. (A) Bar plot displaying the total number of *Flexilinea* sp001717545 (*Anaerolineaceae* bacterium oral taxon 439) reads per well‐preserved library subsampled to 5 million reads prior to taxonomic profiling (NPost). Only samples with over 100 reads were included. (B) Histograms showing the distribution of read length for all *Flexilinea* sp001717545 reads for well‐preserved samples subsampled to 5 million reads prior to taxonomic profiling. Plots are grouped by individual. The solid red line indicates the dsLib median while the dashed line indicates the ssLib median. Insets to the right of each histogram show nucleotide misincorporation patterns to assess characteristic aDNA damage. The left panel displays the frequency of cytosine deamination at the 5′ end (C→T), and the right panel shows the complementary guanine to adenine (G→A) transitions at the 3′ end. Double‐stranded libraries exhibit complementary G→A substitutions at the 3′ end due to blunt‐end repair during library preparation (Briggs et al. 2007). In contrast, single‐stranded libraries show complementary C→T patterns at both ends because the DNA molecules are fully denatured and reconstructed without fill‐in or blunt‐end repair (Meyer et al. 2012). As a result, ssLibs typically display a flat profile at the 3′ end when plotting G→A transitions, since this misincorporation pattern is not expected in ssLib preparation.
**Figure S32:** *Lautropia mirabilis* (65.5% GC, high GC taxa) per sample read length distributions and damage. (A) Bar plot displaying the total number of *Lautropia mirabilis* reads per well‐preserved library subsampled to 5 million reads prior to taxonomic profiling (NPost). Only samples with over 100 reads were included. (B) Histograms showing the distribution of read length for all *L. mirabilis* reads for well‐preserved samples subsampled to 5 million reads prior to taxonomic profiling. Plots are grouped by individual. The solid red line indicates the dsLib median while the dashed line indicates the ssLib median. Insets to the right of each histogram show nucleotide misincorporation patterns to assess characteristic aDNA damage. The left panel displays the frequency of cytosine deamination at the 5′ end (C→T), and the right panel shows the complementary guanine to adenine (G→A) transitions at the 3′ end. Double‐stranded libraries exhibit complementary G→A substitutions at the 3′ end due to blunt‐end repair during library preparation (Briggs et al. 2007). In contrast, single‐stranded libraries show complementary C→T patterns at both ends because the DNA molecules are fully denatured and reconstructed without fill‐in or blunt‐end repair (Meyer et al. 2012). As a result, ssLibs typically display a flat profile at the 3′ end when plotting G→A transitions, since this misincorporation pattern is not expected in ssLib preparation.
**Figure S33:** *Actinomyces dentalis* (73% GC, high GC taxa) per sample read length distributions and damage. (A) Bar plot displaying the total number of *Actinomyces dentalis* reads per well‐preserved library subsampled to 5 million reads prior to taxonomic profiling (NPost). Only samples with over 100 reads were included. (B) Histograms showing the distribution of read length for all *A. dentalis* reads for well‐preserved samples subsampled to 5 million reads prior to taxonomic profiling. Plots are grouped by individual. The solid red line indicates the dsLib median while the dashed line indicates the ssLib median. Insets to the right of each histogram show nucleotide misincorporation patterns to assess characteristic aDNA damage. The left panel displays the frequency of cytosine deamination at the 5′ end (C→T), and the right panel shows the complementary guanine to adenine (G→A) transitions at the 3′ end. Double‐stranded libraries exhibit complementary G→A substitutions at the 3′ end due to blunt‐end repair during library preparation (Briggs et al. 2007). In contrast, single‐stranded libraries show complementary C→T patterns at both ends because the DNA molecules are fully denatured and reconstructed without fill‐in or blunt‐end repair (Meyer et al. 2012). As a result, ssLibs typically display a flat profile at the 3′ end when plotting G→A transitions, since this misincorporation pattern is not expected in ssLib preparation.
**Figure S34:** *Aggregatibacter kilianii* (43% GC, low GC taxa) per sample read length distributions and damage. (A) Bar plot displaying the total number of *Aggregatibacter kilianii* reads per well‐preserved library subsampled to 5 million reads prior to taxonomic profiling (NPost). Only samples with over 100 reads were included. (B) Histograms showing the distribution of read length for all *A. kilianii* reads for well‐preserved samples subsampled to 5 million reads prior to taxonomic profiling. Plots are grouped by individual. The solid red line indicates the dsLib median while the dashed line indicates the ssLib median. Insets to the right of each histogram show nucleotide misincorporation patterns to assess characteristic aDNA damage. The left panel displays the frequency of cytosine deamination at the 5′ end (C→T), and the right panel shows the complementary guanine to adenine (G→A) transitions at the 3′ end. Double‐stranded libraries exhibit complementary G→A substitutions at the 3′ end due to blunt‐end repair during library preparation (Briggs et al. 2007). In contrast, single‐stranded libraries show complementary C→T patterns at both ends because the DNA molecules are fully denatured and reconstructed without fill‐in or blunt‐end repair (Meyer et al. 2012). As a result, ssLibs typically display a flat profile at the 3′ end when plotting G→A transitions, since this misincorporation pattern is not expected in ssLib preparation.
**Figure S35:** Median GC% and read length of NPost mapped reads. Plots showing the median GC content (%) and fragment length (bp) of reads mapped to the following species: (A) *Methanocatella oralis*, (B) *Aggregatibacter kilianii* (C) *Neisseria elongata* , (D) *Flexilinea* sp001717545 (*Anaerolineaceae* bacterium oral taxon 439), (E) *Lautropia mirabilis* and (F) *Actinomyces dentalis* . The average GC content (%) is displayed next to each species name, and plots are arranged in order of ascending GC. The red dashed line on the plot displays the median GC% of each reference genome. Library type is denoted by colour and preservation is denoted by shape. Grey lines connect libraries from the same individual, and individual names are placed next to the dsLib in the pair.
**Figure S36:** Percent of duplicated reads classified by Kraken2. Bar plots displaying the percentage of reads classified by Kraken2 that are duplicated. Duplication is separated into broader categories (unique/no duplication, duplicated twice, duplicated 3–5 times, duplicated 6–10 times, or duplicated greater than 10 times). Plots are divided between merged and unmerged reads for the no normalization (NN) dataset (A), normalizing 10 million reads prior to processing (NPre) (B), and normalizing 5 million reads prior to taxonomic profiling (NPost) (C).
**Figure S37:** Percent of duplicated oral reads. Bar plots displaying the percentage of oral reads that are duplicated. Duplication is separated into broader categories (unique/no duplication, duplicated twice, duplicated 3–5 times, duplicated 6–10 times, or duplicated greater than 10 times). Plots are divided between merged and unmerged reads for the no normalization (NN) dataset (A), normalizing 10 million reads prior to processing (NPre) (B), and normalizing 5 million reads prior to taxonomic profiling (NPost) (C).

**Table S1A:** Metadata for publicly available sequencing data used in this study.
**Table S1B:** Metadata for newly sequenced data used in this study.
**Table S1C:** Control samples used in this study.
**Table S1D:** Publicly available comparative data used in this study.
**Table S1E:** Additional sequences added to the human reference genome (hs37d5) for mapping during processing steps.
**Table S1F:** GTDB r207 oral species list.

**Table S2A:** Wilcoxon *t*‐tests and associated figures.
**Table S2B:** PERMANOVA stats and associated figures.
**Table S2C:** PCA loadings.
**Table S3A:** No normalization (NN) initial read count and processing read retention.

**Table S3B:** Nonpareil current coverage and estimated sequencing depth to achieve 95× coverage (NPre).
**Table S3C:** SourceTracker results after normalizing to 10M reads prior to processing and taxonomic profiling with Kraken2 (NPre).
**Table S3D:** Read counts throughout processing steps after normalizing to 10M reads prior to processing (NPre).

**Table S4A:** Median read length (bp) throughout processing steps after normalizing to 10M reads (NPre).
**Table S4B:** Median GC% throughout processing steps after normalizing to 10M reads (NPre).
**Table S4C:** GOY005 read length (bp) distributions throughout processing steps after normalizing to 10M reads (NPre).
**Table S4D:** GOY005 GC content (%) distributions throughout processing steps after normalizing to 10M reads (NPre).
**Table S4E:** Comparing median read length throughout *nf‐core/eager* processing steps after normalizing to 10M reads (NPre) when keeping or removing the 75 bp peak (reads longer than > 140 bp that could not be collapsed).
**Table S4F:** Analysis of post‐AdapterRemoval fragment length < 30 bp in ssLibs after normalizing to 10M reads prior to processing (NPre).

**Table S5A:** Kraken2 total species table after normalizing to 5M reads prior to taxonomic profiling (NPost).
**Table S5B:** Median read length (bp) and GC% of classified reads after normalizing to 5M reads prior to taxonomic profiling (NPost, no read length subsampling).
**Table S5C:** GOY005 classified reads read length (bp) distributions (NPost).
**Table S5D:** GOY005 classified reads GC content (%) distributions (NPost).
**Table S5E:** Total species alpha‐diversity metrics after normalizing to 5M reads prior to taxonomic profiling (NPost).
**Table S5F:** Principal components analysis of total classified reads after normalizing to 5M reads prior to taxonomic profiling (NPost, no read length subsampling).

**Table S6A:** Kraken2 oral species table after normalizing to 5M reads prior to taxonomic profiling (NPost).
**Table S6B:** Median read length and GC% of oral reads after normalizing to 5M reads prior to taxonomic profiling (NPost, no read length subsampling).
**Table S6C:** GOY005 read length (bp) distributions of oral reads after normalizing to 5M reads prior to taxonomic profiling (NPost).
**Table S6D:** GOY005 GC% distributions of oral reads after normalizing to 5M reads prior to taxonomic profiling (NPost).
**Table S6E:** Oral species alpha‐diversity metrics after normalizing to 5M reads prior to taxonomic profiling (NPost).
**Table S6F:** Principal components analysis of oral reads after normalizing to 5M reads prior to taxonomic profiling (NPost, read length subsampling).

**Table S7A:** Principal components analysis of well‐preserved samples normalized to 5M reads prior to taxonomic profiling (NPost) and filtered to include only reads from oral associated taxa.
**Table S7B:** Top 10 loadings for the principal components analysis of well‐preserved samples normalized to 5M reads prior to taxonomic profiling (NPost) and filtered to include only reads from oral associated taxa.
**Table S7C:** Kraken2 taxIDs for the oral species making up the top 10 loadings of each PC (NPost well‐preserved oral PCA).
**Table S7D:** Read length distributions of *Aggregatibacter kilianii* GOY005 reads after normalizing to 5M reads prior to taxonomic profiling (NPost).
**Table S7E:** Read length distributions of *Neisseria elongata* GOY005 reads after normalizing to 5M reads prior to taxonomic profiling (NPost).
**Table S7F:** Read length distributions of *Lautropia mirabilis* GOY005 reads after normalizing to 5M reads prior to taxonomic profiling (NPost).

## Data Availability

All publicly available data used in this manuscript are available from their original sources, as cited in the manuscript. All newly generated sequencing data are available through the European Nucleotide Archive under project accession PRJEB112518. Scripts and code used in the project are available on GitHub: https://github.com/keriburge/library_ds_vs_ss.
