# Supplementary material for: Comparing the Performance of Double‐Stranded and Single‐Stranded DNA Libraries for Ancient Oral Microbiome Reconstruction
Source: Mol Ecol Resour. 2026 Sep 21;26(7):e70201. doi: 10.1111/1755-0998.70201 (PMC13593575; doi:10.1111/1755-0998.70201)
Supplement: Supplementary file 1 [file MEN-26-e70201-s001.pdf]

Supplemental materials for

Comparing the performance of double-stranded and single-stranded DNA libraries for ancient oral microbiome reconstruction

Authors: Keri Burge, Irina M. Velsko, Domingo C. Salazar-García, María Haber Uriarte, Joaquín Lomba Maurandi, Christina Warinner

Table of Contents:

Additional Results 3

S3.1 Sequencing read characteristics and taxonomic diversity prior to oral filtering 3

S3.2 Differences in taxonomic identification based on read length prior to oral filtering 4

S3.3 Clonality prior to oral filtering 4

Supplemental Figures 5

3.2 Library complexity 5

Supplemental Figure 1. Estimated metagenomic dataset coverage for samples normalized to 10 million reads going into *nf-core/eager* (NPre dataset) 5

Supplemental Figure 2. Estimated metagenomic dataset coverage for samples not normalized going into *nf-core/eager* (NN dataset) 6

3.3 Contamination profiles 7

Supplemental Figure 3. Sample preservation status with no normalization (NN dataset). 7

Supplemental Figure 4. Normalizing 10 million reads into *nf-core/eager* (NPre) sample preservation status 8

Supplemental Figure 5. Normalizing 5 million reads into Kraken2 (NPost) sample preservation status 9

Supplemental Figure 6. SourceTracker plots show source composition of each sample 10

Supplemental Figure 7. Rarefied PCoAs of ancient dental calculus and source samples 11

3.4 Effects of library preparation protocol on read processing outcomes 12

Supplemental Figure 8. CDM read length and GC content throughout processing steps 12

Supplemental Figure 9. Read length and GC content throughout processing steps for poorly preserved samples BAN, GUE, and ZAF 13

Supplemental Figure 10. Read length and GC content throughout processing steps for poorly preserved FUM samples 14

Supplemental Figure 11. Read length and GC content throughout processing steps for GDN, GOY, and VLD 15

Supplemental Figure 12. PES and SPM read length and GC content throughout processing steps 16

Supplemental Figure 13. Read retention and processing outcomes in *nf-core/eager* after subsampling to 10 million reads (NPre dataset) 17

3.5 Oral microbiome–focused diversity and compositional analyses 18

Supplemental Figure 14. Sequencing read characteristics and taxonomic diversity of

samples profiled with Kraken2 after subsampling to 5M reads (NPost) 18
Supplemental Figure 15. Read lengths distributions of classified reads by sample 19
Supplemental Figure 16. GC content distributions of classified reads by sample 20
Supplemental Figure 17. Median read length and %GC of classified Kraken2 reads 21
Supplemental Figure 18. Alpha-diversity analyses for both total classified reads and oral reads 22
Supplemental Figure 19. Drivers of PCA structure for dataset normalizing 10 million reads prior to processing steps 23
Supplemental Figure 20. Drivers of PCA structure for the no normalization dataset 24
Supplemental Figure 21. Drivers of PCA structure for dataset normalizing 5 million reads into Kraken2 (NPost) 25
Supplemental Figure 22. Total species beta diversity and dispersion of well-preserved samples from the NPost dataset (subsampled to 5M reads prior to taxonomic profiling) 26
Supplemental Figure 23. Read lengths distributions of oral reads by sample 27
Supplemental Figure 24. GC content distributions of oral reads by sample 28
Supplemental Figure 25. Median read length and %GC of oral reads 29
3.6 Differences in taxonomic identification based on read length 30
Supplemental Figure 26. Alpha-diversity analyses for read stratification datasets 30
Supplemental Figure 27. Drivers of PCA structure for dataset normalizing 10 million reads prior to processing steps and stratifying by read length 31
Supplemental Figure 28. Drivers of PCA structure for dataset normalizing 5 million reads into Kraken2 and stratifying by read length 32
3.7 Oral species-level drivers of compositional differences between library protocols 33
Supplemental Figure 29. *Methanocatella oralis* (27.5% GC, low GC taxa) per sample read length distributions and damage 33
Supplemental Figure 30. *Neisseria elongata* (54% GC, mid GC taxa) per sample read length distributions and damage 34
Supplemental Figure 31. *Flexilinea* sp001717545 (55% GC, mid GC taxa) per sample read length distributions and damage 35
Supplemental Figure 32. *Lautropia mirabilis* (65.5% GC, high GC taxa) per sample read length distributions and damage 36
Supplemental Figure 33. *Actinomyces dentalis* (73% GC, high GC taxa) per sample read length distributions and damage 37
Supplemental Figure 34. *Aggregatibacter kilianii* (43% GC, low GC taxa) per sample read length distributions and damage 38
Supplemental Figure 35. Median GC% and read length of NPost mapped reads 39
3.8 Clonality 40
Supplemental Figure 36. Percent of duplicated reads classified by Kraken2 40
Supplemental Figure 37. Percent of duplicated oral reads 41
References **42**

Additional Results

S3.1 Sequencing read characteristics and taxonomic diversity prior to oral filtering

Because the substantial loss of ssLib reads during NPre data processing resulted in highly uneven read counts available for taxonomic classification, we used the normalized NPost dataset to test for library-specific differences in taxonomic composition. In the NPost dataset, libraries were subsampled to 5 million reads prior to taxonomic classification with Kraken2 (**Table S5A**). On average, a greater proportion of reads from dsLibs were classified to the species level (14.3%, SD = 9.20) compared to ssLibs (9.8%, SD = 6.74) (p = 0.004; effect size = 0.85; **Table S5B**). Classified reads from dsLibs were also significantly longer (median = 64 bp, SD = 7.74) than those from ssLibs (median = 53.5 bp, SD = 6.17) (p = 0.006; effect size = 0.89; **Figure S14A; Table S5B**). In addition, classified dsLib reads exhibited higher GC content (median = 61%, SD = 4.84) than classified ssLib reads (median = 51%, SD = 7.16) (p = 0.006; effect size = 0.89; **Figure S14B; Table S5B**). Neanderthal GOY005 is shown as an example (**Figure S14C-D; Table S5C-D**). The same trends were also seen in the NN and NPre datasets (**Figures S15-17**).

We next assessed alpha diversity across the NPost dataset. No significant difference was observed in the total number of species recovered between library preparation protocols (p = 0.407; effect size = 0.32; **Figure S14E; Table S5E**), with dsLibs and ssLibs recovering an average of 312 (SD = 96.40) and 315 (SD = 96.70) species, respectively. The same trend was seen in the NPre dataset (**Figure S18C**), but dsLibs recovered significantly more species in the NN dataset (**Figure S18A**), as expected given the three dsLibs with elevated sequencing depth in the NN dataset. In contrast, Shannon diversity was significantly higher in ssLibs (mean = 3.58, SD = 0.84) compared to dsLibs (mean = 3.35, SD = 0.80) (p = 0.004; effect size = 0.89; **Figure S14F; Table S5E**), indicating a slightly more even distribution of species abundances in ssLibs. The same trend was observed in the NN dataset (**Figure S18B**) but not in the NPre dataset (**Figure S18D**).

Beta diversity differences in community composition were evaluated using principal component analysis (PCA) of species-level taxonomic profiles. Samples clustered significantly by individual but not by library preparation protocol across all NPost datasets (**Figure S14G; Table S5F**), and the same trend was observed for the NPre (**Figure S19A-B**) and NN (**Figure S20A-B**) datasets. Preservation status and community composition were significantly associated, with well-preserved samples plotting in positive PC1 space and poorly preserved samples plotting in negative PC1 space (**Figure S14G**). Taxa with strong positive PC1 loadings included oral-associated species such as CAJPSE01 sp905373185 (an uncultured *Fretibacterium* sp.), *Fretibacterium fastidiosum*, and *Desulfomicrobium orale*, whereas negative PC1 loadings were associated with taxa commonly interpreted as contaminants, including *Pelomonas puraquae*, *Staphylococcus epidermidis*, and *Aquabacterium commune*, which correlates with the preservation status of the samples. (**Figure S21A**). Although library type contributed less to overall variance, separation along PC1 between samples from the same individual was partially

associated with library protocol, with ssLibs tending toward more positive PC1 scores and dsLibs toward more negative scores. However, PC loadings overall are driven more by individual than library type, indicating that sample specific compositions are more influential than the low level of species differences between library types (**Figure S22**).

S3.2 Differences in taxonomic identification based on read length prior to oral filtering

To further explore the influence of fragment length on taxonomic classification, we conducted parallel analyses on datasets restricted to total classified reads < 50 bp and total classified reads ≥ 50 bp for both the NPre and NPost datasets. This is prior to oral filtering (oral filtering analyses are reported in Section 3.6).

As seen with oral reads, read length stratification did not result in significant differences in alpha diversity for ssLibs (**Figure S26C**). For dsLibs, in the NPre dataset, dsLibs reads ≥ 50 bp did recover significantly more species than reads < 50 bp (p = 0.006, effect size = 0.88) (**Figure S27A**). A similar trend was observed for the NPost dataset, although it was not significant (p = 0.463, effect size = 0.47) (**Figure S27C**). Shannon diversity (**Figure S27D**) did not significantly differ between read length classes. For total species, no significant differences were observed for dsLibs (p = 0.846; effect size = 0.08) or ssLibs (p = 0.367; effect size = 0.37). The same trends for Shannon diversity were observed in the NPre dataset (**Figure S27B**).

S3.3 Clonality prior to oral filtering

To assess clonality in classified reads prior to oral filtering, we calculated the proportion of unique reads among all Kraken2-classified reads, irrespective of taxonomic rank. On average, across the NPost dataset, classified reads from dsLibs consisted of 87.0% (SD = 22.0) unique sequences (mean = 1,205,138 unique reads, SD = 719,142), whereas ssLibs consisted of 94.4% (SD = 5.53) unique sequences (mean = 865,195 unique reads, SD = 518202) (p = 0.006; effect size = 0.82; **Figure S36C**). This was additionally seen in the NN and NPre datasets (**Figure S36A-B**). As seen with the oral reads dataset (Section 3.8), clonality in total classified reads additionally varied substantially across samples. GDN001 likewise represented a clear outlier in both library preparation protocols, exhibiting elevated levels of total classified read duplication relative to other samples (**Figure S36**).

Supplemental Figures

3.2 Library complexity

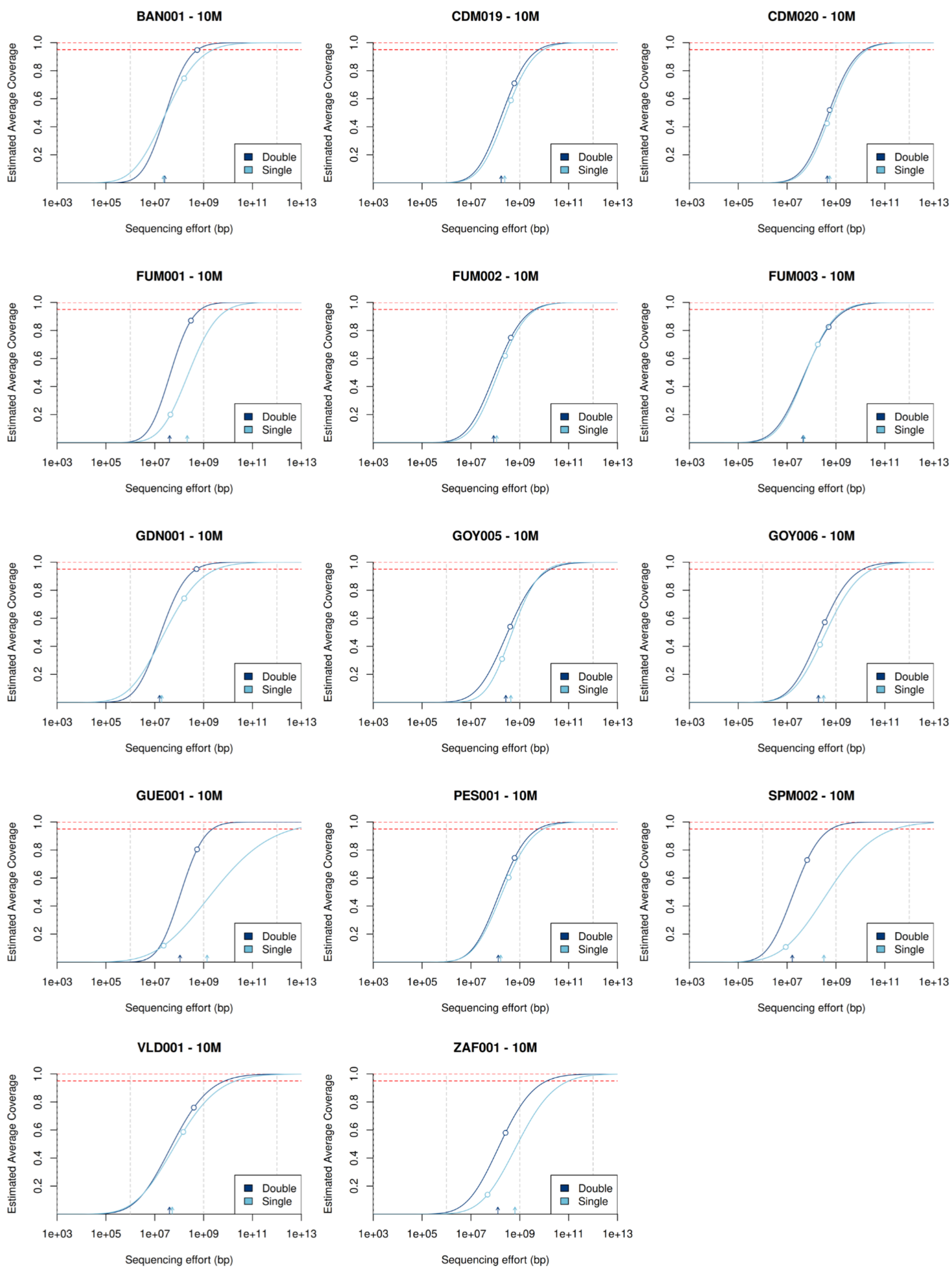

Supplemental Figure 1. Estimated metagenomic dataset coverage for samples normalized to 10 million reads going into *nf-core/eager* (NPre dataset). Nonpareil curves for each sample showing the estimated coverage in each metagenomic dataset. The horizontal dashed lines indicate 100 (gray) and 95% (red) coverage. The empty circles indicate the size and estimated average coverage of the datasets, and the lines after that point are projections of the fitted model. Arrows on the x-axis represent the location corresponding to exp(Nd), which is a metric of sequence diversity based on the projection of the Nonpareil curve. Curves with corresponding arrows that are more to the right indicate a higher sequence diversity. Each plot shows both the dsLib (dark blue) and ssLib (light blue) of each sample.

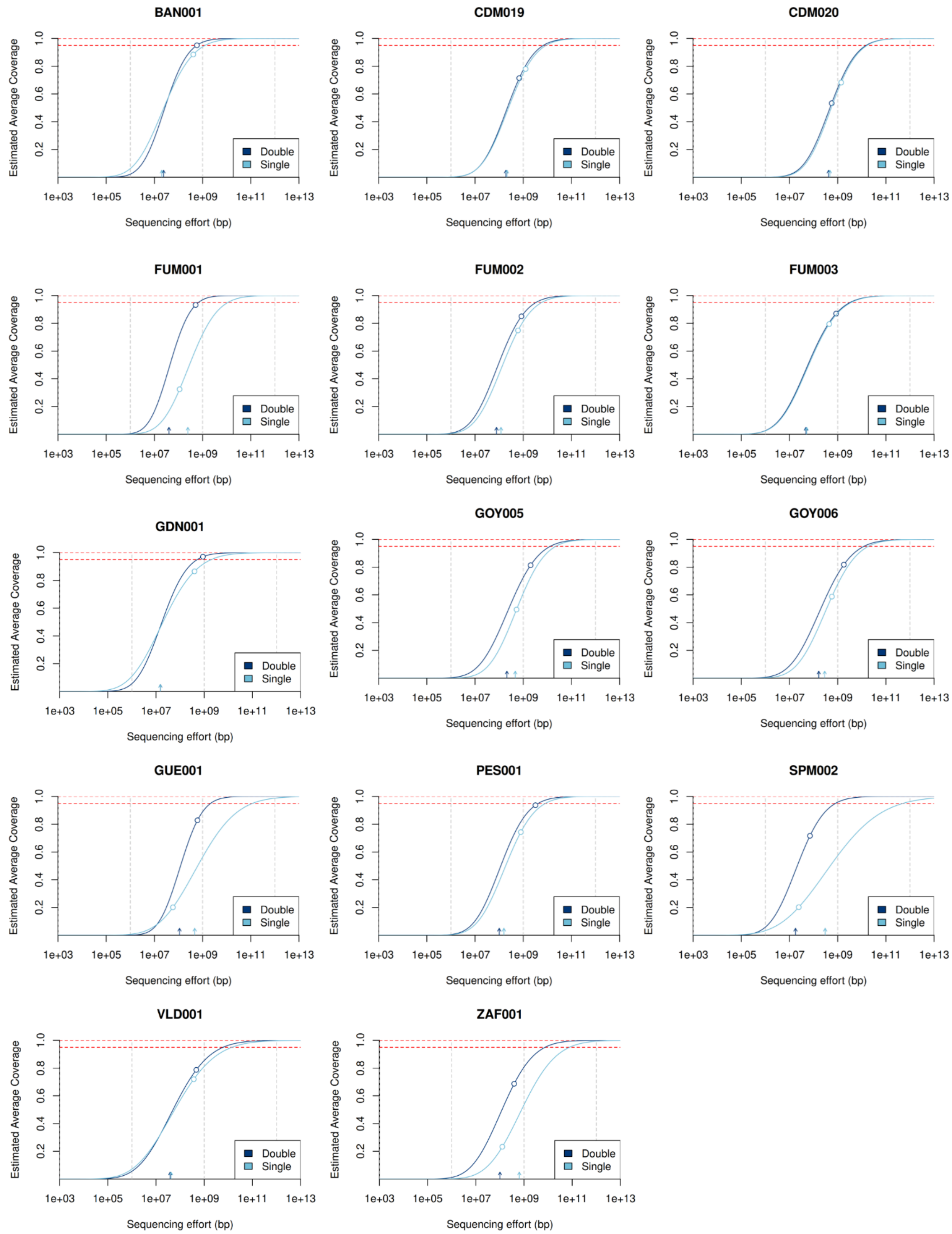

Supplemental Figure 2. Estimated metagenomic dataset coverage for samples not normalized going into *nf-core/eager* (NN dataset).

Nonpareil curves for each sample showing the estimated coverage in each metagenomic dataset. The horizontal dashed lines indicate 100 (gray) and 95% (red) coverage. The empty circles indicate the size and estimated average coverage of the datasets, and the lines after that point are projections of the fitted model. Arrows on the x-axis represent the location corresponding to exp(Nd), which is a metric of sequence diversity based on the projection of the Nonpareil curve. Curves with corresponding arrows that are more to the right indicate a higher sequence diversity. Each plot shows both the dsLib (dark blue) and ssLib (light blue) of each sample.

3.3 Contamination profiles

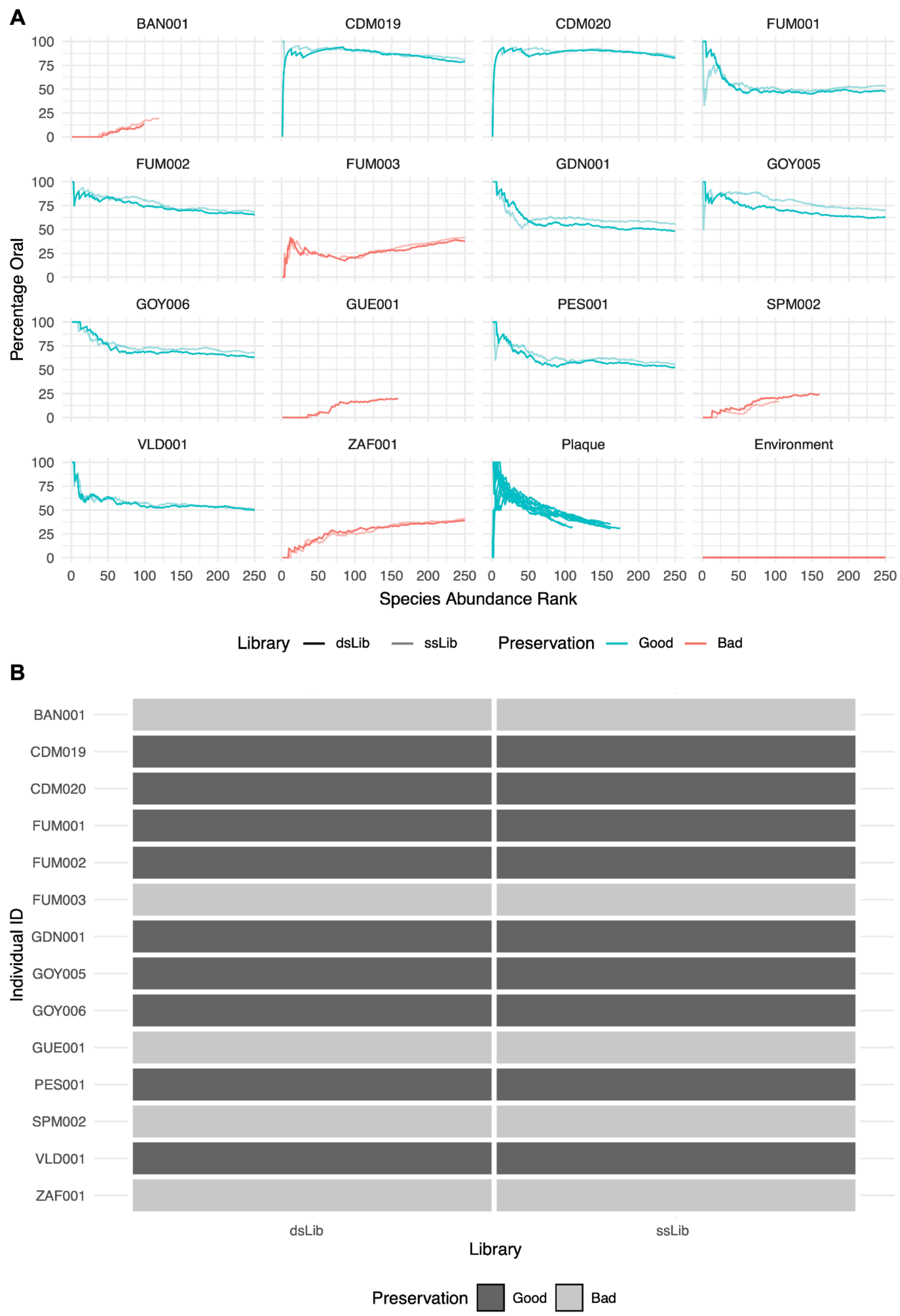

Supplemental Figure 3. Sample preservation status with no normalization (NN dataset).

A. Cumulative percent decay (cuperdec) plots showing the fraction of oral taxa relative to all taxa, as ordered by abundance rank for the sample set that was not normalized going into both *nf-core/eager* and Kraken2 (NN dataset). The x-axis is restricted to 250 positions for visualization purposes. Modern reference datasets (plaque and environmental samples) are shown in the bottom row for comparison. Each plot shows the cuperdec curve for both the dsLib (darker line) and ssLib (lighter, more transparent line) of each sample. A 50% threshold with an adaptive burin determines a passing sample. **B.** Summary of preservation status by individual and library strandedness. Each row represents an individual, with bars indicating whether dsLibs and/or ssLibs were classified as well (dark grey) or poorly preserved (light grey) as determined by the cuperdec curves **(A)**.

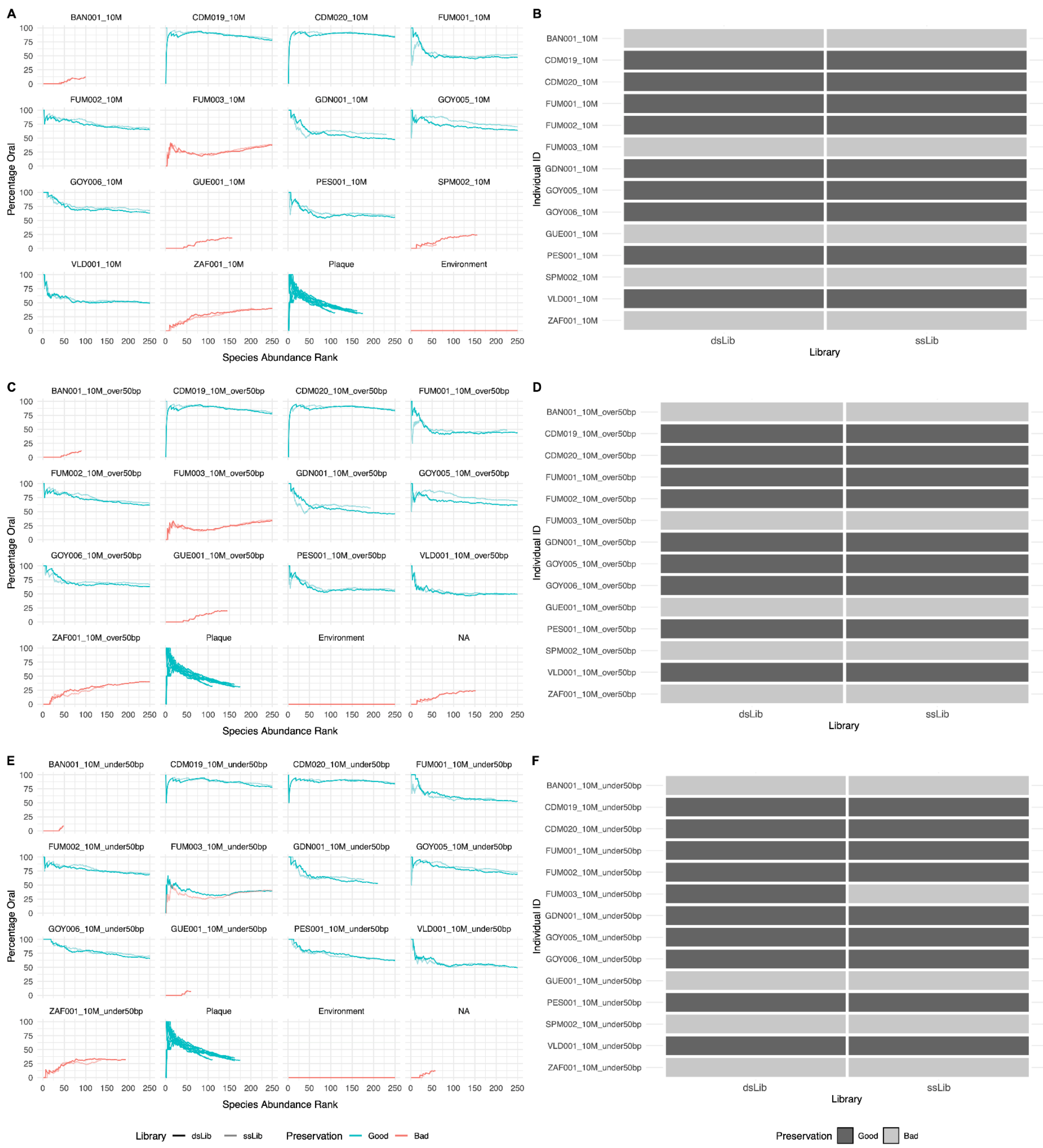

Supplemental Figure 4. Normalizing 10 million reads into *nf-core/eager* (NPre) sample preservation status.

A,C,E. Cumulative percent decay (cuperdec) plots showing the fraction of oral taxa relative to all taxa, as ordered by abundance rank for the NPre dataset that was normalized 10 million reads prior to processing with *nf-core/eager* **(A)** as well as further subsampled datasets separating reads ≥ 50bp **(C)** and reads < 50bp **(E)**. The x-axis is restricted to 250 positions for visualization purposes. Modern reference datasets (plaque and environmental samples) are shown in the bottom row for comparison. Each plot shows the cuperdec curve for both the dsLib (darker line) and ssLib (lighter, more transparent line) of each sample. A 50% threshold with an adaptive burin determines a passing sample. **B,D,F.** Summary of preservation status by individual and library strandedness. Each row represents an individual, with bars indicating whether dsLibs and/or ssLibs were classified as well (dark grey) or poorly preserved (light grey) as determined by the cuperdec curves. Summary plots are included for all reads **(B)**, reads ≥ 50bp **(D)**, and reads < 50bp **(F)**.

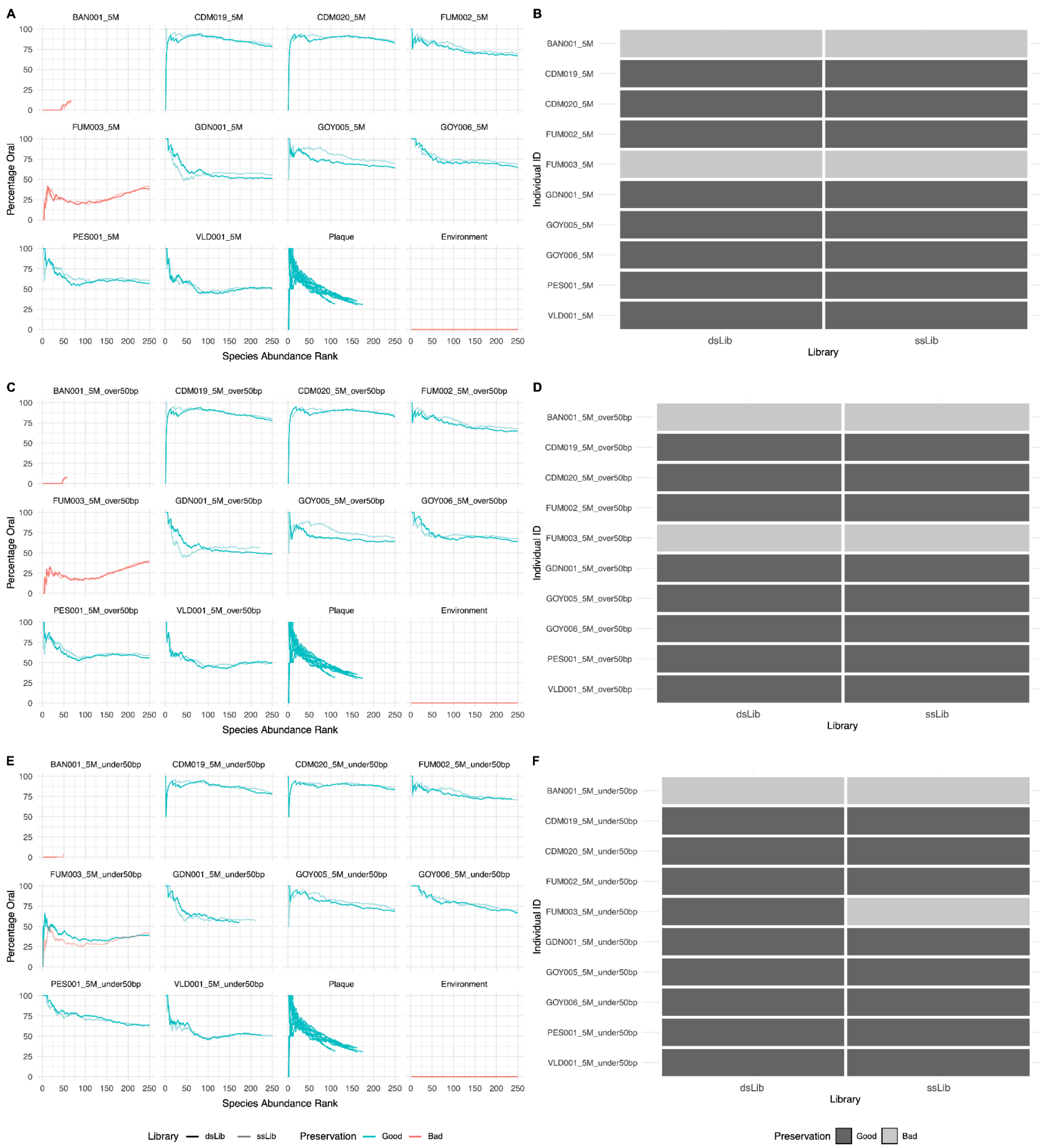

Supplemental Figure 5. Normalizing 5 million reads into Kraken2 (NPost) sample preservation status.

A,C,E. Cumulative percent decay (cuperdec) plots showing the fraction of oral taxa relative to all taxa, as ordered by abundance rank for the NPost dataset that was normalized 5 million reads prior to taxonomic profiling with Kraken2 **(A)** as well as further subsampled datasets separating reads ≥ 50bp **(C)** and reads < 50bp **(E)**. The x-axis is restricted to 250 positions for visualization purposes. Modern reference datasets (plaque and environmental samples) are shown in the bottom row for comparison. Each plot shows the cuperdec curve for both the dsLib (darker line) and ssLib (lighter, more transparent line) of each sample. A 50% threshold with an adaptive burin determines a passing sample. **B,D,F.** Summary of preservation status by individual and library strandedness. Each row represents an individual, with bars indicating whether dsLibs and/or ssLibs were classified as well (dark grey) or poorly preserved (light grey) as determined by the cuperdec curves. Summary plots are included for all reads **(B)**, reads ≥ 50bp **(D)**, and reads < 50bp **(F)**.

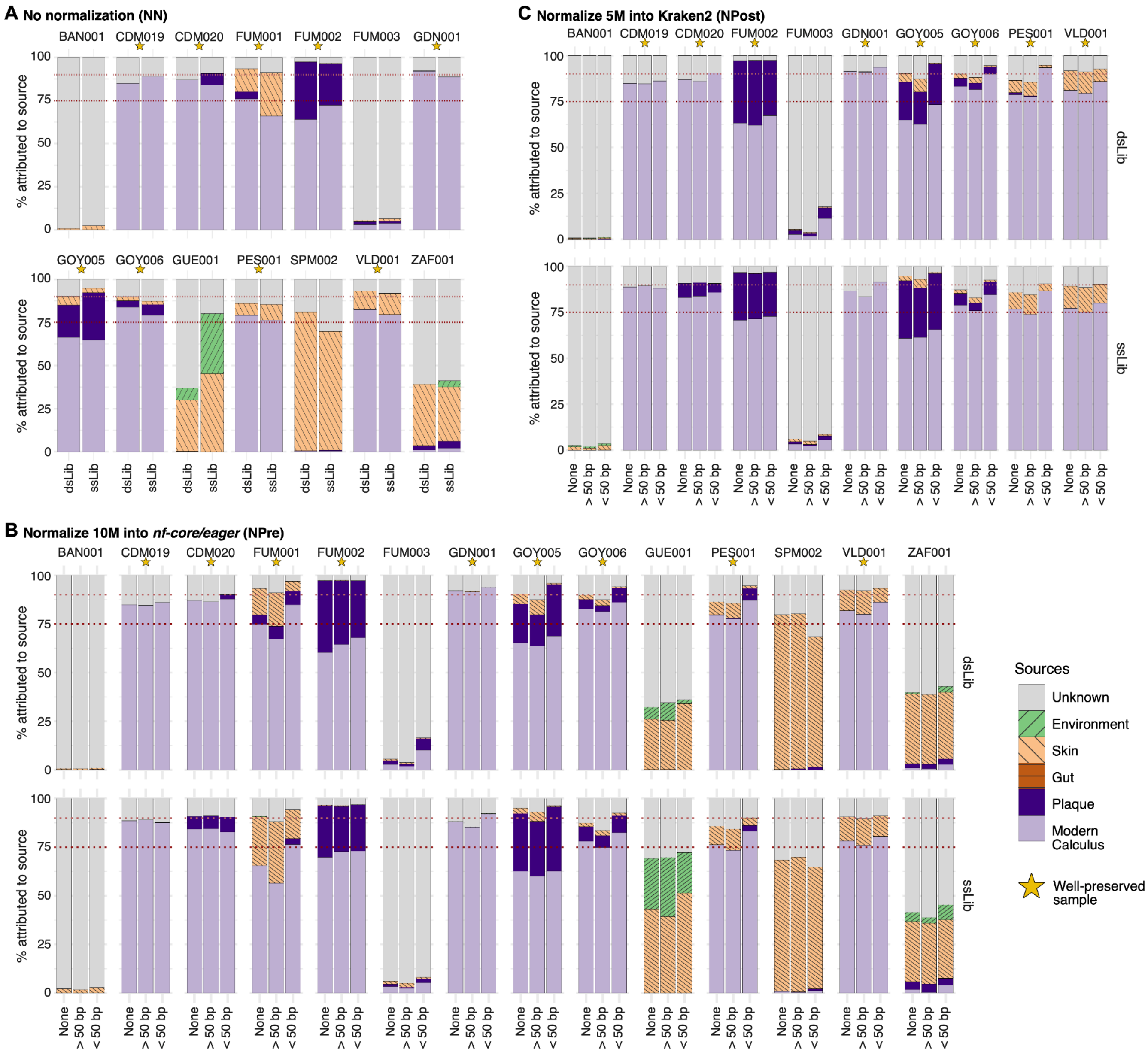

Supplemental Figure 6. SourceTracker plots show source composition of each sample.

Percentage bar plots showing the percentage of sources (environment, skin, gut, modern dental plaque, modern dental calculus, and unknown) attributed to each metagenomic sample for each normalization tract: no normalization (NN) **(A)**, normalizing to 10 million reads prior to processing (NPre) **(B)**, and normalizing to 5 million reads prior to taxonomic profiling (NPost) **(C)**. The red hashed line indicates 75% while the pink hashed line indicates 90%. Stars next to the sample name indicates a well-preserved sample. For the two normalization datasets **(C, D)**, read length stratification datasets (<50 bp versus ≥50 bp) are additionally shown.

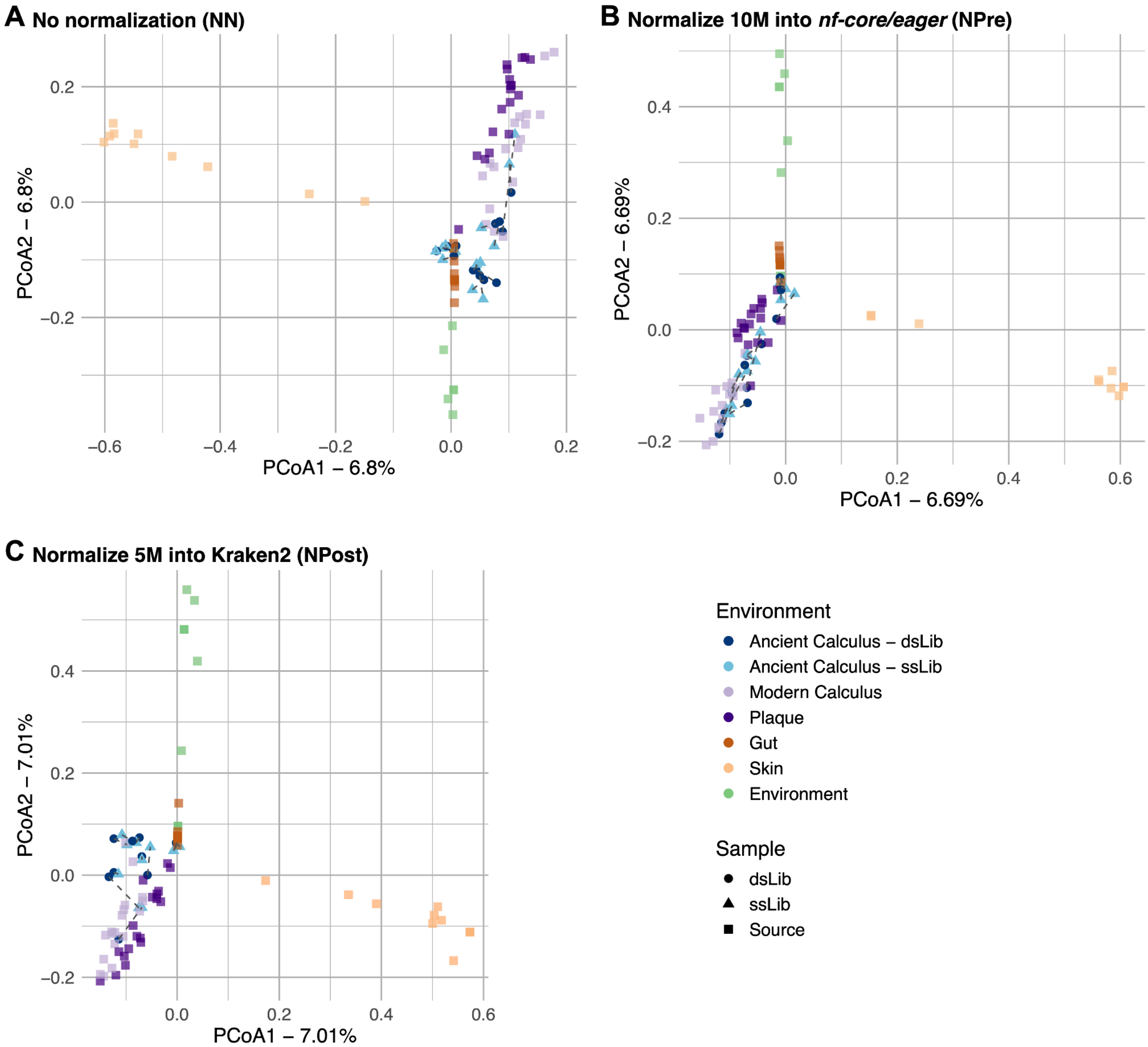

Supplemental Figure 7. Rarefied PCoAs of ancient dental calculus and source samples.

Principal coordinates analysis (PCoA) for ancient dental calculus samples and source samples (environment, skin, gut, modern dental plaque, and modern calculus) for each normalization dataset: no normalization (NN) **(A)**, normalizing to 10 million reads prior to processing (NPre) **(B)**, and normalizing to 5 million reads prior to taxonomic profiling (NPost) **(C)**. The variance explained by each principal coordinate is written on the axes. Dark grey hashed lines connect the dsLib and ssLib from the same individual. The Kraken2 total species tables used for these analyses were filtered, removing low abundance taxa, before plotting.

3.4 Effects of library preparation protocol on read processing outcomes

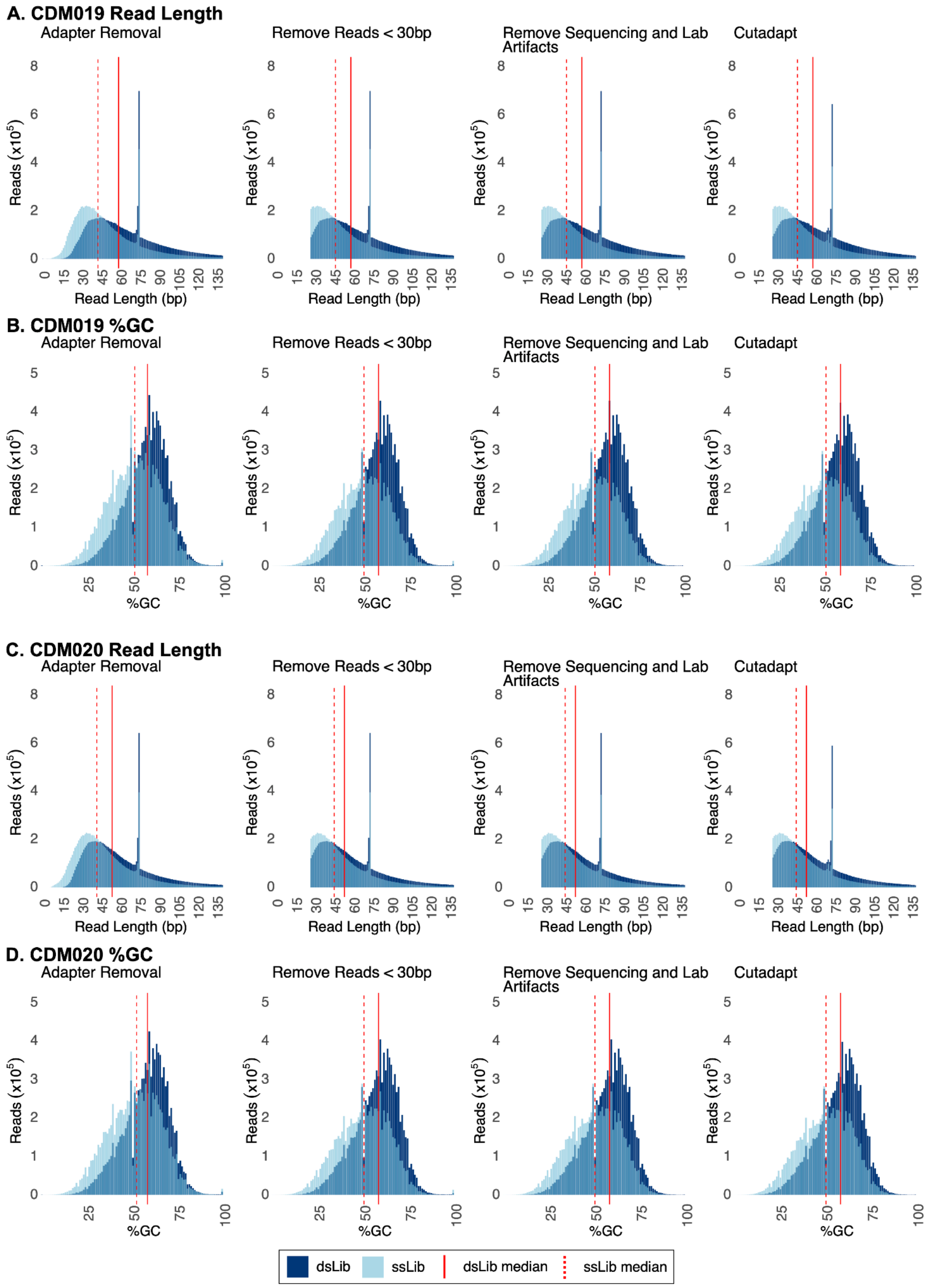

Supplemental Figure 8. CDM read length and GC content throughout processing steps.

Histograms showing the distribution of read length (bp) (**A,C**) and GC content (%) (**B,D**) for NPre CDM019 (**A,B**) and CDM020 (**C,D**) throughout the main read processing steps: adapter removal and merging, removing reads less than 30bp, removing sequencing and laboratory artifacts, and additional adapter removal with Cutadapt. The solid red line indicates the dsLib median while the dashed line indicates the ssLib median.

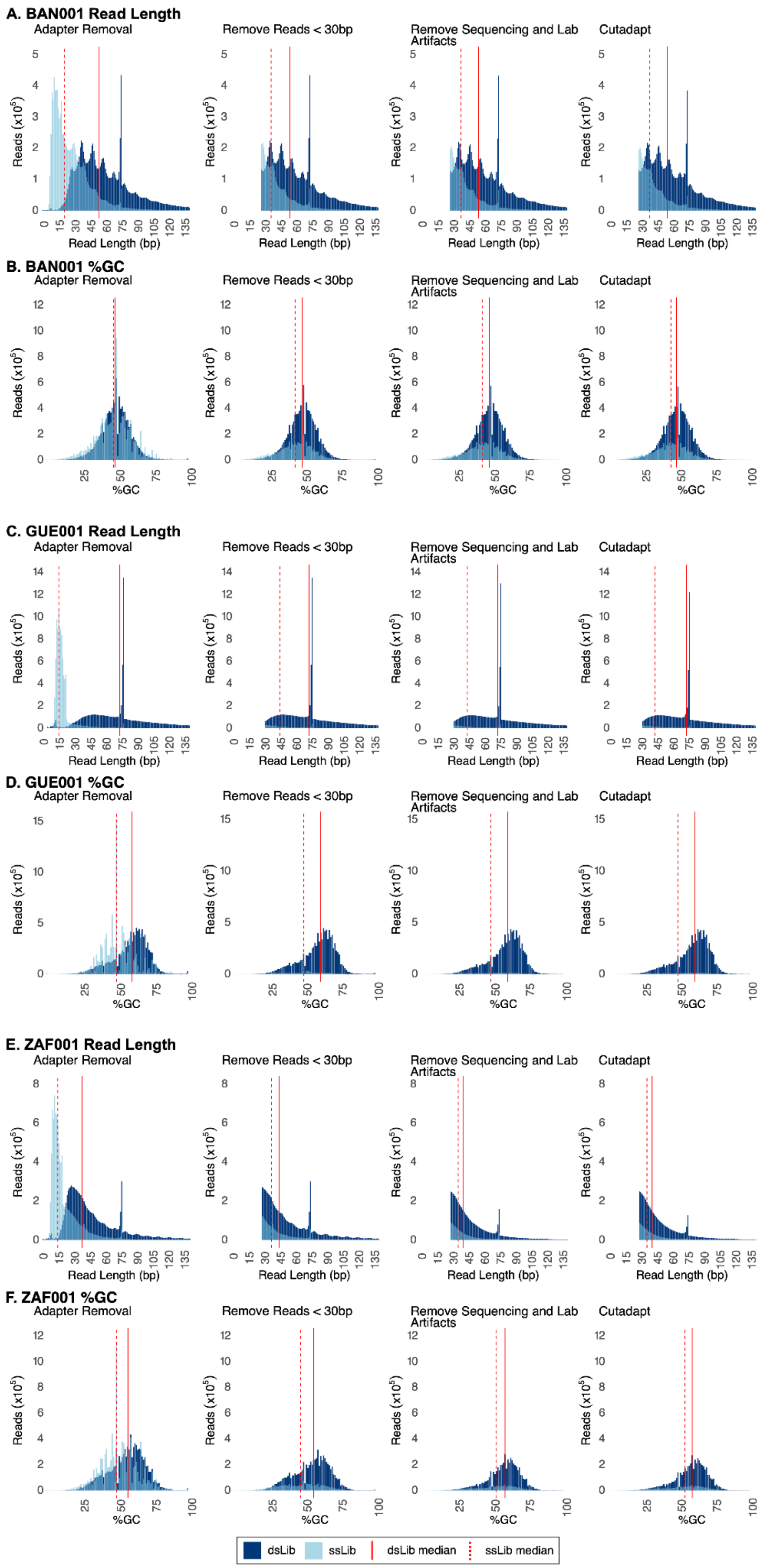

Supplemental Figure 9. Read length and GC content throughout processing steps for poorly preserved samples BAN, GUE, and ZAF.

Histograms showing the distribution of read length (bp) (**A,C,E**) and GC content (%) (**B,D,F**) for NPre BAN001 (**A,B**), GUE001 (**C,D**), and ZAF001 (**E,F**) throughout the main read processing steps: adapter removal and merging, removing reads less than 30bp, removing sequencing and laboratory artifacts, and additional adapter removal with Cutadapt. The solid red line indicates the dsLib median while the dashed line indicates the ssLib median.

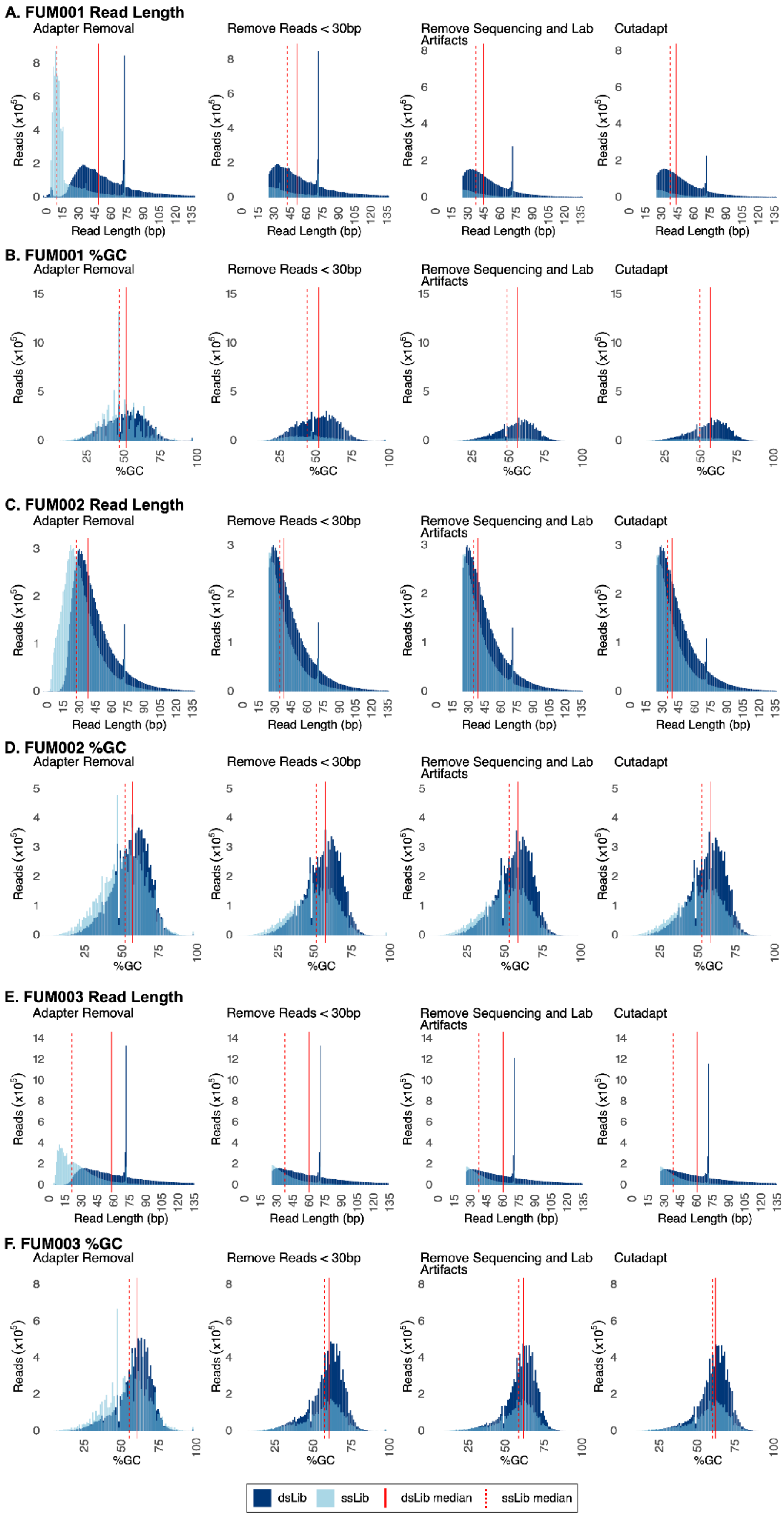

Supplemental Figure 10. Read length and GC content throughout processing steps for poorly preserved FUM samples.

Histograms showing the distribution of read length (bp) (**A,C,E**) and GC content (%) (**B,D,F**) for NPre FUM001 (**A,B**), FUM002 (**C,D**), and FUM003 (**E,F**) throughout the main read processing steps: adapter removal and merging, removing reads less than 30bp, removing sequencing and laboratory artifacts, and additional adapter removal with Cutadapt. The solid red line indicates the dsLib median while the dashed line indicates the ssLib median.

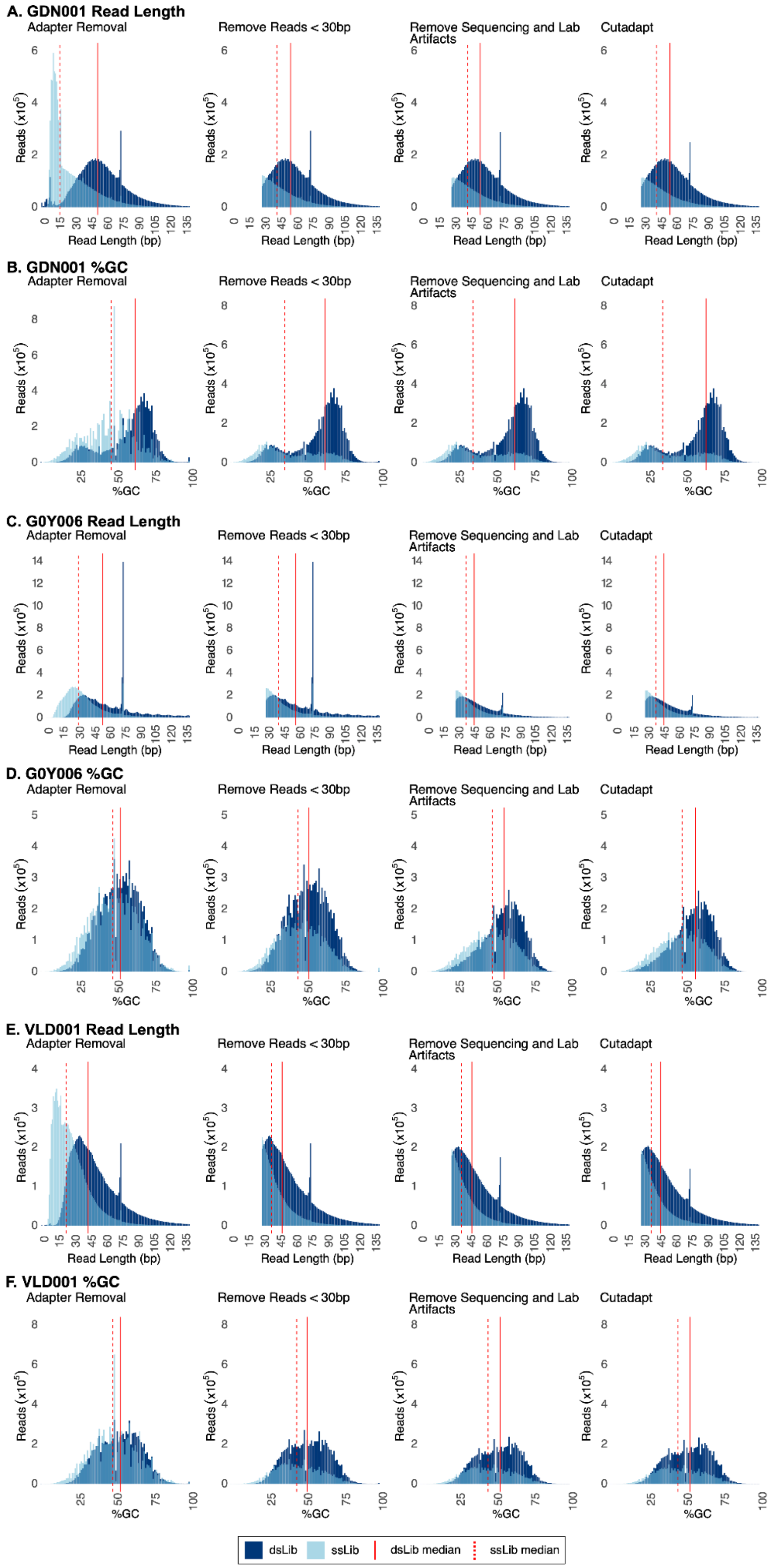

Supplemental Figure 11. Read length and GC content throughout processing steps for GDN, GOY, and VLD.

Histograms showing the distribution of read length (bp) (**A,C,E**) and GC content (%) (**B,D,F**) for NPre GDN001 (**A,B**), GOY006 (**C,D**), and VLD001 (**E,F**) throughout the main read processing steps: adapter removal and merging, removing reads less than 30bp, removing sequencing and laboratory artifacts, and additional adapter removal with Cutadapt. The solid red line indicates the dsLib median while the dashed line indicates the ssLib median.

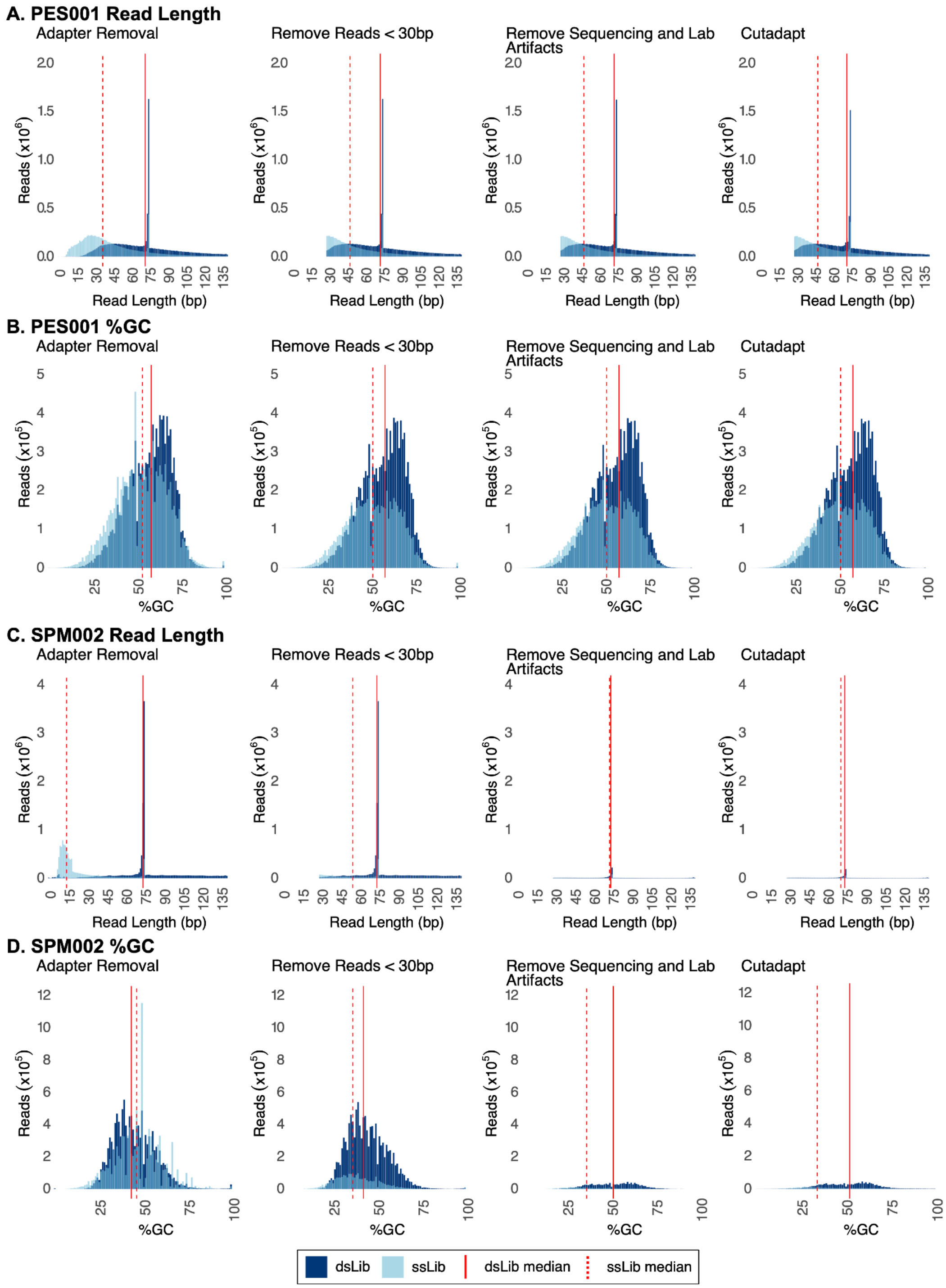

Supplemental Figure 12. PES and SPM read length and GC content throughout processing steps.

Histograms showing the distribution of read length (bp) (**A,C**) and GC content (%) (**B,D**) for NPre PES001 (**A,B**) and SPM002 (**C,D**) throughout the main read processing steps: adapter removal and merging, removing reads less than 30bp, removing sequencing and laboratory artifacts, and additional adapter removal with Cutadapt. The solid red line indicates the dsLib median while the dashed line indicates the ssLib median.

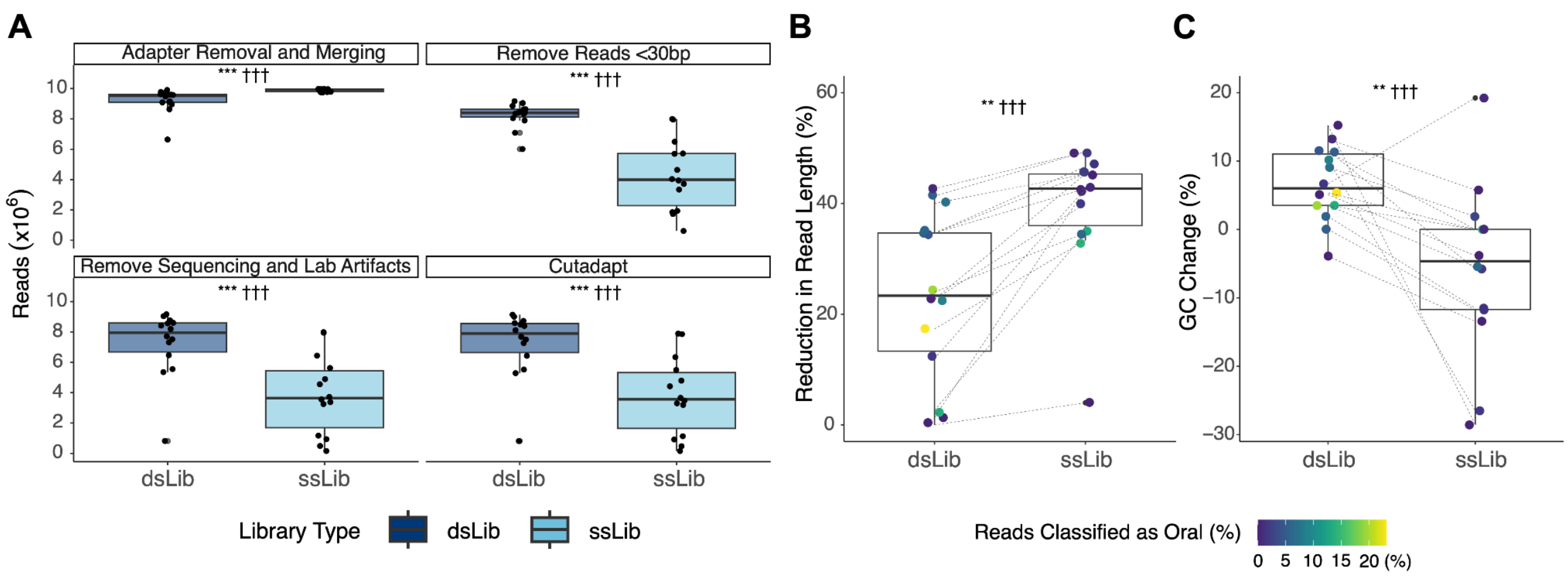

Supplemental Figure 13. Read retention and processing outcomes in *nf-core/eager* after subsampling to 10 million reads (NPre dataset).

A. Total number of reads of both dsLibs (dark blue) and ssLibs (light blue) throughout the main read processing steps (adapter removal and merging, removing reads less than 30 bp, removing sequencing and lab artifacts, and running Cutadpt to remove persisting adapter sequences). **B.** Percent reduction in read length after read processing (as compared to the initial reads sequenced at 75 bp). **C.** Percent of change in GC content after processing. Points **(B-C)** are colored by the percentage of reads classified as oral and libraries from the same sample are connected by a hashed grey line. Wilcoxon signed-rank tests were used for paired comparisons. Significance codes: ** $p < 0.01$; *** $p < 0.001$. Effect sizes (rank-biserial r): ††† large (≥0.50).

3.5 Oral microbiome–focused diversity and compositional analyses

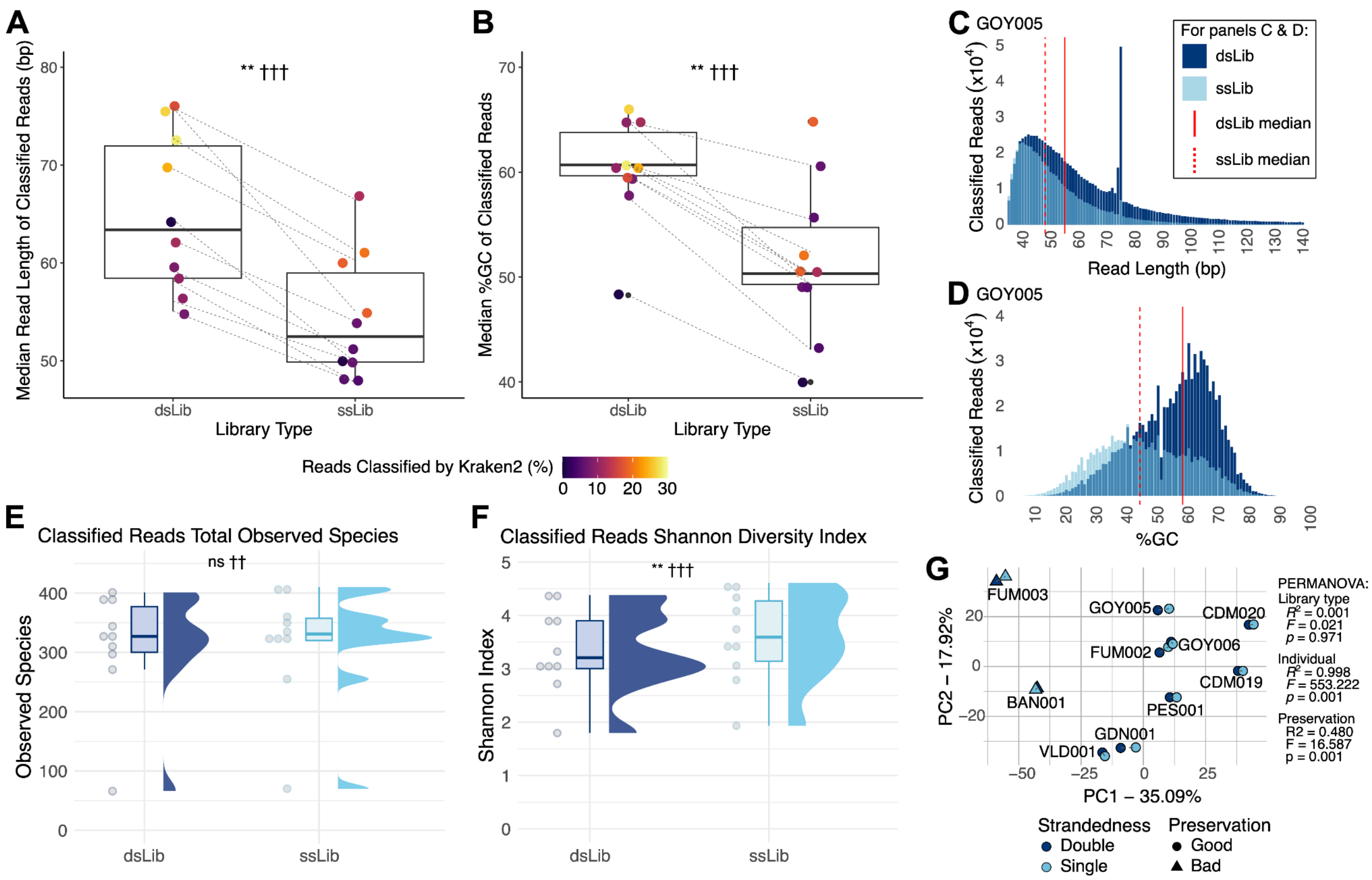

Supplemental Figure 14. Sequencing read characteristics and taxonomic diversity of samples profiled with Kraken2 after subsampling to 5M reads (NPost).

A. Median read length (bp) of reads from the NPost dataset classified by Kraken2. Points are colored by the percentage of the subsampled reads that were classified to the species level. **B.** Median GC content (%) of reads classified by Kraken2. **C.** Read length distribution of classified NPost GOY005 taxonomic profiling normalized libraries. The solid red line indicates the dsLib median while the dashed line indicates the ssLib median. **D.** GC content (%) distribution of classified NPost GOY005 reads. **E-F.** Count of total observed species **(E)** and Shannon diversity **(F)** in NPost libraries classified by Kraken2. **G.** Principal components analysis (PCA) of Kraken2 taxonomic profiles from the NPost dataset. Points are colored by strandedness, and the shape indicates preservation status. Libraries from the same sample are connected by a hashed grey line **(A-B, G)**. Wilcoxon signed-rank tests were used for paired comparisons. Significance codes: ns, $p \geq 0.05$; ** $p < 0.01$. Effect sizes (rank-biserial r): †† moderate (0.30–0.49), ††† large (≥0.50).

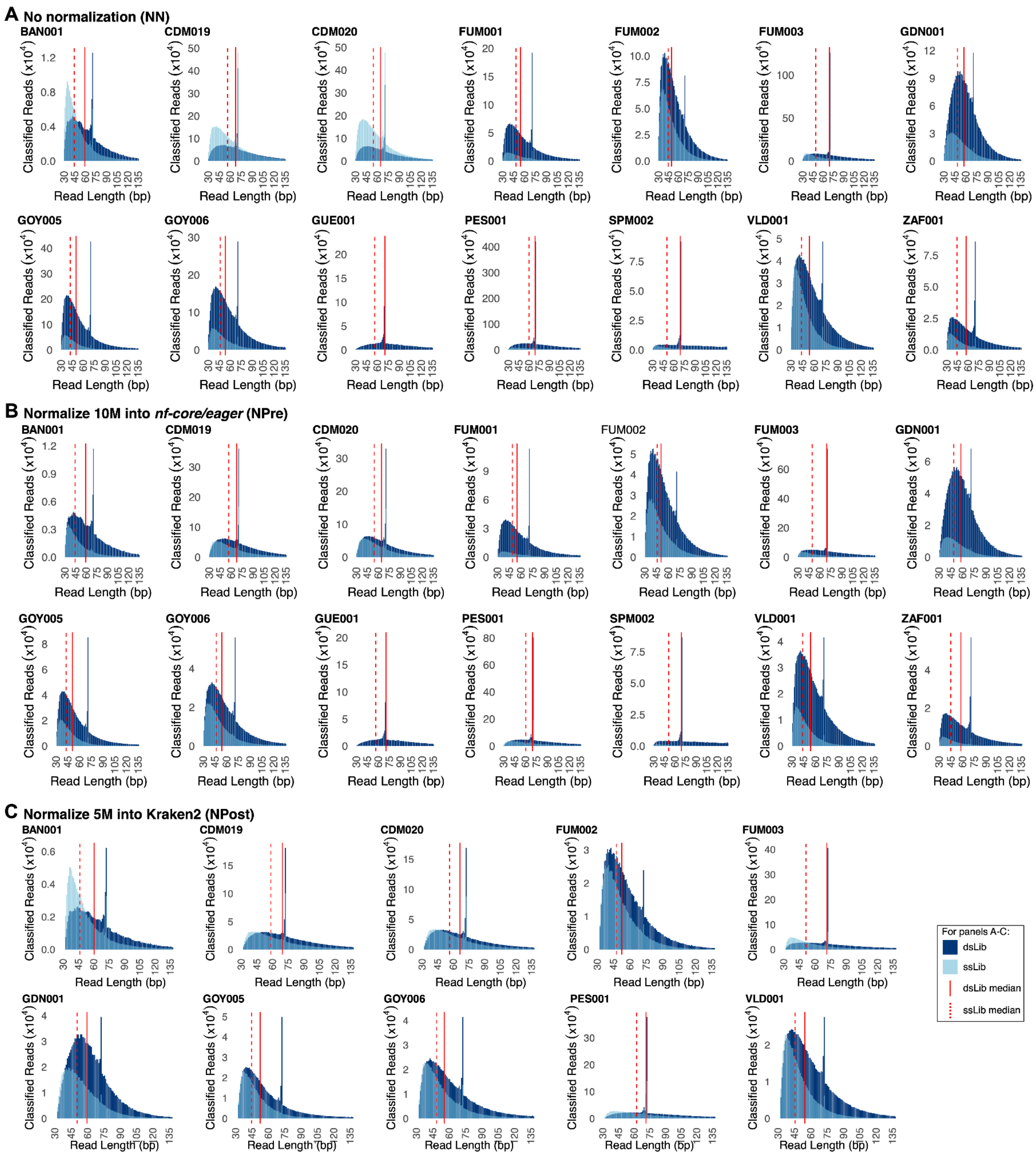

Supplemental Figure 15. Read lengths distributions of classified reads by sample.

Histograms showing the counts of reads classified by Kraken2 at each read length for the no normalization (NN) dataset **(A)**, normalizing 10 million reads prior to processing (NPre) **(B)**, and normalizing 5 million reads prior to taxonomic profiling (NPost) **(C)** divided by sample. Red solid lines indicate the dsLib median per sample while the hashed red lines indicate the ssLib median per sample.

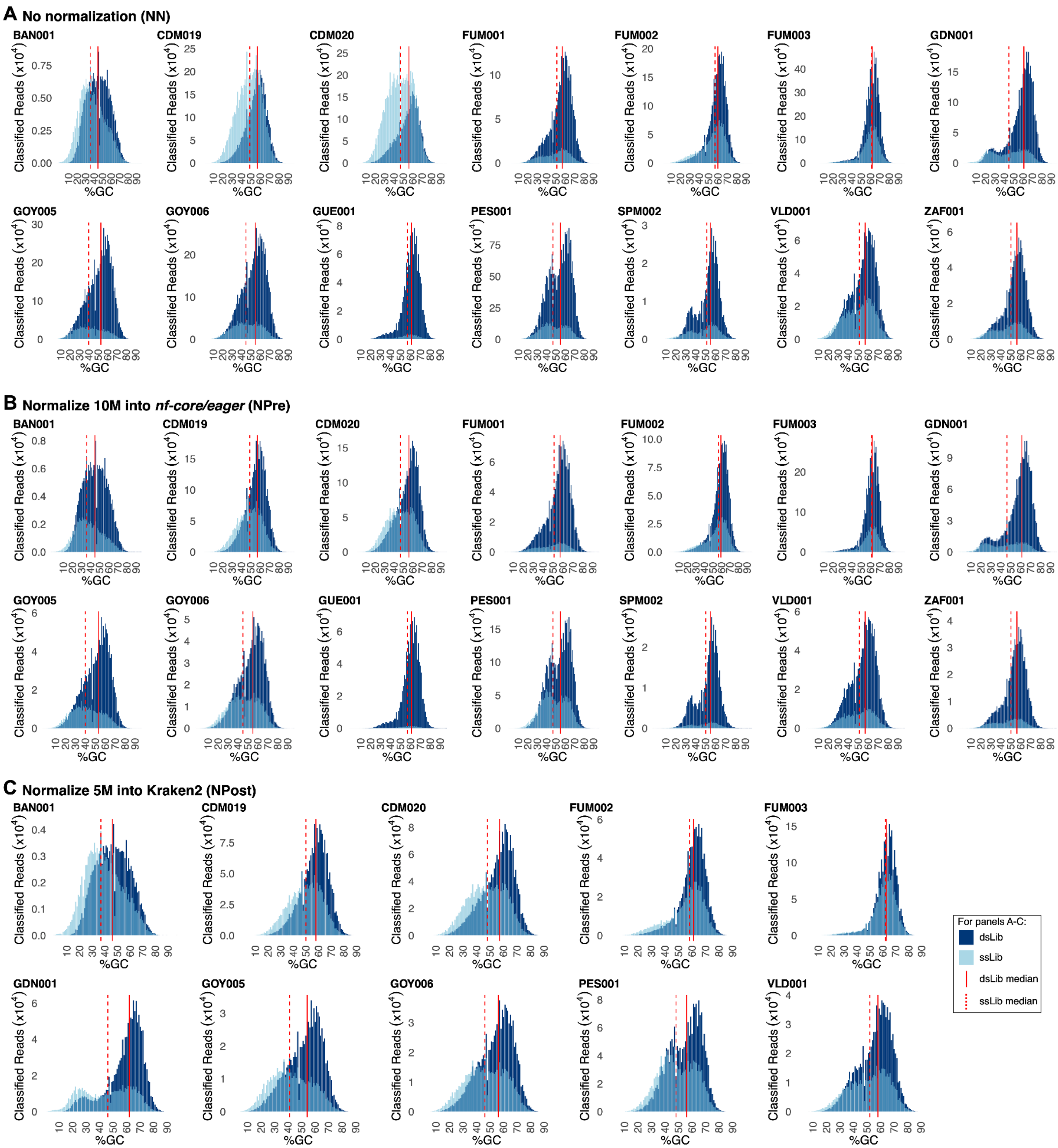

Supplemental Figure 16. GC content distributions of classified reads by sample.

Histograms showing the counts of reads classified by Kraken2 at each %GC bin for the no normalization (NN) dataset **(A)**, normalizing 10 million reads prior to processing (NPre) **(B)**, and normalizing 5 million reads prior to taxonomic profiling (NPost) **(C)** divided by sample. Red solid lines indicate the dsLib median per sample while the hashed red lines indicate the ssLib median per sample.

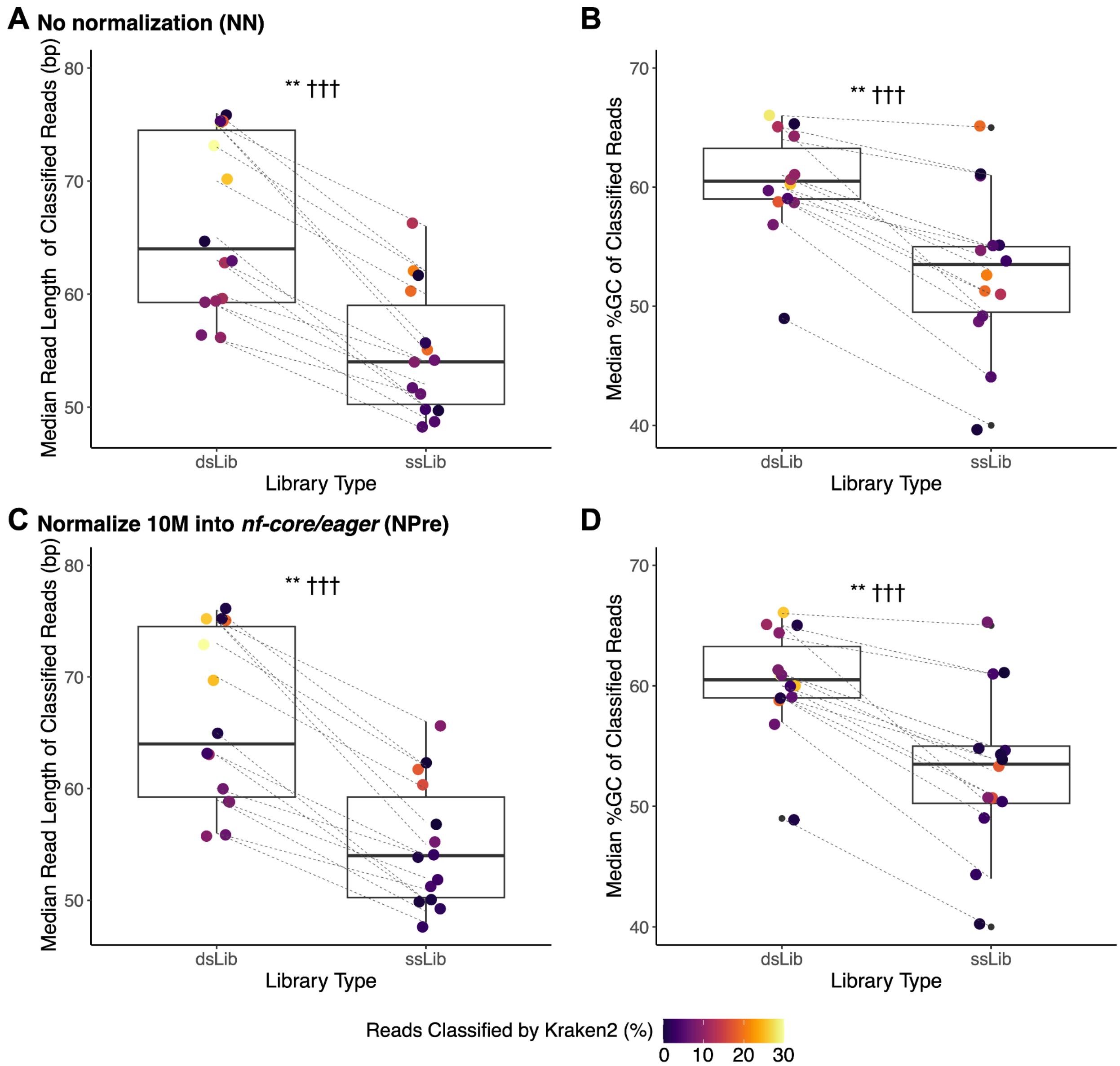

Supplemental Figure 17. Median read length and %GC of classified Kraken2 reads.

Boxplots showing the median read length (bp) **(A, C)** and %GC **(B, D)** of the total classified reads from each sample for the no normalization (NN) dataset **(A, B)** and the NPre dataset subsampling to 10 million reads prior to read processing **(C, D)**. Grey hashed lines connect the dsLib and ssLib of the same individual. Points are colored by the percent of processed reads (post *nf-core/eager* and Cutadpt) classified by Kraken2. Wilcoxon signed-rank tests were used for paired comparisons. Significance codes: ** $p < 0.01$. Effect sizes (rank-biserial r): ††† large (≥0.50).

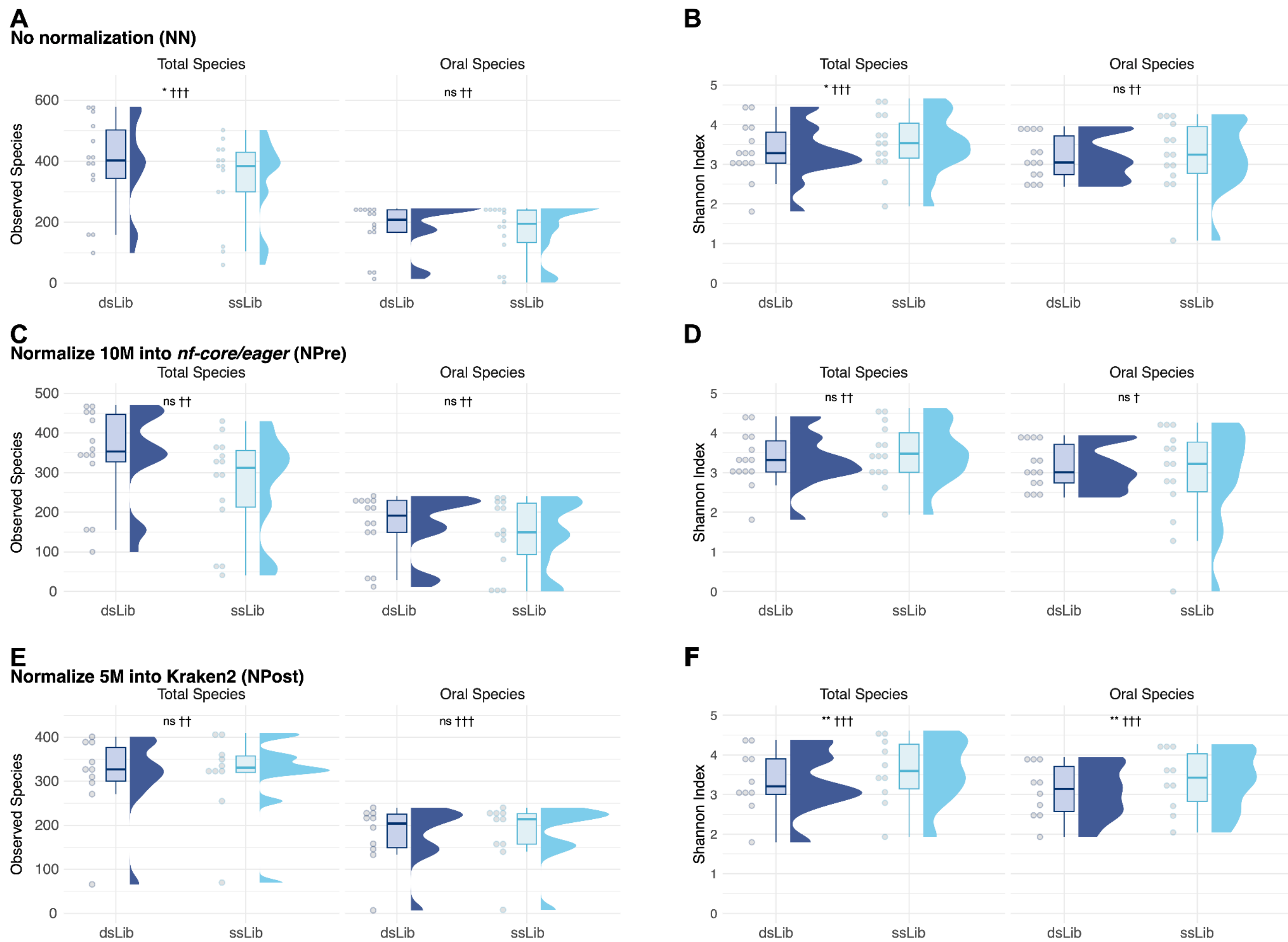

Supplemental Figure 18. Alpha-diversity analyses for both total classified reads and oral reads.

Raincloud plots showing both the total observed species count **(A, C, E)** and Shannon diversity index **(B, D, F)** for each normalization dataset: no normalization (NN) **(A, B)**, normalizing to 10 million reads prior to processing (NPre) **(C, D)**, and normalizing to 5 million reads prior to taxonomic profiling (NPost) **(E, F)**. For each plot, the left panel shows the alpha-diversity metric for all reads classified by Kraken2, and the right panel shows the alpha-diversity metric for only reads assigned to oral taxa. Wilcoxon signed-rank tests were used for paired comparisons. Significance codes: ns, $p \geq 0.05$; *$p < 0.05$; ** $p < 0.01$. Effect sizes (rank-biserial r): † small (<0.30), †† moderate (0.30–0.49), ††† large (≥0.50).

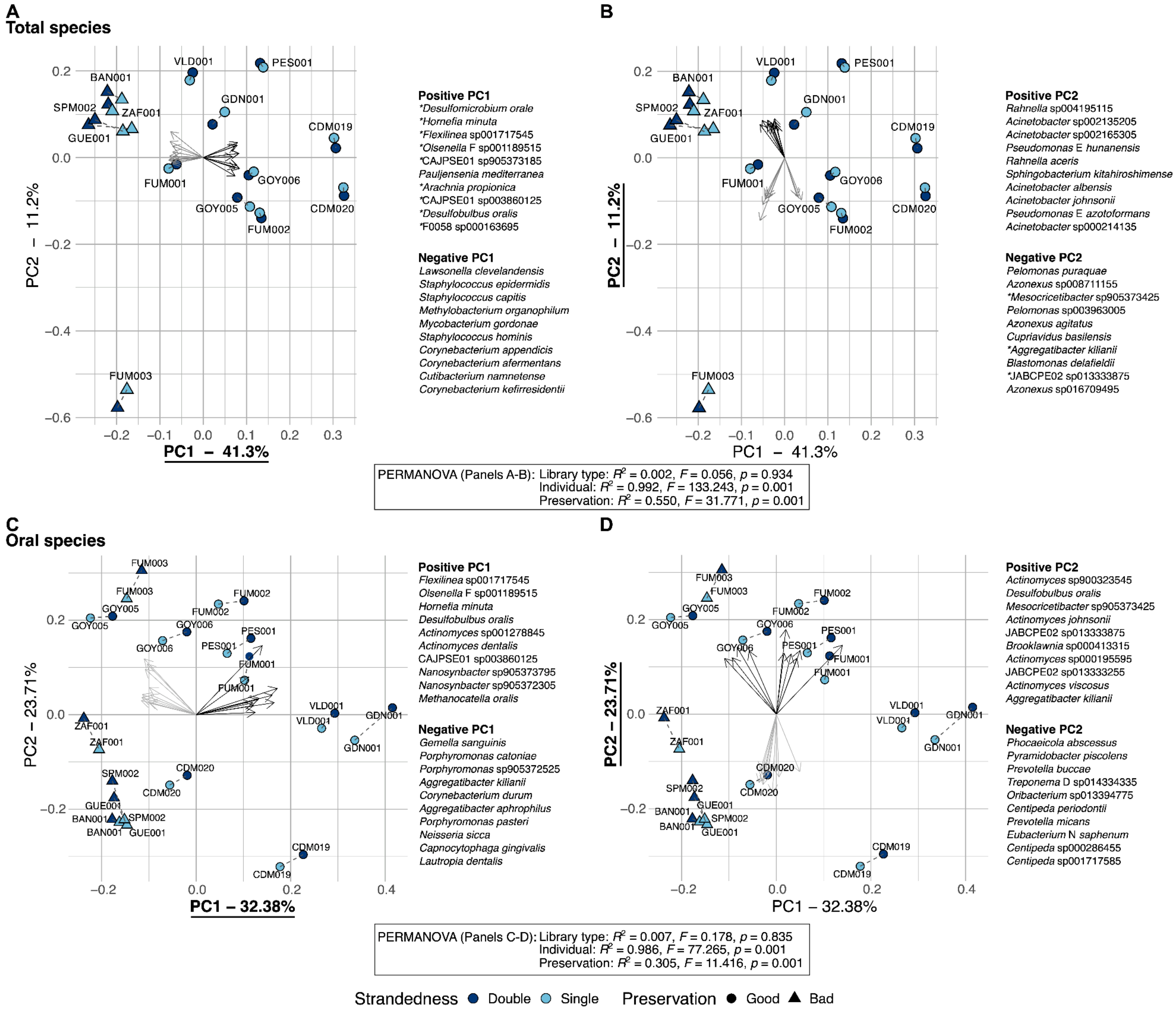

Supplemental Figure 19. Drivers of PCA structure for dataset normalizing 10 million reads prior to processing steps.

Principal component analysis (PCA) of samples based on NPre Kraken2 species tables after normalizing to 10 million reads prior to processing via *nf-core/eager* for both total species classified **(A, B)** and reads attributed only to oral species **(C, D)**. **A, C.** Arrows indicate species loadings, with the direction and magnitude reflecting their contribution to PC1. Species with the strongest positive and negative loadings on PC1 are listed. **B, D.** PCA highlighting species driving separation along PC2, with associated species loadings. Libraries from the same sample are connected by a hashed grey line. Asterisks next to species names in the total species **(A, B)** PCAs indicate oral associated taxa.

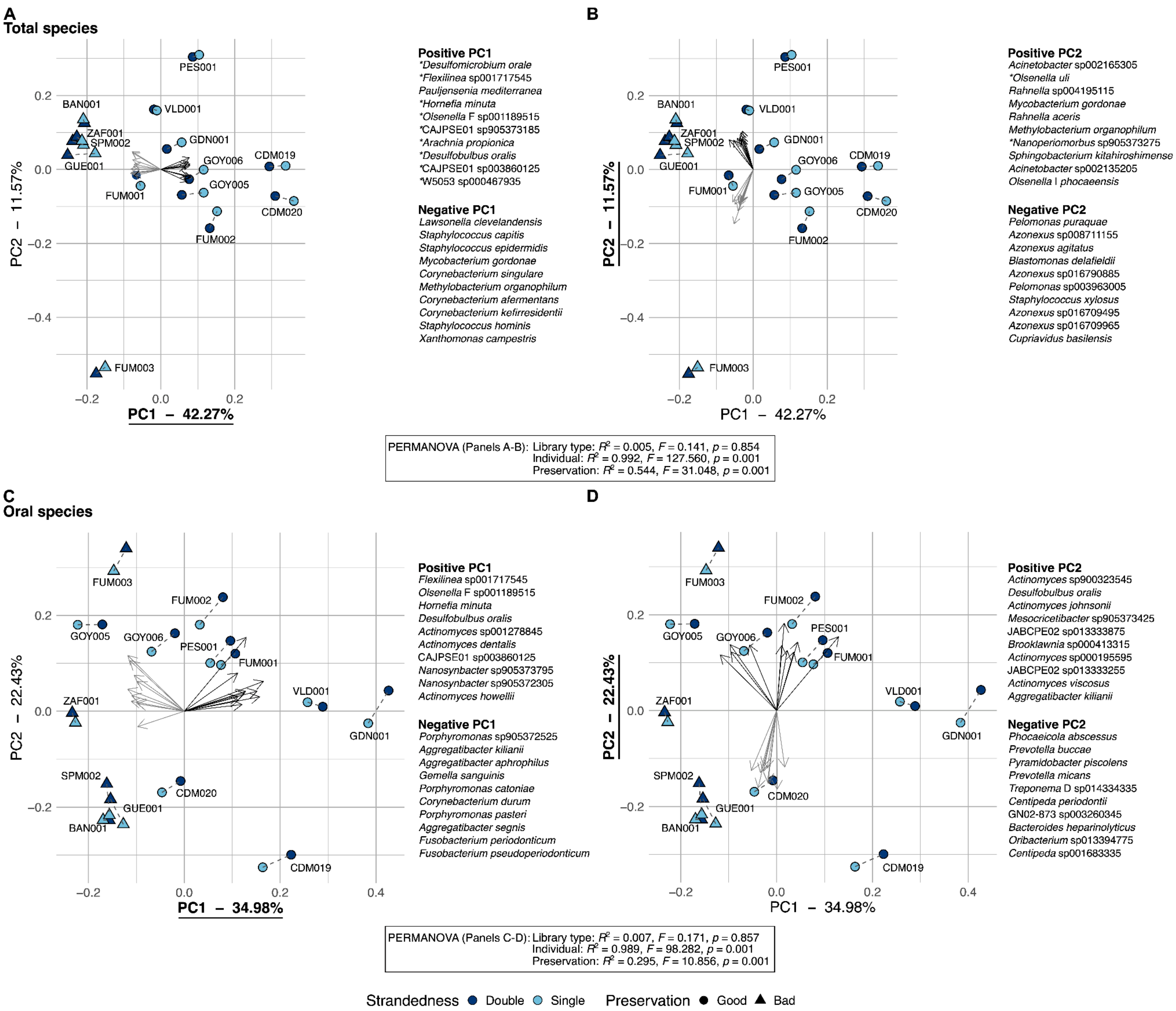

Supplemental Figure 20. Drivers of PCA structure for the no normalization dataset.

Principal component analysis (PCA) of samples based on NN Kraken2 species tables after not normalizing prior to both processing and taxonomic profiling for total species **(A, B),** oral species **(C, D)**, and oral species of only well-preserved samples (no BAN001, FUM003, GUE001, SPM001, or ZAF001). **A, C, E.** Arrows indicate species loadings, with the direction and magnitude reflecting their contribution to PC1. Species with the strongest positive and negative loadings on PC1 are listed. **B, D, F.** PCA highlighting species driving separation along PC2, with associated species loadings. Libraries from the same sample are connected by a hashed grey line. Asterisks next to species names in the total species **(A, B)** PCAs indicate oral associated taxa.

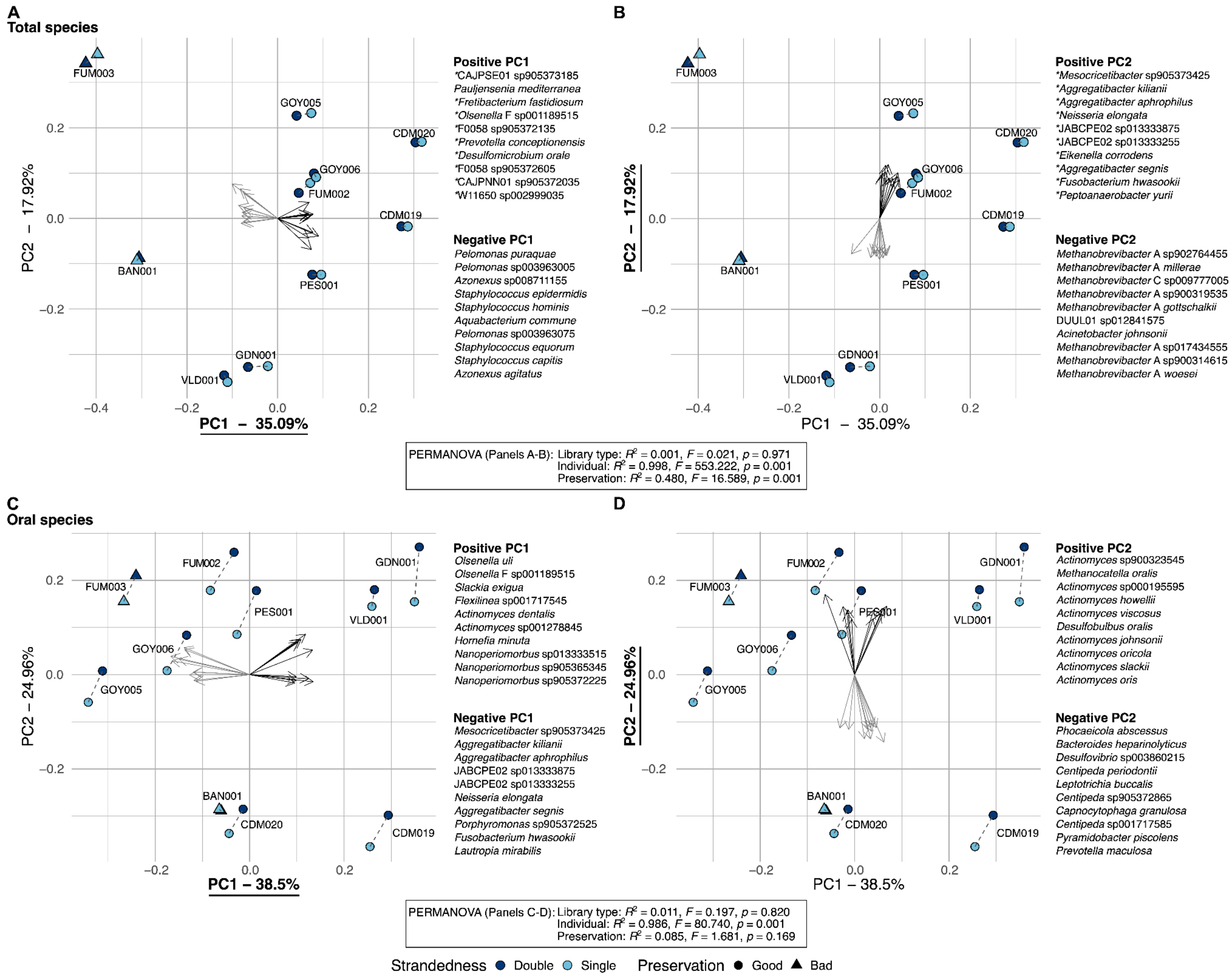

Supplemental Figure 21. Drivers of PCA structure for dataset normalizing 5 million reads into Kraken2 (NPost).

Principal component analysis (PCA) of samples based on NPost Kraken2 species tables after normalizing to 5 million reads for both total species **(A, B)** and oral species **(C, D)**. **A, C.** Arrows indicate species loadings, with the direction and magnitude reflecting their contribution to PC1. Species with the strongest positive and negative loadings on PC1 are listed. **B, D.** PCA highlighting species driving separation along PC2, with associated species loadings. Libraries from the same sample are connected by a hashed grey line. Asterisks next to species names in the total species **(A, B)** PCAs indicate oral associated taxa.

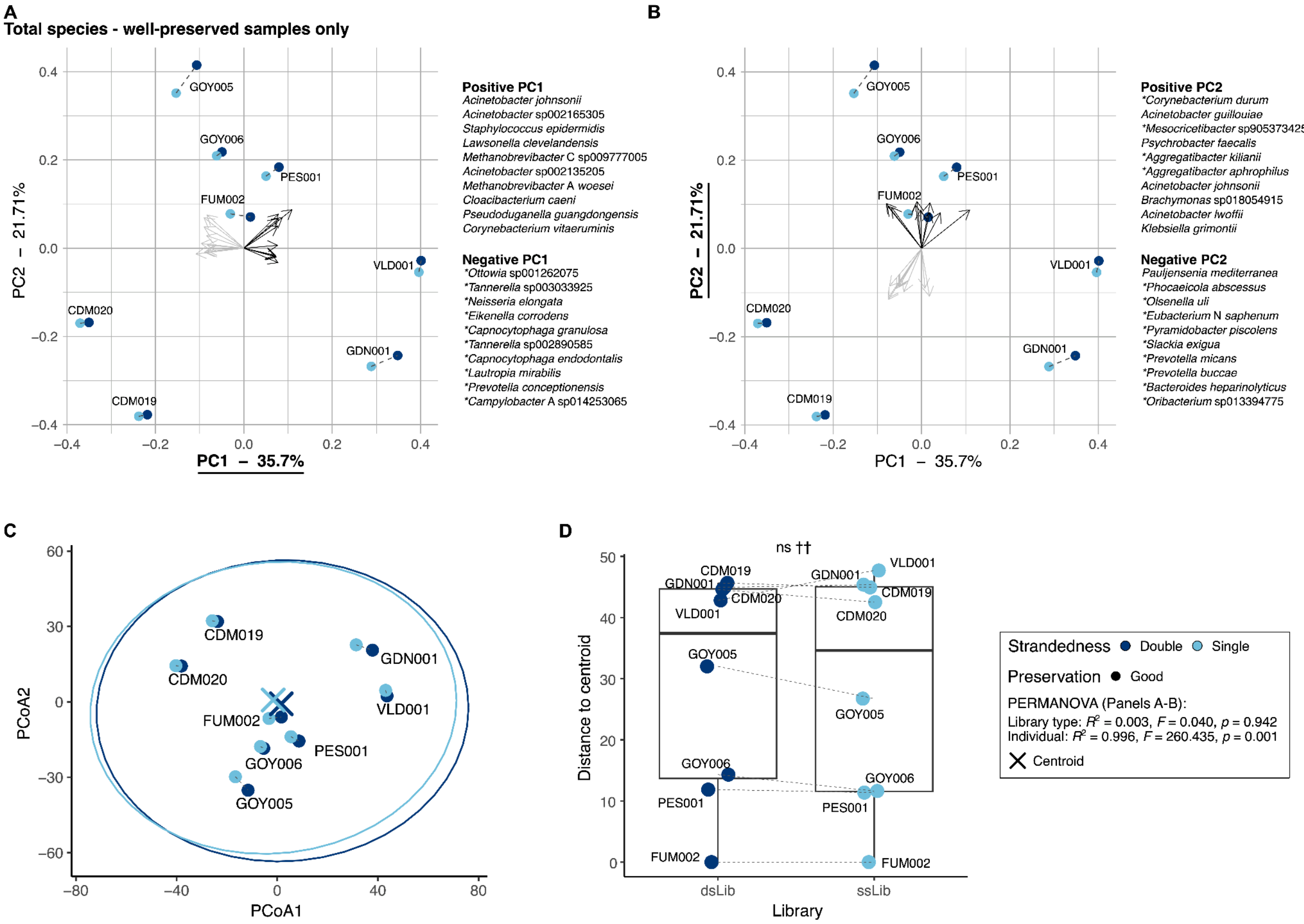

Supplemental Figure 22. Total species beta diversity and dispersion of well-preserved samples from the NPost dataset (subsampled to 5M reads prior to taxonomic profiling).

A-B. Principal component analysis (PCA) of samples based on Kraken2 total species tables after filtering for only well-preserved samples in the NPost dataset. **A.** Arrows indicate species loadings, with the direction and magnitude reflecting their contribution to PC1. Species with the strongest positive and negative loadings on PC1 are listed. **B.** PCA highlighting species driving separation along PC2, with associated species loadings. Libraries from the same sample are connected by a hashed grey line. **C.** Principal coordinates analysis (PCoA) of Euclidean distances showing sample clustering, with ellipses representing group dispersion and crosses indicating group centroids. **D.** Distances to group centroids (**C**) (with vegan function `betadisper` (Oksanen et al., 2025)) for each sample by library type, with paired samples connected by dashed lines. Wilcoxon signed-rank tests were used for paired comparisons. Significance codes: ns, $p \geq 0.05$. Effect size: †† moderate (0.30–0.49).

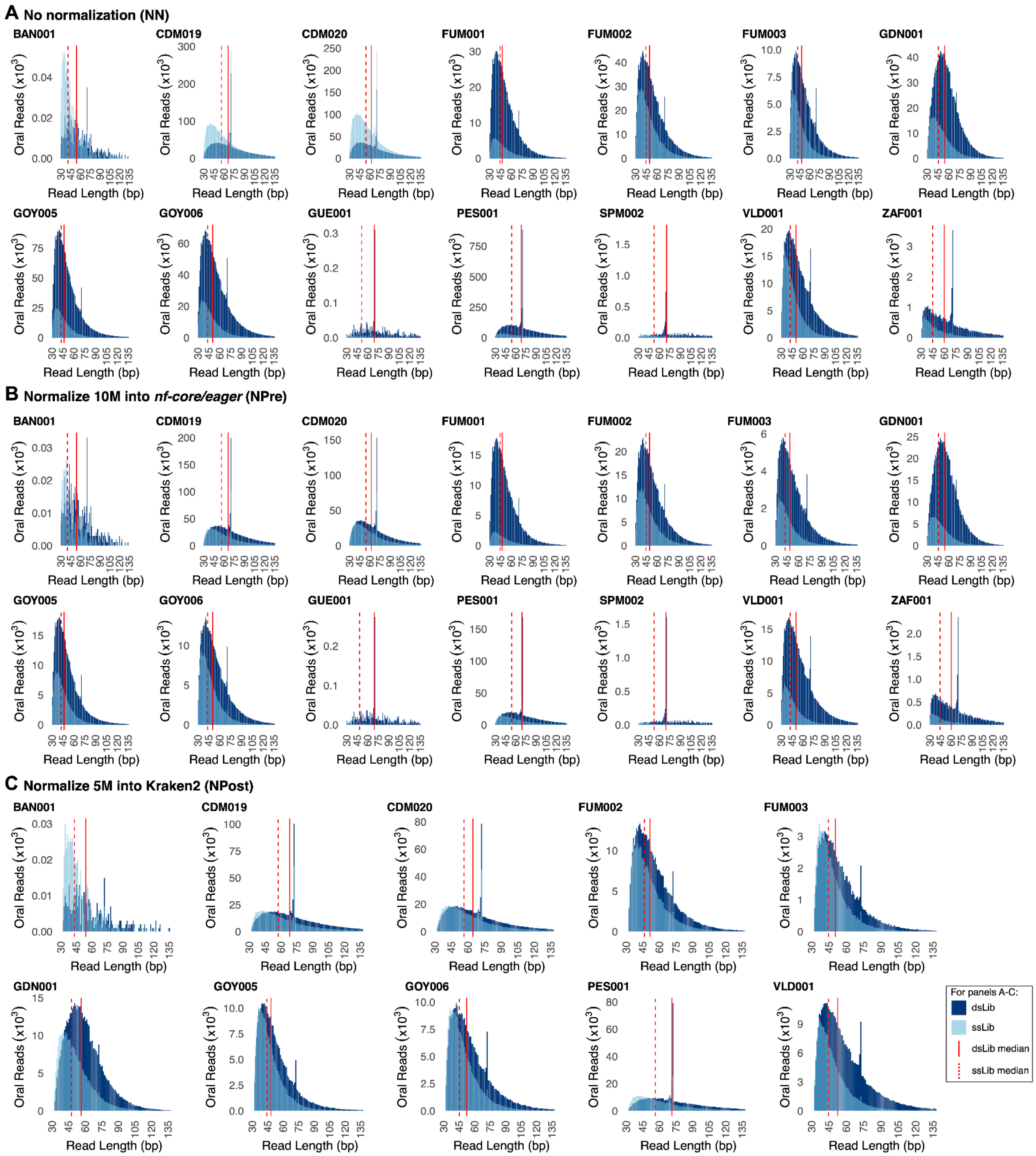

Supplemental Figure 23. Read lengths distributions of oral reads by sample.

Histograms showing the counts of oral reads at each read length for the no normalization (NN) dataset **(A)**, normalizing 10 million reads prior to processing (NPre) **(B)**, and normalizing 5 million reads prior to taxonomic profiling (NPost) **(C)** divided by sample. Red solid lines indicate the dsLib median per sample while the hashed red lines indicate the ssLib median per sample.

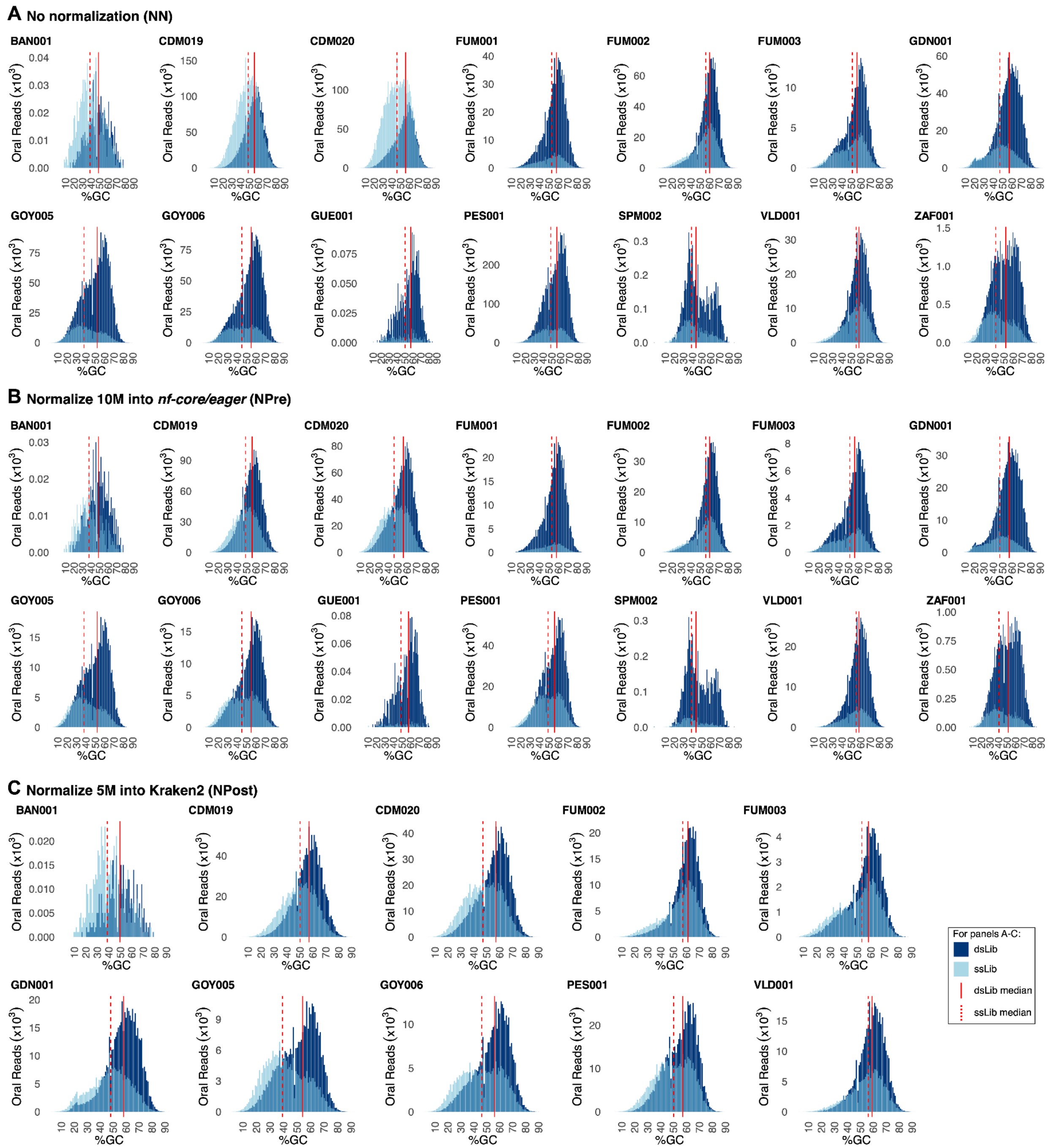

Supplemental Figure 24. GC content distributions of oral reads by sample.

Histograms showing the counts of oral reads at each %GC bin for the no normalization (NN) dataset **(A)**, normalizing 10 million reads prior to processing (NPre) **(B)**, and normalizing 5 million reads prior to taxonomic profiling (NPost) **(C)** divided by sample. Red solid lines indicate the dsLib median per sample while the hashed red lines indicate the ssLib median per sample.

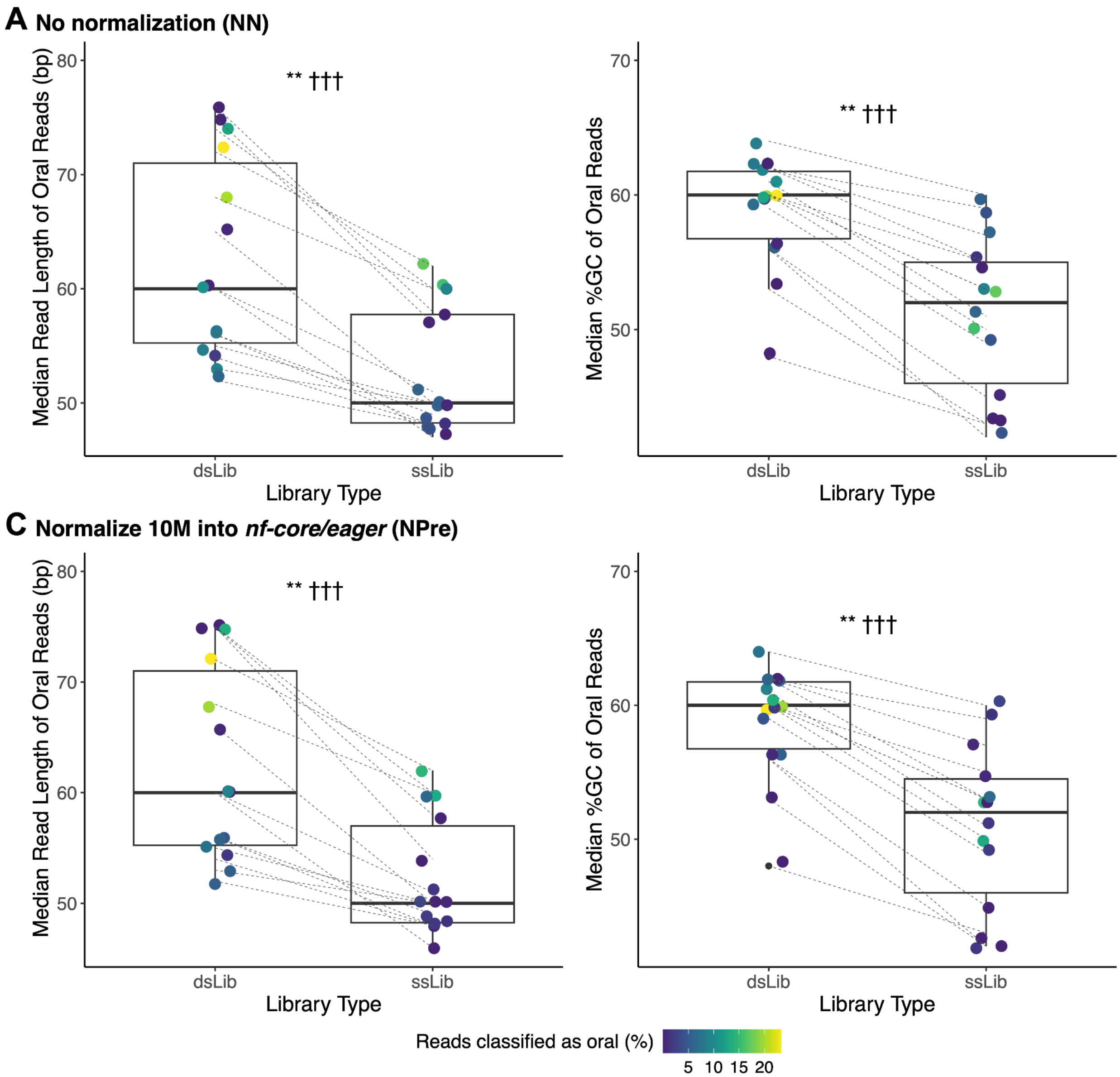

Supplemental Figure 25. Median read length and %GC of oral reads.

Boxplots showing the median read length (bp) **(A, C)** and %GC **(B, D)** of the reads attributed to oral taxa from each sample for the no normalization (NN) dataset **(A, B)** and the NPre dataset subsampling to 10 million reads prior to read processing **(C, D)**. Grey hashed lines connect the dsLib and ssLib of the same individual. Points are colored by the percent of processed reads (post *nf-core/eager* and Cutadpt) determined to be oral. Wilcoxon signed-rank tests were used for paired comparisons. Significance codes: ** $p < 0.01$. Effect sizes (rank-biserial r): ††† large (≥0.50).

3.6 Differences in taxonomic identification based on read length

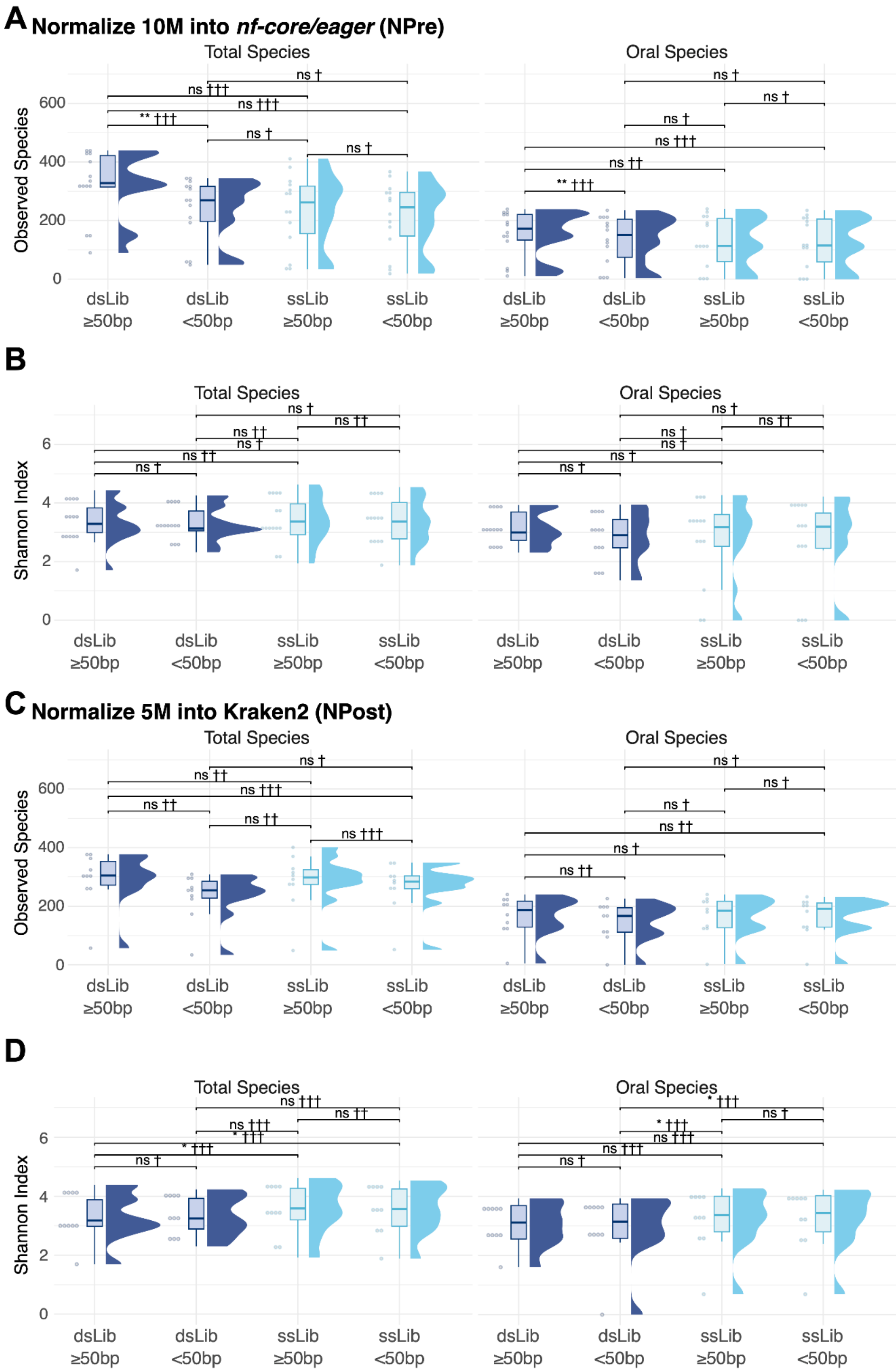

Supplemental Figure 26. Alpha-diversity analyses for read stratification datasets.

Raincloud plots showing both the total observed species count **(A, C)** and Shannon diversity index **(B, D)** for the NPre and NPost dataset read length stratification (<50 bp versus ≥50 bp): normalizing to 10 million reads prior to processing **(A, B)**, and normalizing to 5 million reads prior to taxonomic profiling **(C, D)**. For each plot, the left panel shows the alpha-diversity metric for all reads classified by Kraken2, and the right panel shows the alpha-diversity metric for only reads assigned to oral taxa. Wilcoxon signed-rank tests were used for paired comparisons. Significance codes: ns, $p \geq 0.05$; *$p < 0.05$; ** $p < 0.01$. Effect sizes (rank-biserial r): † small (<0.30), †† moderate (0.30–0.49), ††† large (≥0.50).

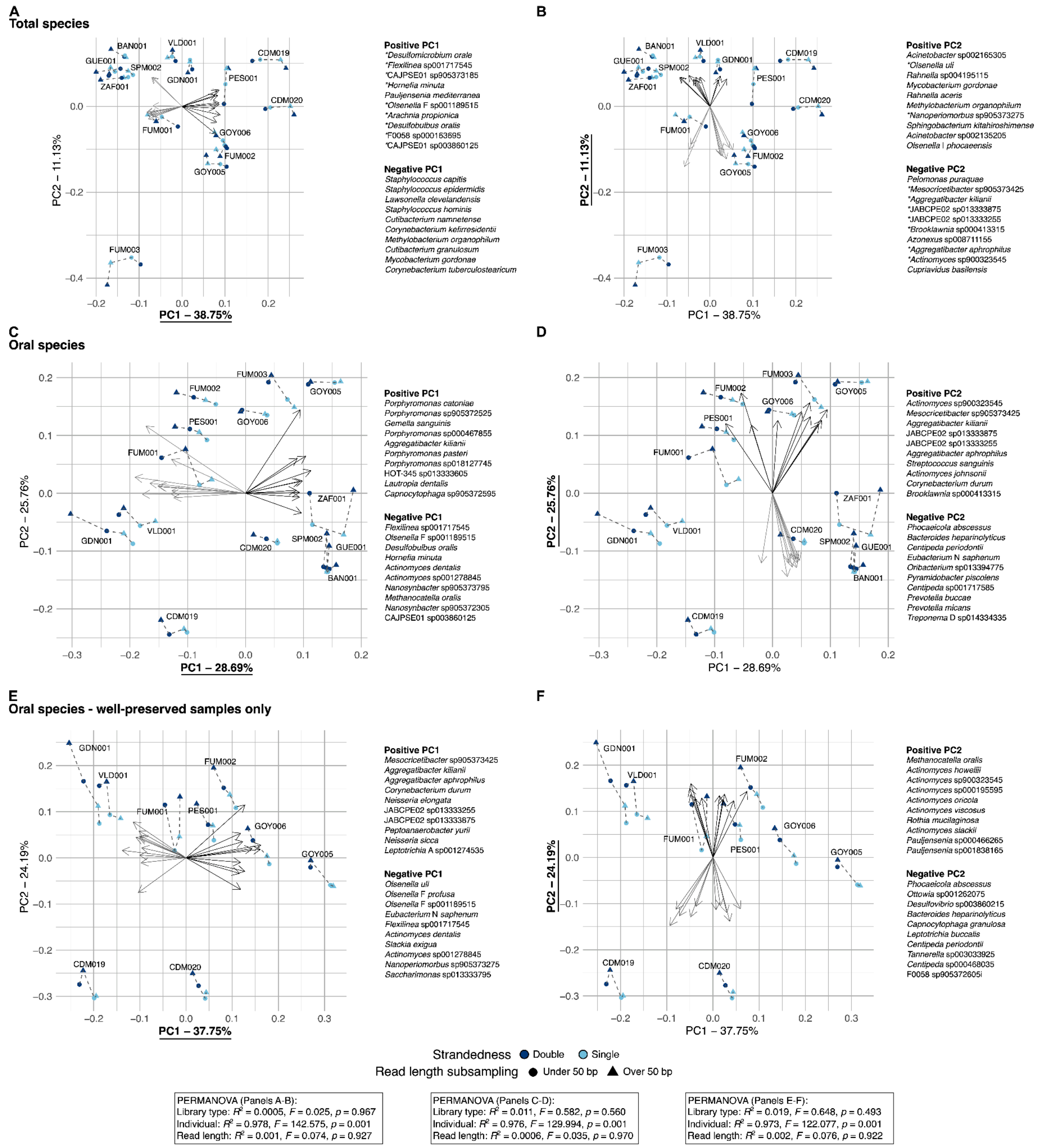

Supplemental Figure 27. Drivers of PCA structure for dataset normalizing 10 million reads prior to processing steps and stratifying by read length.

Principal component analysis (PCA) of samples based on NPre Kraken2 species tables after normalizing to 10 million reads prior to processing via *nf-core/eager* and stratifying by read length (<50 bp versus ≥50 bp) for total species **(A, B),** oral species **(C, D)**, and oral species of only well-preserved samples (no BAN001, FUM003, GUE001, SPM001, or ZAF001). **A, C, E.** Arrows indicate species loadings, with the direction and magnitude reflecting their contribution to PC1. Species with the strongest positive and negative loadings on PC1 are listed. **B, D, F.** PCA highlighting species driving separation along PC2, with associated species loadings. Libraries from the same sample are connected by a hashed grey line. Asterisks next to species names in the total species **(A, B)** PCAs indicate oral associated taxa.

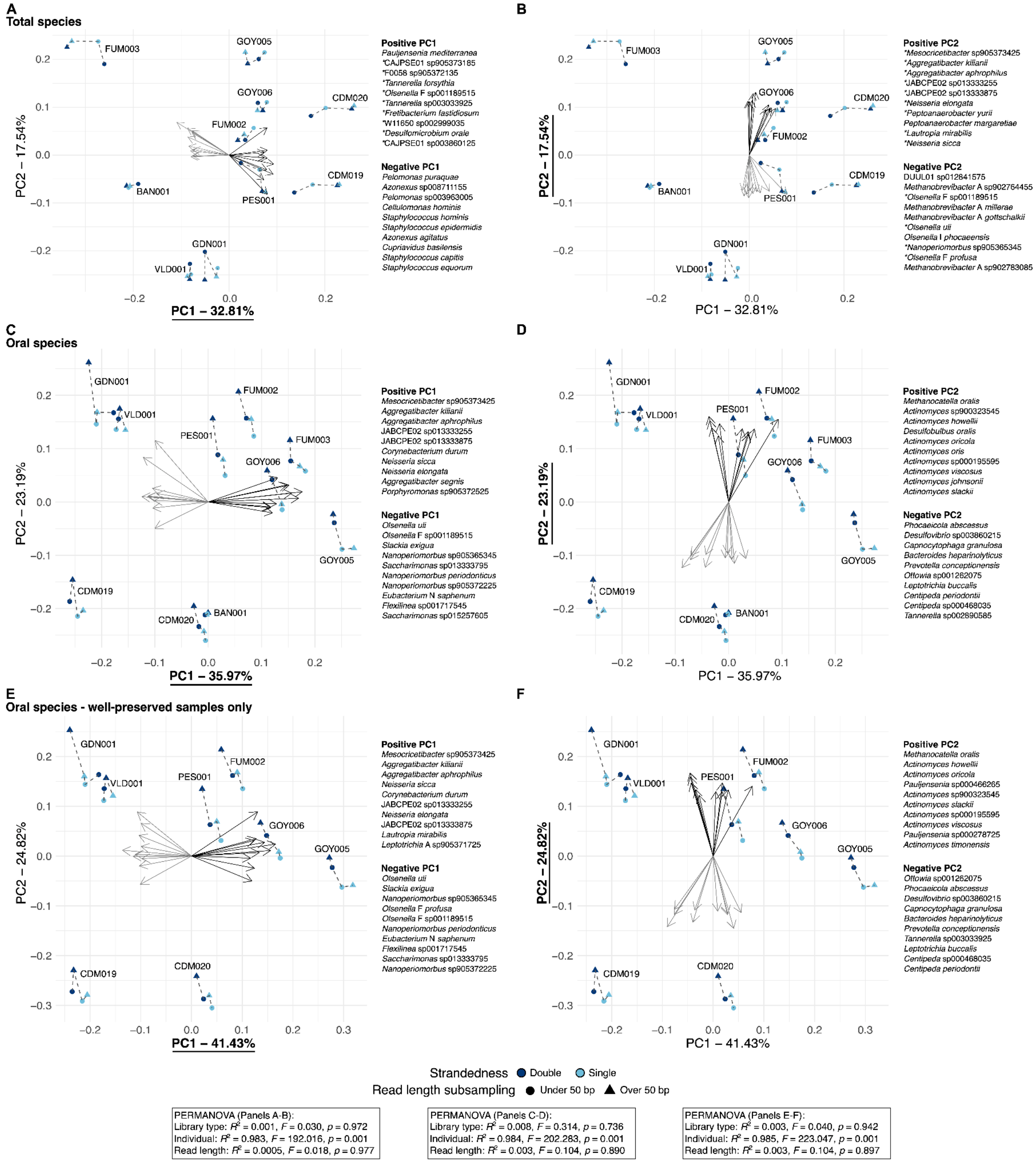

Supplemental Figure 28. Drivers of PCA structure for dataset normalizing 5 million reads into Kraken2 and stratifying by read length.

Principal component analysis (PCA) of samples based on NPost Kraken2 species tables after normalizing to 5 million reads and stratifying by read length (<50 bp versus ≥50 bp) for total species **(A, B),** oral species **(C, D)**, and oral species of only well-preserved samples (no BAN001 or FUM003). **A, C, E.** Arrows indicate species loadings, with the direction and magnitude reflecting their contribution to PC1. Species with the strongest positive and negative loadings on PC1 are listed. **B, D, F.** PCA highlighting species driving separation along PC2, with associated species loadings. Libraries from the same sample are connected by a hashed grey line. Asterisks next to species names in the total species **(A, B)** PCAs indicate oral associated taxa.

3.7 Oral species-level drivers of compositional differences between library protocols

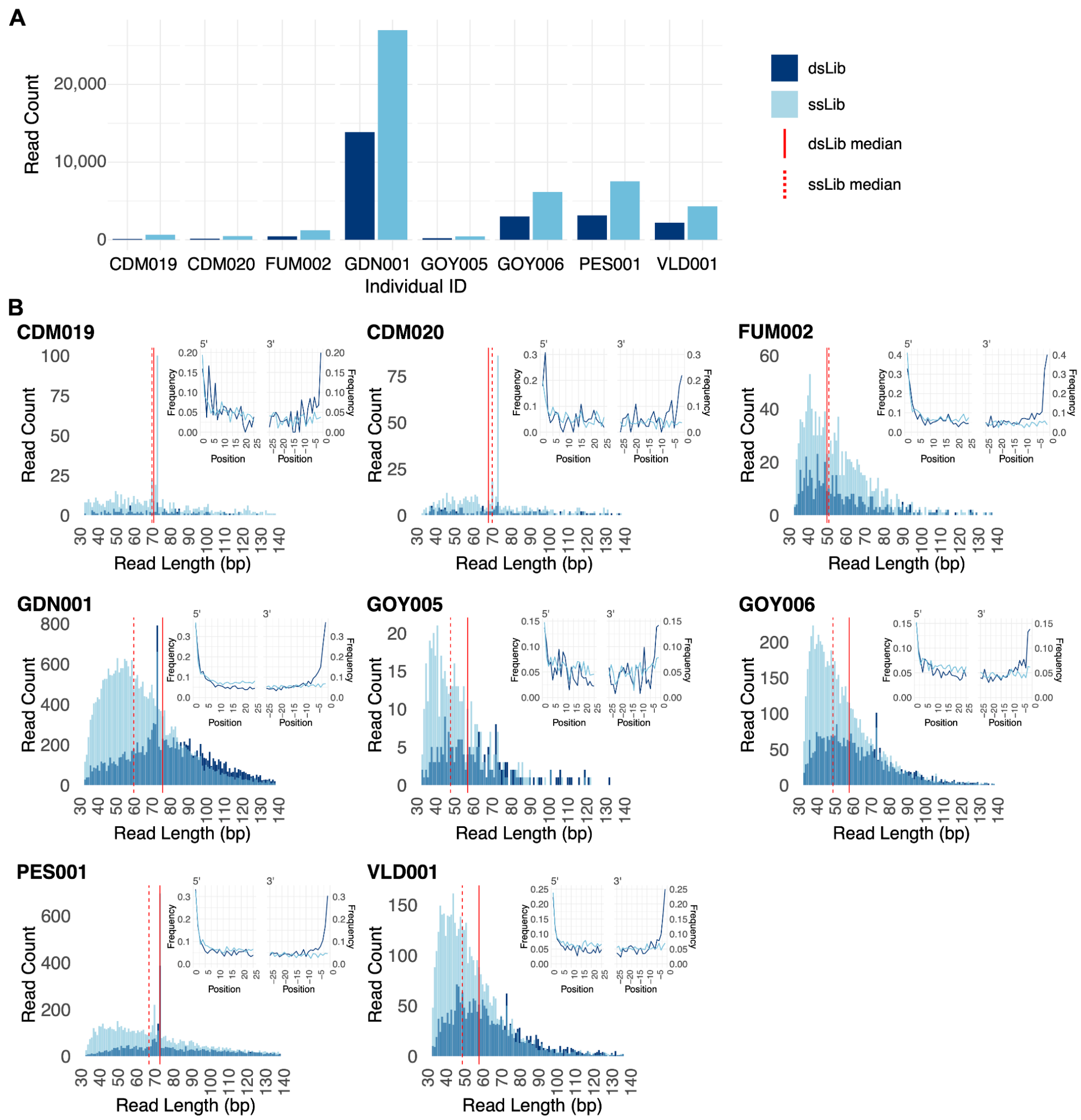

Supplemental Figure 29. ***Methanocatella oralis*** **(27.5% GC, low GC taxa) per sample read length distributions and damage.**

A. Bar plot displaying the total number of *Methanocatella oralis* reads per well-preserved library subsampled to 5 million reads prior to taxonomic profiling (NPost). Only samples with over 100 reads were included. **B.** Histograms showing the distribution of read length for all *M. oralis* reads for well-preserved samples subsampled to 5 million reads prior to taxonomic profiling. Plots are grouped by individual. The solid red line indicates the dsLib median while the dashed line indicates the ssLib median. Insets to the right of each histogram show nucleotide misincorporation patterns to assess characteristic aDNA damage. The left panel displays the frequency of cytosine deamination at the 5′ end (C→T), and the right panel shows the complementary guanine to adenine (G→A) transitions at the 3′ end. Double-stranded libraries exhibit complementary G→A substitutions at the 3′ end due to blunt-end repair during library preparation (Briggs et al., 2007). In contrast, single-stranded libraries show complementary C→T patterns at both ends because the DNA molecules are fully denatured and reconstructed without fill-in or blunt-end repair (Meyer et al., 2012). As a result, ssLibs typically display a flat profile at the 3′ end when plotting G→A transitions, since this misincorporation pattern is not expected in ssLib preparation.

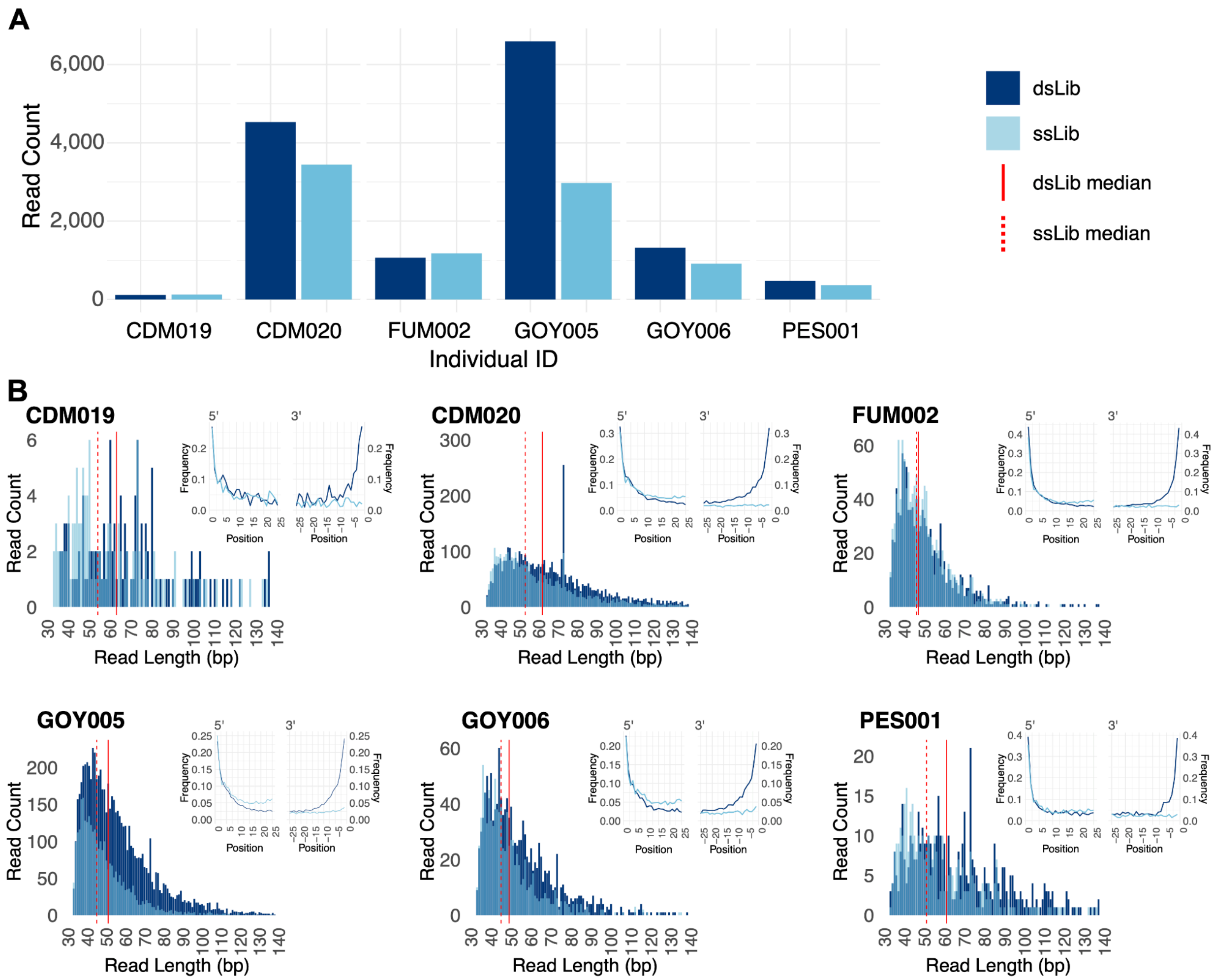

Supplemental Figure 30. *Neisseria elongata* (54% GC, mid GC taxa) per sample read length distributions and damage.

A. Bar plot displaying the total number of *Neisseria elongata* reads per well-preserved library subsampled to 5 million reads prior to taxonomic profiling (NPost). Only samples with over 100 reads were included. **B.** Histograms showing the distribution of read length for all *N. elongata* reads for well-preserved samples subsampled to 5 million reads prior to taxonomic profiling. Plots are grouped by individual. The solid red line indicates the dsLib median while the dashed line indicates the ssLib median. Insets to the right of each histogram show nucleotide misincorporation patterns to assess characteristic aDNA damage. The left panel displays the frequency of cytosine deamination at the 5′ end (C→T), and the right panel shows the complementary guanine to adenine (G→A) transitions at the 3′ end. Double-stranded libraries exhibit complementary G→A substitutions at the 3′ end due to blunt-end repair during library preparation (Briggs et al., 2007). In contrast, single-stranded libraries show complementary C→T patterns at both ends because the DNA molecules are fully denatured and reconstructed without fill-in or blunt-end repair (Meyer et al., 2012). As a result, ssLibs typically display a flat profile at the 3′ end when plotting G→A transitions, since this misincorporation pattern is not expected in ssLib preparation.

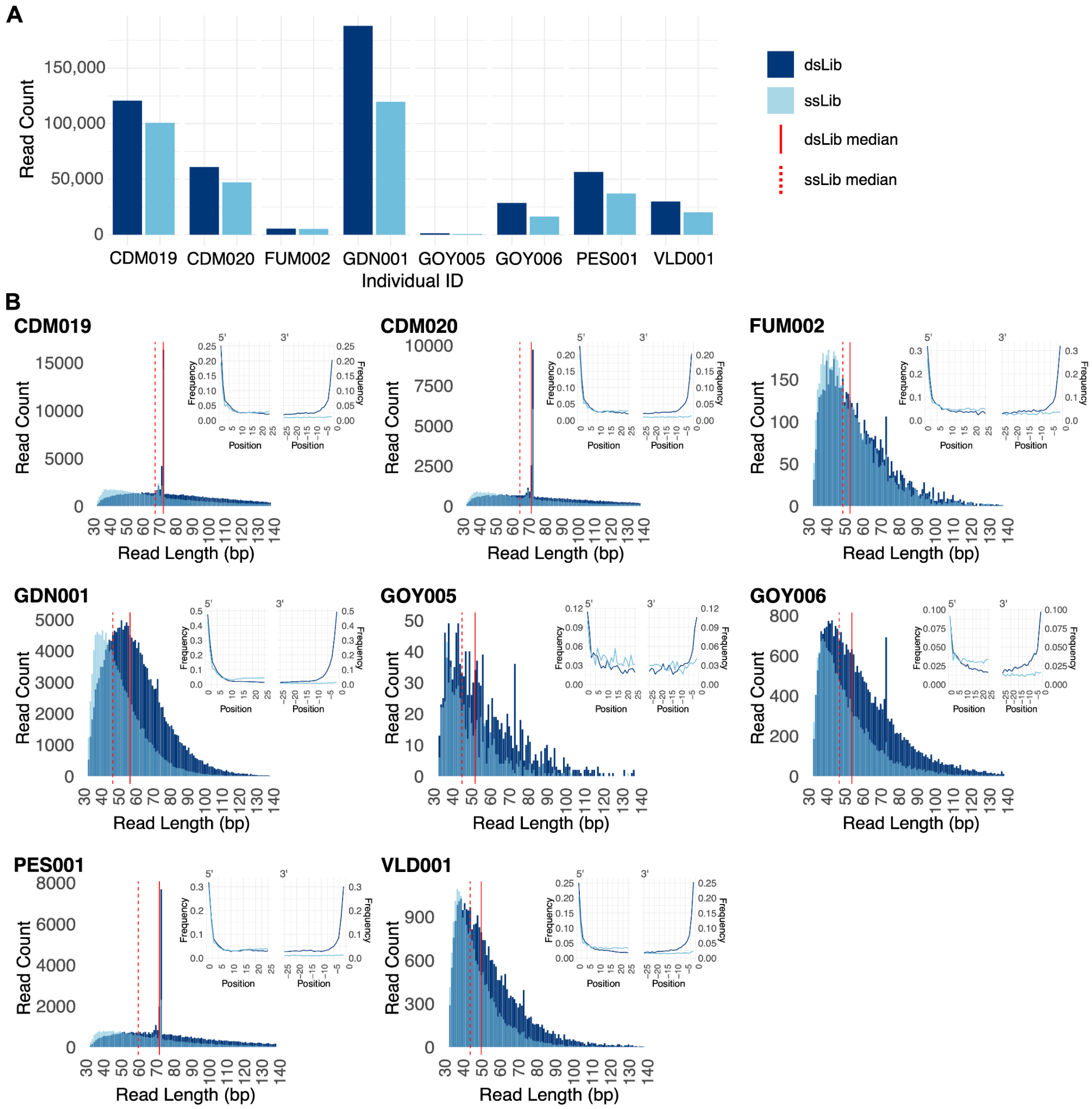

Supplemental Figure 31. *Flexilinea* sp001717545 (55% GC, mid GC taxa) per sample read length distributions and damage.

A. Bar plot displaying the total number of *Flexilinea* sp001717545 (*Anaerolineaceae* bacterium oral taxon 439) reads per well-preserved library subsampled to 5 million reads prior to taxonomic profiling (NPost). Only samples with over 100 reads were included. **B.** Histograms showing the distribution of read length for all *Flexilinea* sp001717545 reads for well-preserved samples subsampled to 5 million reads prior to taxonomic profiling. Plots are grouped by individual. The solid red line indicates the dsLib median while the dashed line indicates the ssLib median. Insets to the right of each histogram show nucleotide misincorporation patterns to assess characteristic aDNA damage. The left panel displays the frequency of cytosine deamination at the 5′ end (C→T), and the right panel shows the complementary guanine to adenine (G→A) transitions at the 3′ end. Double-stranded libraries exhibit complementary G→A substitutions at the 3′ end due to blunt-end repair during library preparation (Briggs et al., 2007). In contrast, single-stranded libraries show complementary C→T patterns at both ends because the DNA molecules are fully denatured and reconstructed without fill-in or blunt-end repair (Meyer et al., 2012). As a result, ssLibs typically display a flat profile at the 3′ end when plotting G→A transitions, since this misincorporation pattern is not expected in ssLib preparation.

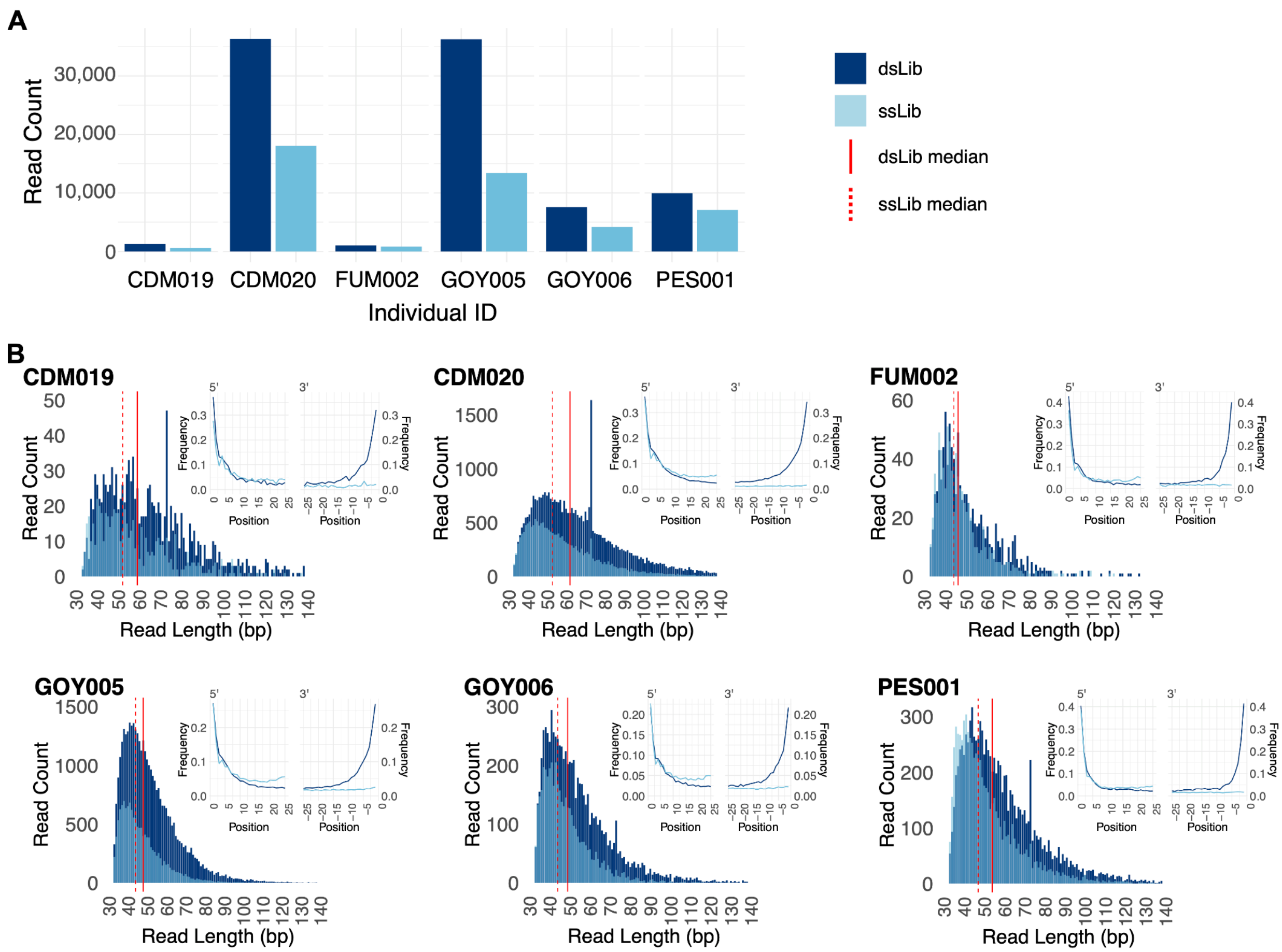

Supplemental Figure 32. *Lautropia mirabilis* (65.5% GC, high GC taxa) per sample read length distributions and damage.

A. Bar plot displaying the total number of *Lautropia mirabilis* reads per well-preserved library subsampled to 5 million reads prior to taxonomic profiling (NPost). Only samples with over 100 reads were included. **B.** Histograms showing the distribution of read length for all *L. mirabilis* reads for well-preserved samples subsampled to 5 million reads prior to taxonomic profiling. Plots are grouped by individual. The solid red line indicates the dsLib median while the dashed line indicates the ssLib median. Insets to the right of each histogram show nucleotide misincorporation patterns to assess characteristic aDNA damage. The left panel displays the frequency of cytosine deamination at the 5′ end (C→T), and the right panel shows the complementary guanine to adenine (G→A) transitions at the 3′ end. Double-stranded libraries exhibit complementary G→A substitutions at the 3′ end due to blunt-end repair during library preparation (Briggs et al., 2007). In contrast, single-stranded libraries show complementary C→T patterns at both ends because the DNA molecules are fully denatured and reconstructed without fill-in or blunt-end repair (Meyer et al., 2012). As a result, ssLibs typically display a flat profile at the 3′ end when plotting G→A transitions, since this misincorporation pattern is not expected in ssLib preparation.

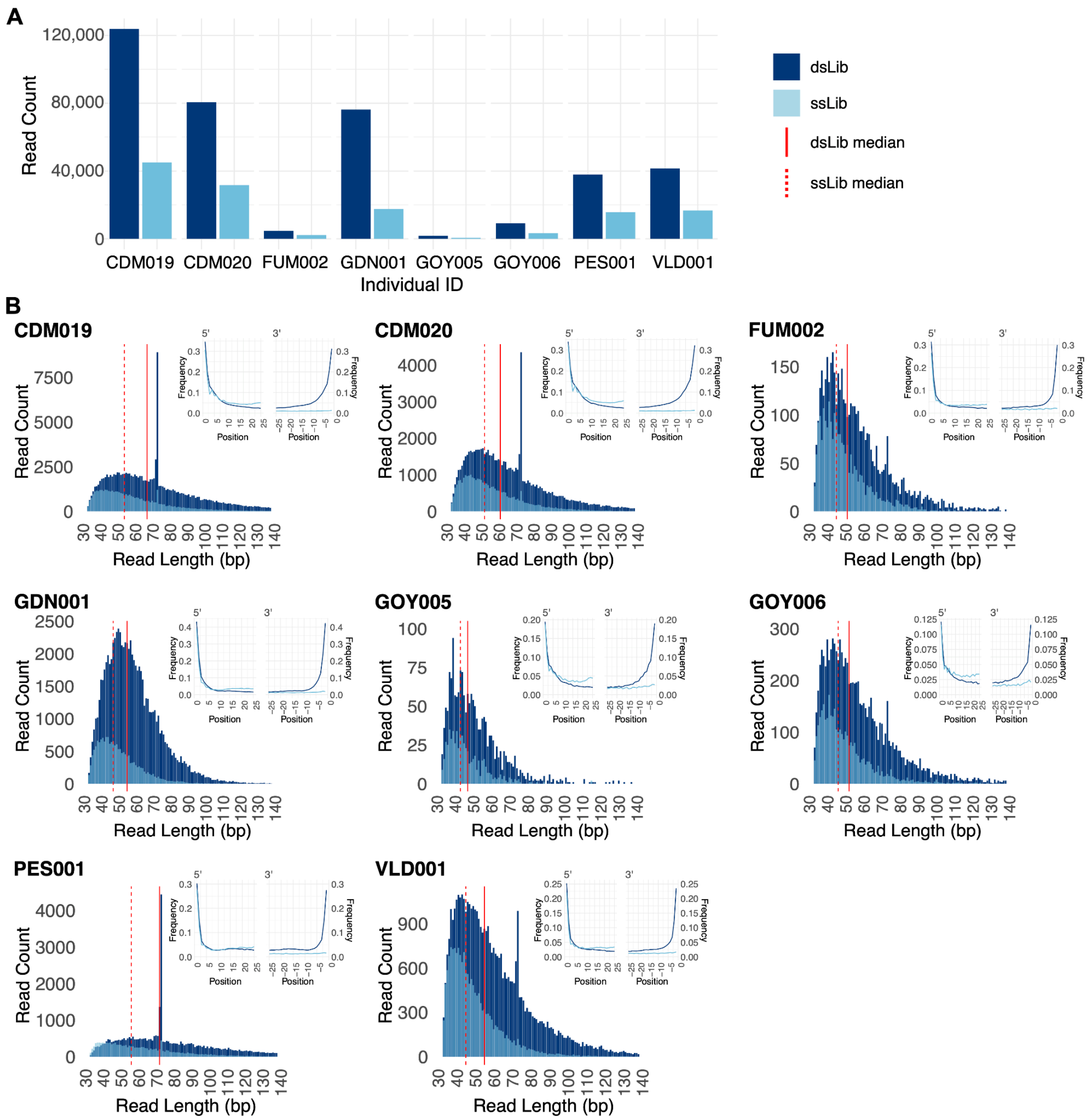

Supplemental Figure 33. ***Actinomyces dentalis*** **(73% GC, high GC taxa) per sample read length distributions and damage.**

A. Bar plot displaying the total number of *Actinomyces dentalis* reads per well-preserved library subsampled to 5 million reads prior to taxonomic profiling (NPost). Only samples with over 100 reads were included. **B.** Histograms showing the distribution of read length for all *A. dentalis* reads for well-preserved samples subsampled to 5 million reads prior to taxonomic profiling. Plots are grouped by individual. The solid red line indicates the dsLib median while the dashed line indicates the ssLib median. Insets to the right of each histogram show nucleotide misincorporation patterns to assess characteristic aDNA damage. The left panel displays the frequency of cytosine deamination at the 5′ end (C→T), and the right panel shows the complementary guanine to adenine (G→A) transitions at the 3′ end. Double-stranded libraries exhibit complementary G→A substitutions at the 3′ end due to blunt-end repair during library preparation (Briggs et al., 2007). In contrast, single-stranded libraries show complementary C→T patterns at both ends because the DNA molecules are fully denatured and reconstructed without fill-in or blunt-end repair (Meyer et al., 2012). As a result, ssLibs typically display a flat profile at the 3′ end when plotting G→A transitions, since this misincorporation pattern is not expected in ssLib preparation.

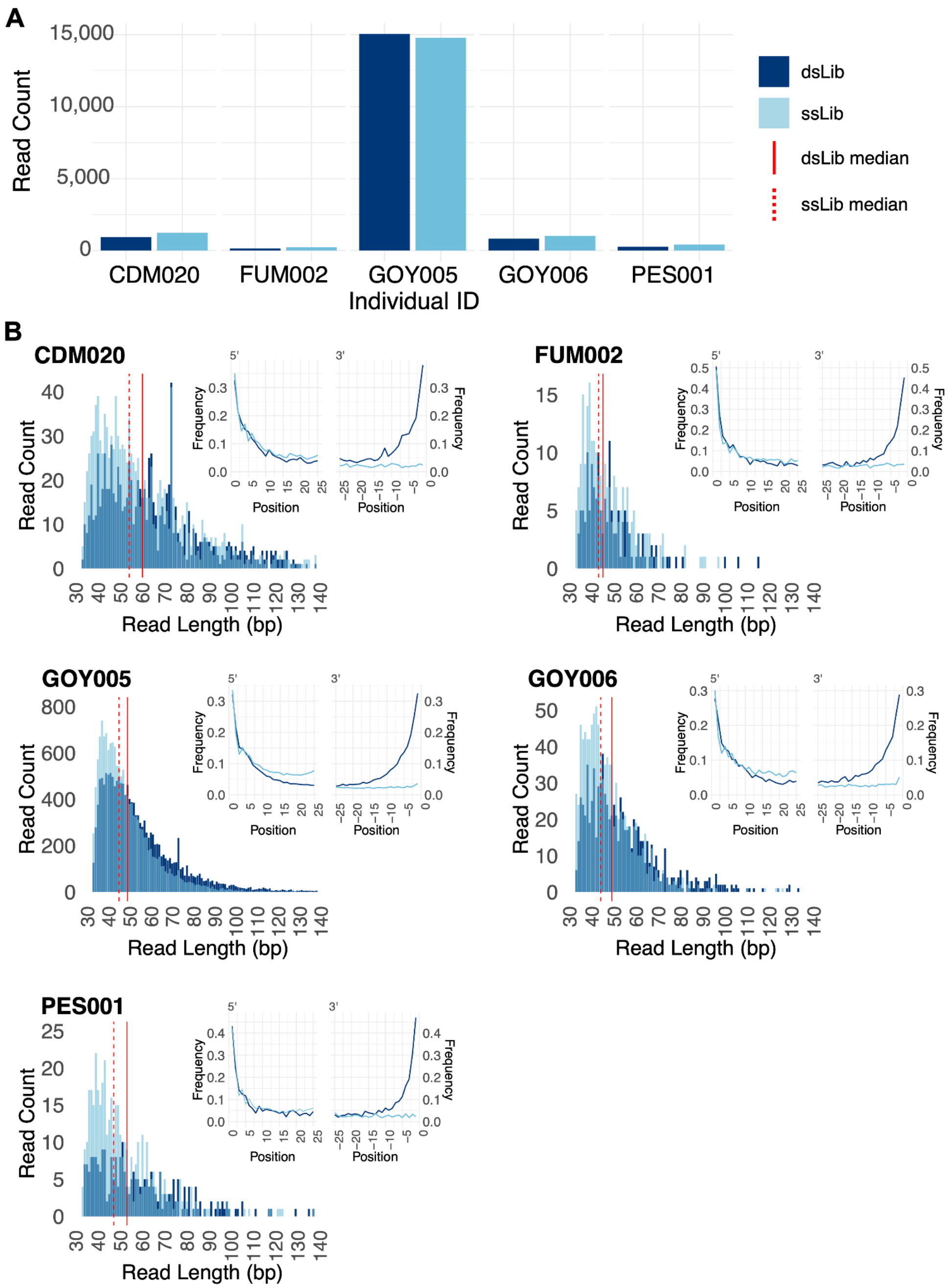

Supplemental Figure 34. *Aggregatibacter kilianii* (43% GC, low GC taxa) per sample read length distributions and damage.

A. Bar plot displaying the total number of *Aggregatibacter kilianii* reads per well-preserved library subsampled to 5 million reads prior to taxonomic profiling (NPost). Only samples with over 100 reads were included. **B.** Histograms showing the distribution of read length for all *A. kilianii* reads for well-preserved samples subsampled to 5 million reads prior to taxonomic profiling. Plots are grouped by individual. The solid red line indicates the dsLib median while the dashed line indicates the ssLib median. Insets to the right of each histogram show nucleotide misincorporation patterns to assess characteristic aDNA damage. The left panel displays the frequency of cytosine deamination at the 5′ end (C→T), and the right panel shows the complementary guanine to adenine (G→A) transitions at the 3′ end. Double-stranded libraries exhibit complementary G→A substitutions at the 3′ end due to blunt-end repair during library preparation (Briggs et al., 2007). In contrast, single-stranded libraries show complementary C→T patterns at both ends because the DNA molecules are fully denatured and reconstructed without fill-in or blunt-end repair (Meyer et al., 2012). As a result, ssLibs typically display a flat profile at the 3′ end when plotting G→A transitions, since this misincorporation pattern is not expected in ssLib preparation.

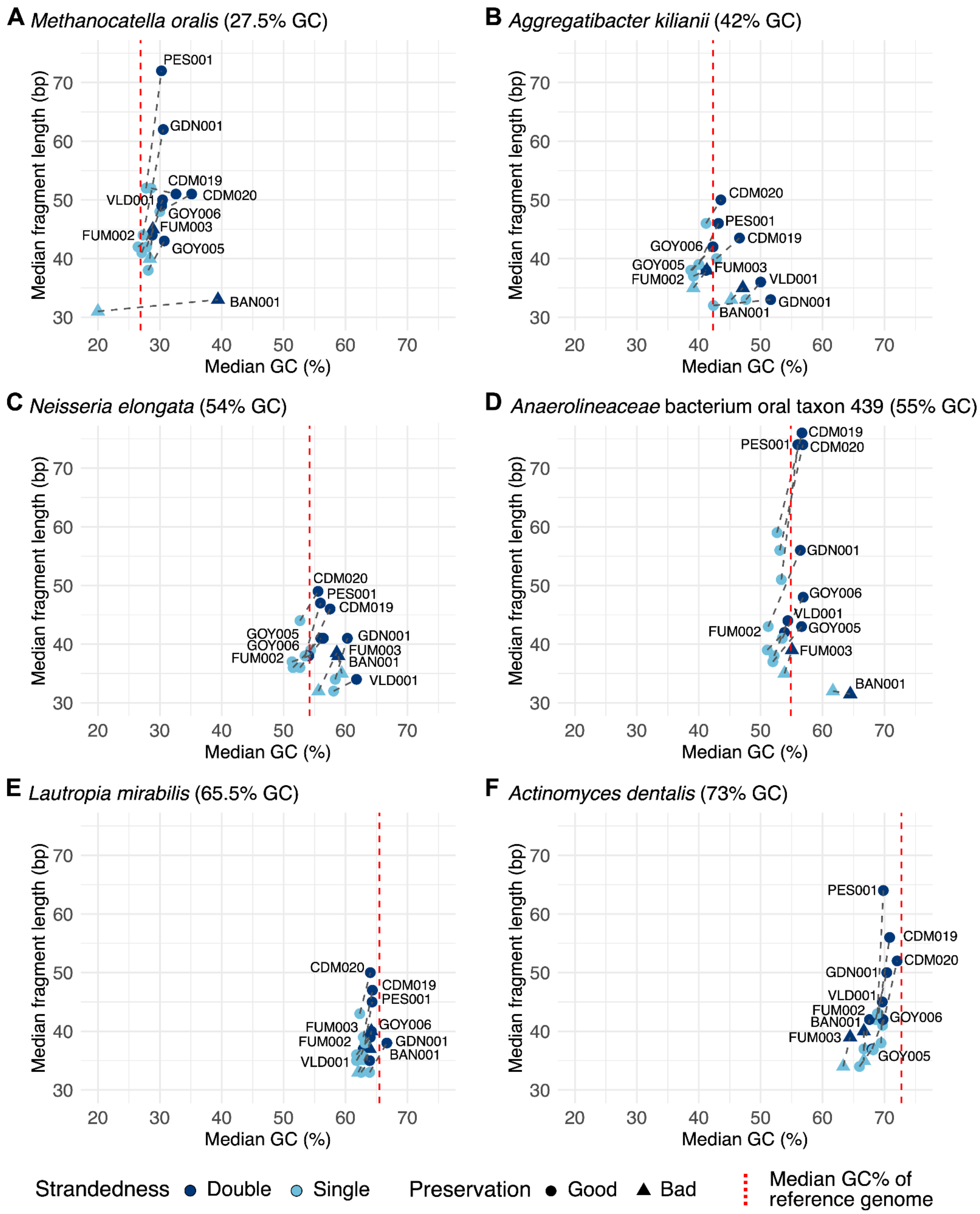

Supplemental Figure 35. Median GC% and read length of NPost mapped reads.

Plots showing the median GC content (%) and fragment length (bp) of reads mapped to the following species: **(A)** *Methanocatella oralis*, **(B)** *Aggregatibacter kilianii* **(C)** *Neisseria elongata*, **(D)** *Flexilinea* sp001717545 (*Anaerolineaceae* bacterium oral taxon 439), **(E)** *Lautropia mirabilis*, and **(F)** *Actinomyces dentalis*. The average GC content (%) is displayed next to each species name, and plots are arranged in order of ascending GC. The red dashed line on the plot displays the median GC% of each reference genome. Library type is denoted by color and preservation is denoted by shape. Grey lines connect libraries from the same individual, and individual names are placed next to the dsLib in the pair.

3.8 Clonality

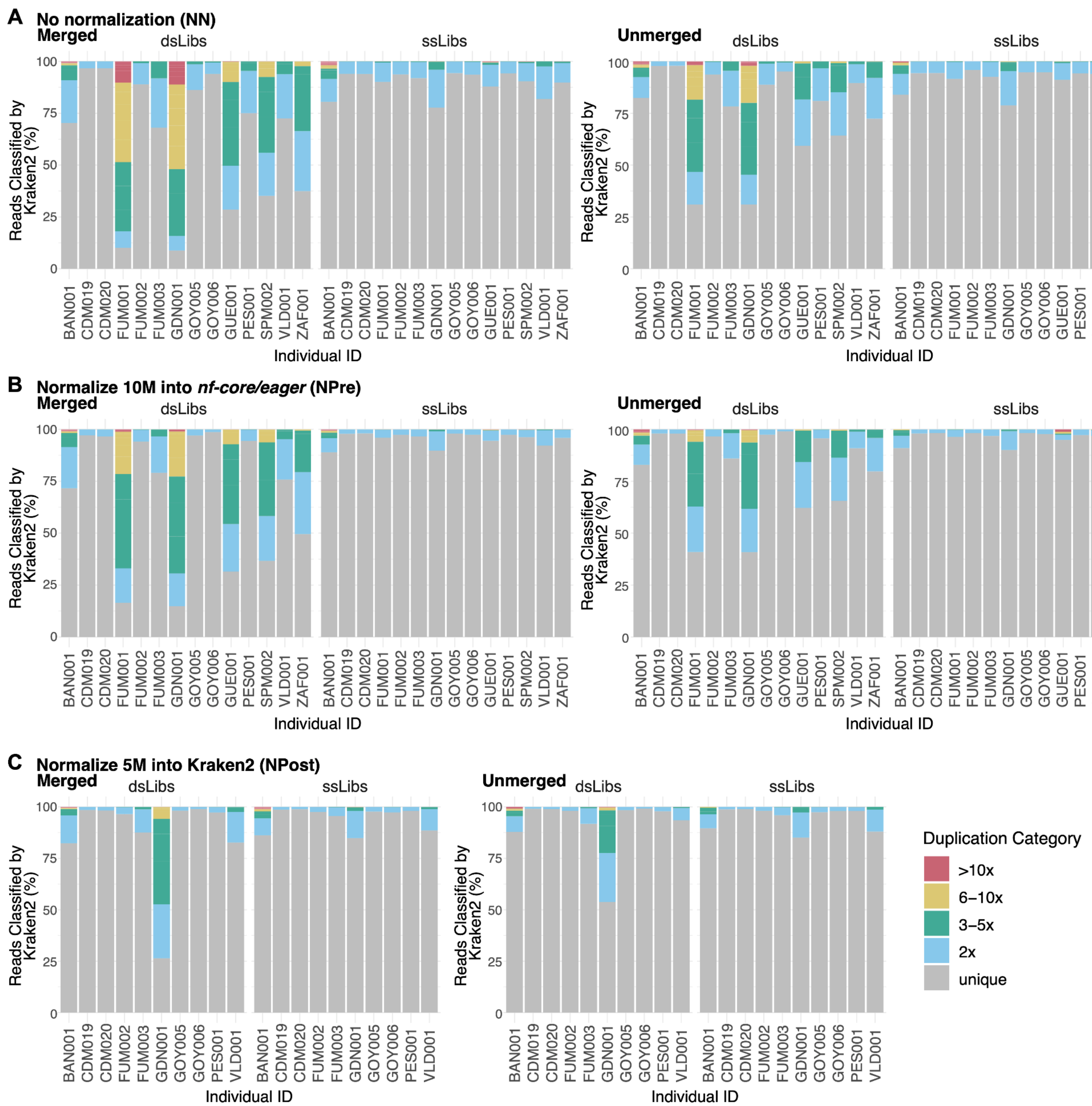

Supplemental Figure 36. Percent of duplicated reads classified by Kraken2.

Bar plots displaying the percentage of reads classified by Kraken2 that are duplicated. Duplication is separated into broader categories (unique/no duplication, duplicated twice, duplicated 3-5 times, duplicated 6-10 times, or duplicated greater than 10 times). Plots are divided between merged and unmerged reads for the no normalization (NN) dataset **(A)**, normalizing 10 million reads prior to processing (NPre) **(B)**, and normalizing 5 million reads prior to taxonomic profiling (NPost) **(C)**.

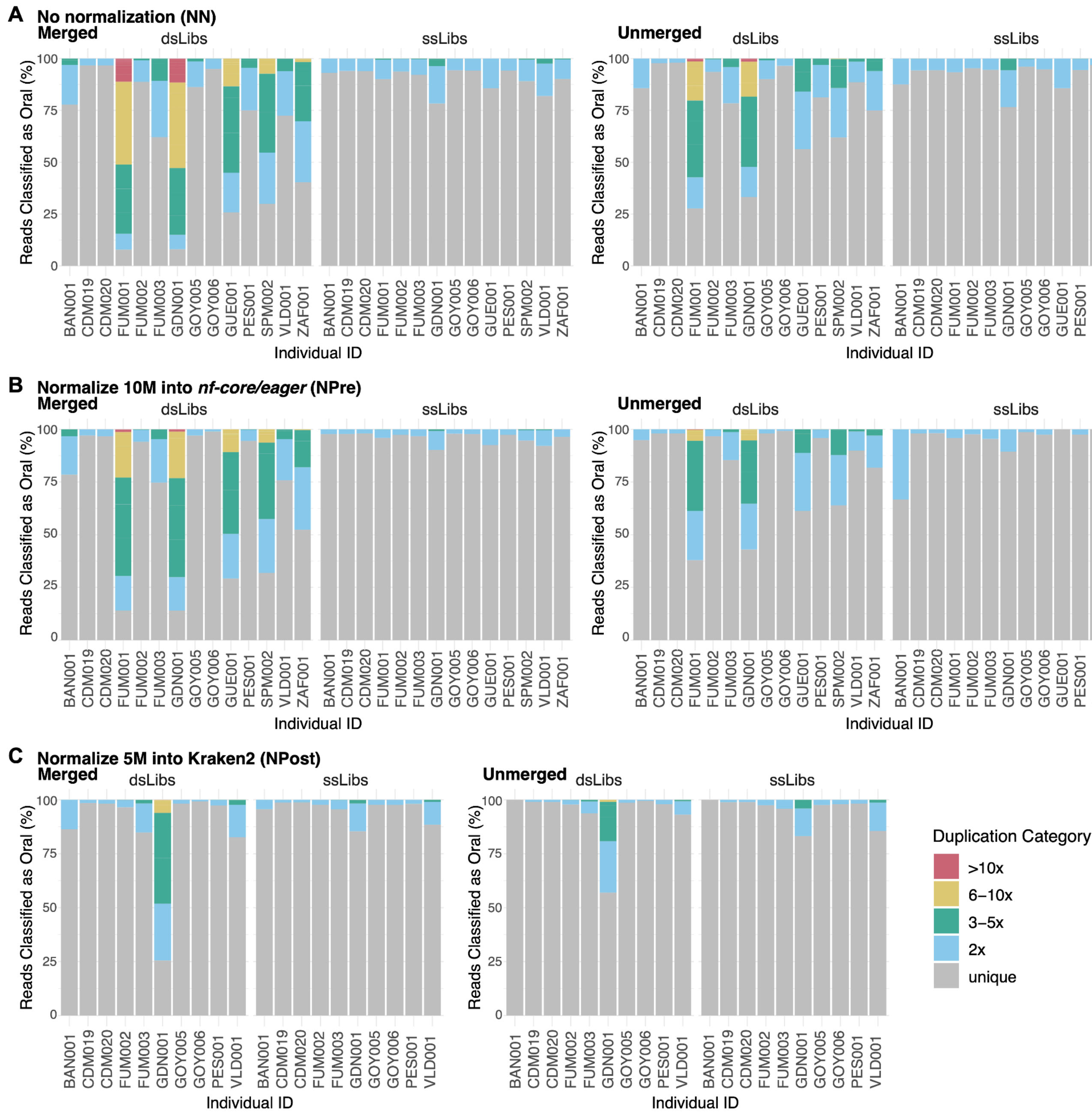

Supplemental Figure 37. Percent of duplicated oral reads.

Bar plots displaying the percentage of oral reads that are duplicated. Duplication is separated into broader categories (unique/no duplication, duplicated twice, duplicated 3-5 times, duplicated 6-10 times, or duplicated greater than 10 times). Plots are divided between merged and unmerged reads for the no normalization (NN) dataset **(A)**, normalizing 10 million reads prior to processing (NPre) **(B)**, and normalizing 5 million reads prior to taxonomic profiling (NPost) **(C)**.

References

Briggs, A. W., Stenzel, U., Johnson, P. L. F., Green, R. E., Kelso, J., Prüfer, K., Meyer, M., Krause, J., Ronan, M. T., Lachmann, M., & Pääbo, S. (2007). Patterns of damage in genomic DNA sequences from a Neandertal. *Proceedings of the National Academy of Sciences of the United States of America*, *104*(37), 14616–14621.

Meyer, M., Kircher, M., Gansauge, M.-T., Li, H., Racimo, F., Mallick, S., Schraiber, J. G., Jay, F., Prüfer, K., de Filippo, C., Sudmant, P. H., Alkan, C., Fu, Q., Do, R., Rohland, N., Tandon, A., Siebauer, M., Green, R. E., Bryc, K., … Pääbo, S. (2012). A high-coverage genome sequence from an archaic Denisovan individual. *Science (New York, N.Y.)*, *338*(6104), 222–226.

Oksanen, J., Simpson, G. L., Blanchet, F. G., Kindt, R., Legendre, P., Minchin, P. R., O’Hara, R. B., Solymos, P., Stevens, M. H. H., Szoecs, E., Wagner, H., Barbour, M., Bedward, M., Bolker, B., Borcard, D., Borman, T., Carvalho, G., Chirico, M., De Caceres, M., … Weedon, J. (2025). *vegan: Community Ecology Package*. https://vegandevs.github.io/vegan/
